# SMOC2 promotes partial epithelial-mesenchymal transition and maladaptive repair in renal tubular epithelial cells

**DOI:** 10.1172/jci.insight.198555

**Published:** 2026-08-11

**Authors:** Schrodinger Cenatus, Peng Gao, Nathalie Henley, Caroline Lamarche, Xue-Song Liu, Frédérick A. Mallette, Jonatan Barrera-Chimal, Casimiro Gerarduzzi

**Affiliations:** 1 Maisonneuve-Rosemont Hospital Research Centre, Montreal, Canada.; 2 Programmes de biologie moléculaire, Faculté de médecine,; 3 Département de Pharmacologie et Physiologie, and; 4 Département de Médecine, Faculté de Médecine, Université de Montréal, Succ. Centre-Ville, Montreal, Quebec, Canada.; 5 School of Life Science and Technology, ShanghaiTech University, Shanghai, China.

**Keywords:** Cell biology, Nephrology, Chronic kidney disease, Extracellular matrix, Fibrosis

## Abstract

Chronic kidney disease is a global health concern characterized by maladaptive repair processes that lead to kidney fibrosis. Following injury, early alterations in the extracellular matrix precede the development of kidney fibrosis and represent potential therapeutic targets to improve kidney repair. In this context, studies from our laboratory and others have shown that the matricellular protein SMOC2 can be targeted to decrease inflammation and tubulointerstitial fibrosis after kidney injury. Tubular epithelial cells (TECs), which are abundant and particularly susceptible to injury, play a central role in maladaptive repair; however, whether SMOC2 affects their function after kidney injury has not been explored. In this study, we showed that SMOC2 localized to the basement membrane of injured TECs across 3 murine models of kidney injury. Our in vitro studies demonstrate that SMOC2 induced a partial epithelial-to-mesenchymal (EMT) transition in TECs. We further demonstrated that its extracellular calcium-binding domain mediated binding to the decellularized extracellular matrix and accounted for most of its effects on TECs. Mechanistically, SMOC2 promoted partial EMT through an integrin-dependent pathway. Together, these findings provide mechanistic insight into how SMOC2 drives maladaptive repair by modulating TEC behavior and identify its calcium-binding domain as a key functional mediator.

## Abstract 2

The protein SMOC2 worsens kidney scarring by changing injured kidney cells and activating nearby scar-forming cells, revealing a possible treatment target.

## Graphical abstract

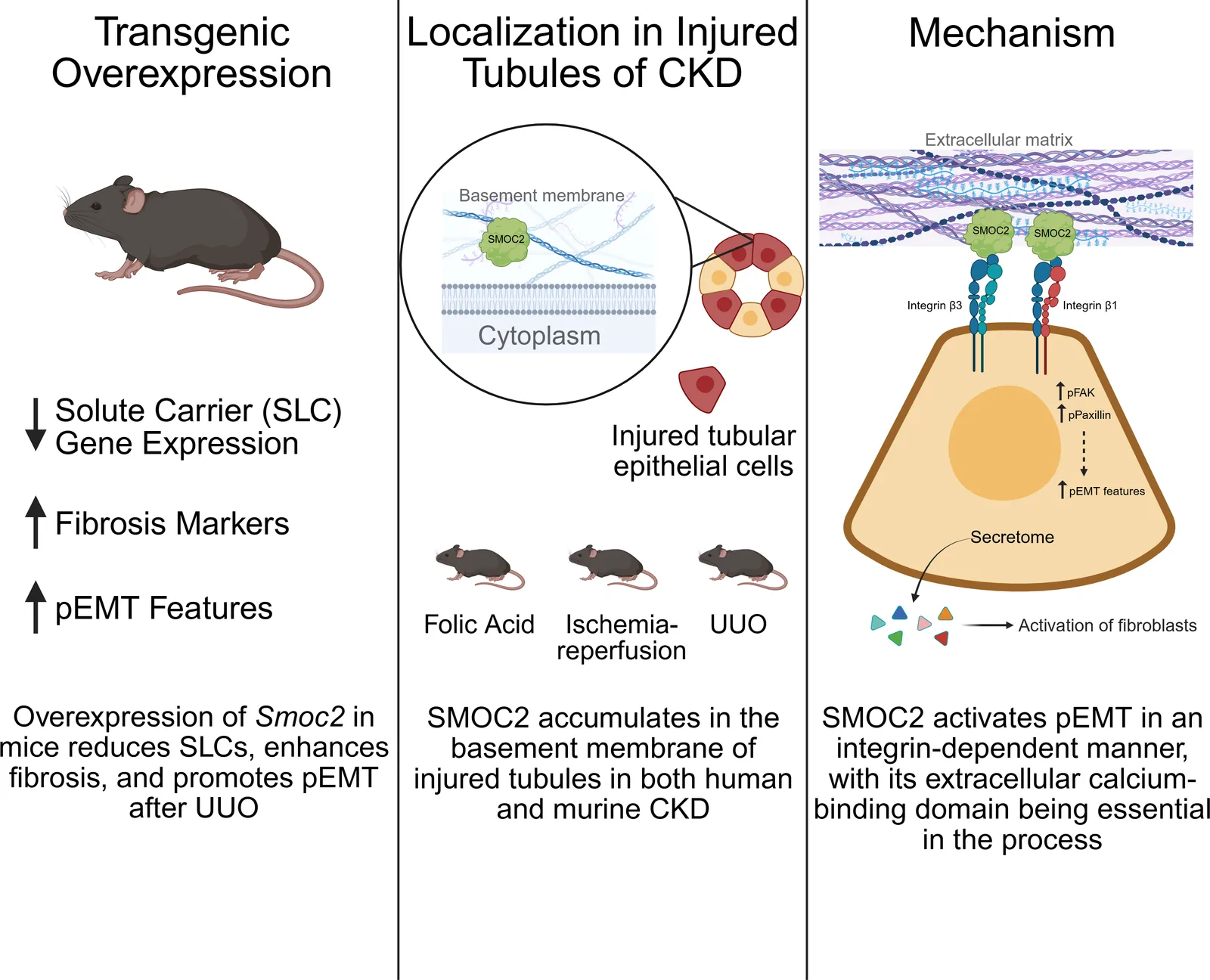

## Introduction

Chronic kidney disease (CKD) is a progressive condition affecting more than 10% of the global population (1, 2). Its etiologies are diverse and include recurrent or severe renal injury (3, 4). Regardless of its causes, CKD culminates in kidney fibrosis, a consequence of maladaptive repair characterized by excessive accumulation of extracellular matrix (ECM) proteins (5, 6). Although the provisional matrix initially maintains kidney integrity after injury, its progression into fibrotic tissue disrupts the parenchymal structure and function, leading to nephron loss and organ failure (5, 7). Thus, understanding maladaptive repair mechanisms could lead to the development of novel therapies that limit fibrosis and prevent end-stage renal disease.

The maladaptive repair response leading to kidney fibrosis involves parenchymal cells, such as tubular epithelial cells (TECs), and nonparenchymal cells, such as fibroblasts and immune cells (5, 7). Among these cell types, TECs, the principal kidney cells, are known for their susceptibility to injury due to their exposure to toxins during solute reabsorption and their high metabolic activity (3, 4, 8, 8–11). They have gained interest for their prominent role in initiating and orchestrating kidney repair events (8, 10, 12). During maladaptive repair, these cells dedifferentiate and acquire mesenchymal features, including increased motility and upregulation of mesenchymal and proinflammatory genes (11, 13–17). However, lineage-tracing experiments have shown that dedifferentiated TECs do not translocate into the interstitial space but remain attached to their basement membrane (18–21). Consequently, the term partial epithelial-mesenchymal transition (pEMT) was proposed to denote this process (16). Studies modulating the activities of transcription factors such as Snai1 and Twist have demonstrated that mesenchymal repression ameliorates renal inflammation and fibrosis in several models of kidney injury (13, 15, 16, 22–27). These studies highlight that persistence in the pEMT phenotype is a conserved mechanism driving kidney function loss and fibrosis in diverse types of CKD.

The ECM has been studied in the context of advanced disease, where its macroscopic alterations signal the onset of fibrosis. However, recent proteomic analyses have revealed that ECM remodeling begins much earlier in kidney disease, even before overt structural changes are detectable (28, 29). The ECM was found to be pivotal in modulating critical pathways that regulate maladaptive repair and establishing fibrogenic niches, which are localized regions where fibrosis is initiated (5, 9, 28–30). Proteomic analyses of decellularized kidney tissue scaffolds from normal and fibrotic kidneys revealed several deregulated ECM components, such as elevated levels of matricellular proteins (MCPs) (31). The MCPs are nonstructural matrix proteins that modulate tissue remodeling, cell behavior, and interactions within the ECM (9, 30, 32). These proteomic data also confirm RNA-seq findings on the deregulated expression of several MCP-coding genes in diverse CKD models (32). Together, these studies suggest an important role for MCPs in regulating repair after injury, which, if left unregulated, can become maladaptive to the point of fibrogenesis.

SPARC-related modular calcium-binding protein 2 (SMOC2) is an MCP whose gene is localized on the human chromosome 6 (33). SMOC2 is structurally composed of 5 domains, which are a follistatin-like (FS) domain, 2 thyroglobulin (TY) type-I domains, a calcium-binding domain with 2 EF-hands, and a SMOC-specific domain (34). SMOC2 is implicated in several diseases, including renal cell carcinoma (33), endometrial carcinoma (35), pulmonary fibrosis (36), and CKD (37–39). Its expression is upregulated in CKD (32), with plasma levels positively correlating with the severity of interstitial fibrosis (37). Multiple studies using various CKD models have shown that *Smoc2*-deficient mice are protected against kidney function loss and exhibit reduced fibrosis and fibroblast activation (38, 39). These findings highlight the involvement of SMOC2 in maladaptive repair following kidney injury. Whether SMOC2 affects TEC function during this maladaptive repair remains unexplored. In this study, we leveraged RNA-seq data to uncover cellular functions of SMOC2 during CKD, and we examined its effects in TECs in vitro. We explored the localization of SMOC2 regarding the kidney tubules following unilateral ureteral obstruction (UUO), folic acid nephropathy, and ischemia-reperfusion injury. We also investigated the various domains of SMOC2 in mediating its effects on TECs and identified the associated signaling pathways, providing a comprehensive mechanism by which this MCP influences kidney epithelial cell repair.

## Results

### Persistent Smoc2 expression during UUO aggravates maladaptive TEC repair and renal fibrosis.

**Figure 1:**
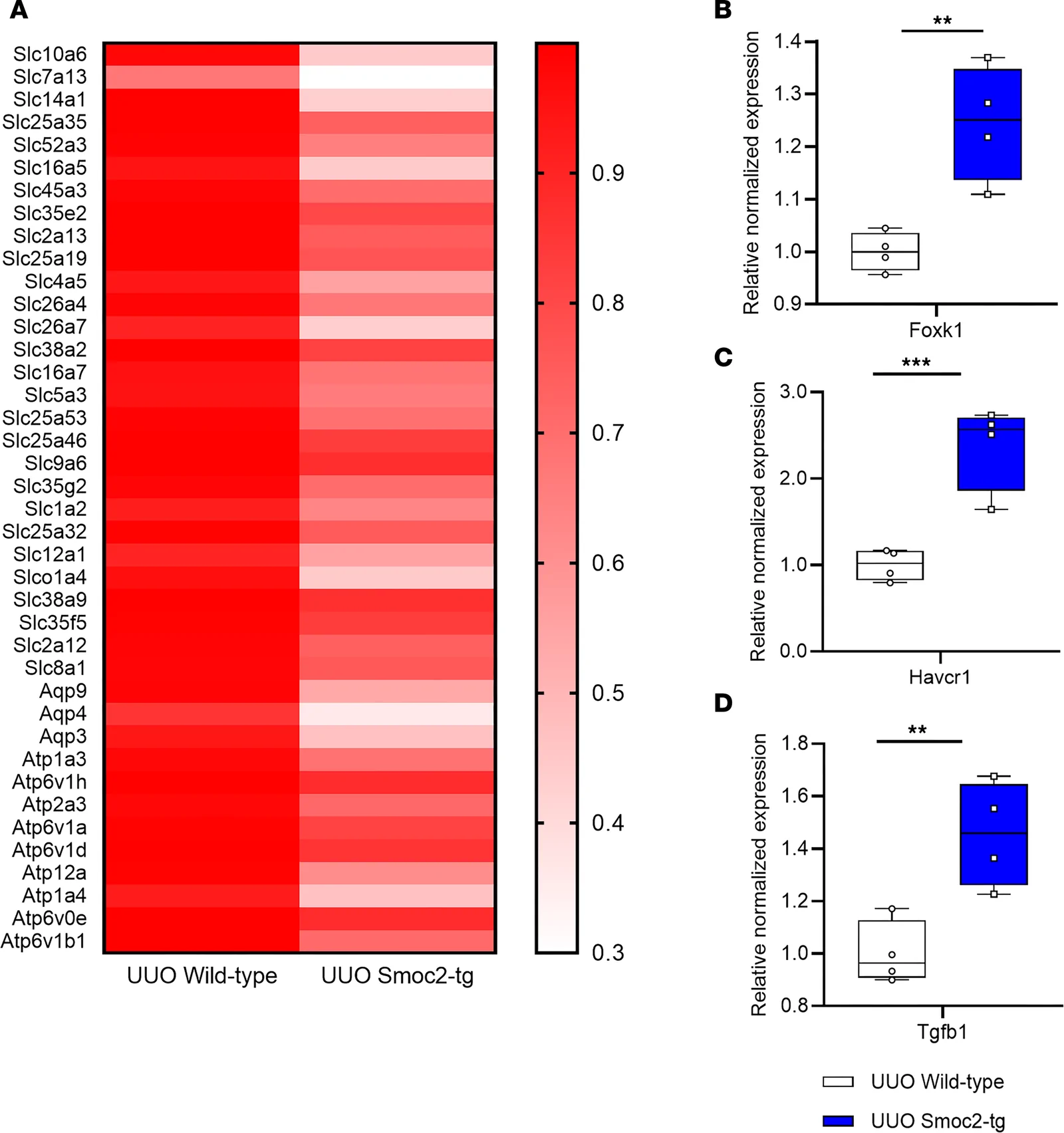
Smoc2-transgenic mice exhibit maladaptive tubular epithelial cell dedifferentiation and exacerbated kidney injury after UUO. (**A**) Heatmap displaying the relative expression of kidney epithelial transporter genes in Smoc2-transgenic (Smoc2-tg) mice compared with WT littermates 7 days after UUO. (**B**–**D**) Box plots showing the relative expression of the transcription factor *Foxk1* (**B**), the proximal kidney injury gene *Havcr1* (**C**), and the fibrotic gene *Tgfb1* in Smoc2-tg and WT mice after UUO (**D**). Data are presented as mean ± SD, and adjusted *P* values (FDR) are shown on box plots; *n* = 4, **P < 0.01, ***P < 0.001

**Figure 2:**
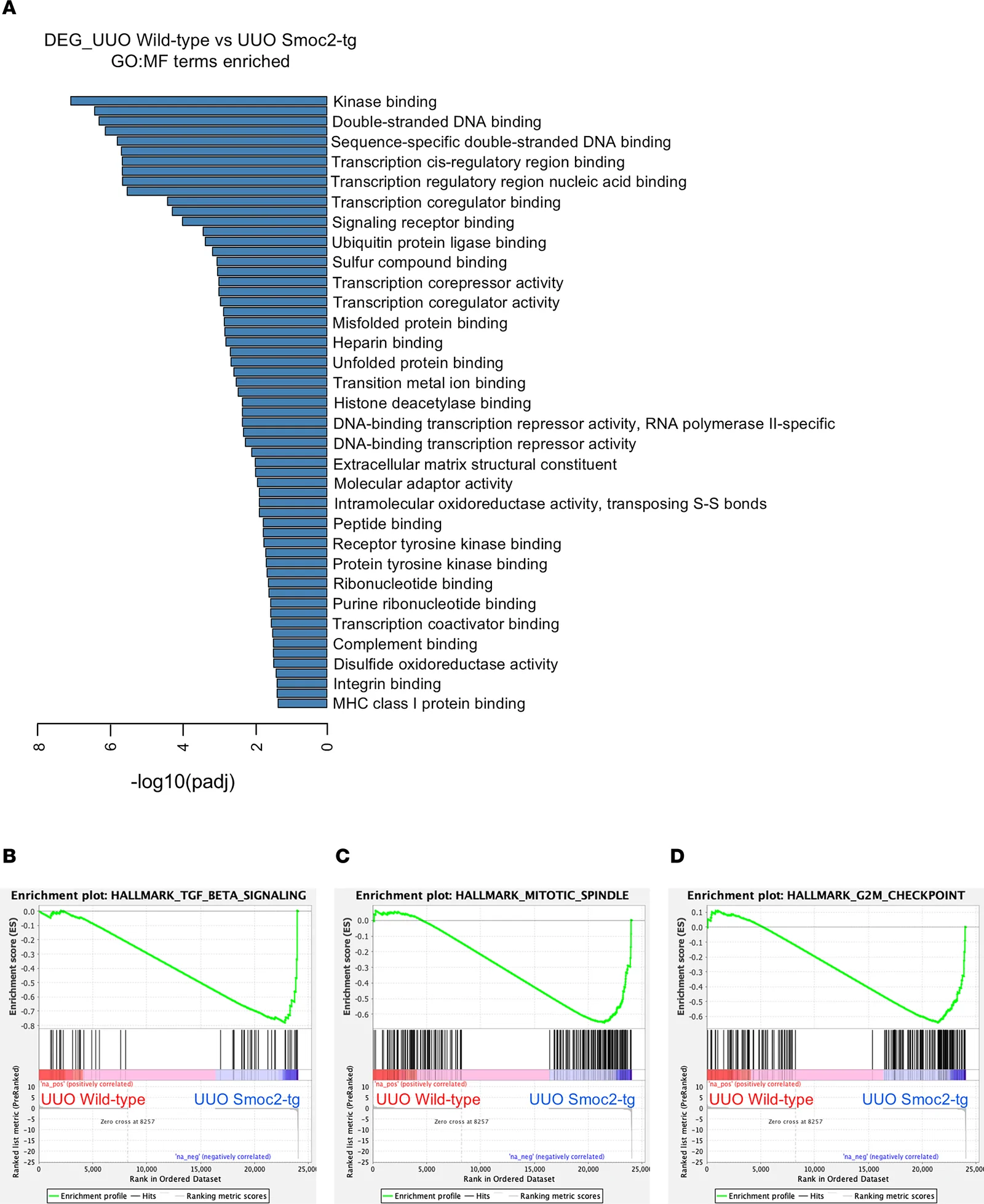
Smoc2-transgenic mice dysregulated pathways involved in pEMT after kidney injury. (**A**) Bar plots showing the gene ontology molecular function (GO:MF) analysis of the differentially expressed genes (DEGs) in Smoc2-tg mice compared with WT mice after UUO. (**B**–**D**) Gene set enrichment analyses showing decreased TGF-β signaling (**B**), mitotic spindle (**C**), and G2M checkpoint (**D**) in WT mice compared with Smoc2-tg mice after UUO.

Transgenic mice overexpressing *Smoc2* (Smoc2-tg), with a 2.5-fold increase in SMOC2 protein, are more prone to kidney injury and fibrosis (39). To investigate this in renal tubules, we examined RNA-seq data from Smoc2-tg and WT mice after 7 days of UUO. Compared with WT, Smoc2-tg mice showed decreased expression of kidney transporter genes, including solute carriers (SLC), aquaporins, and ATPases (Figure 1A). Some transporters were also increased in Smoc2-tg mice (Supplemental Figure 1A; supplemental material available online with this article; https://doi.org/10.1172/jci.insight.198555DS1). Notably, *Slc52a2* and *Slc1a5* — upregulated in cancers and pulmonary fibrosis, respectively — were among the most upregulated (40, 41). Additionally, Smoc2-tg mice showed increased *Foxk1* expression (Figure 1B), which has a profibrotic role in proximal tubules after injury (42, 43). They also showed more kidney injury and fibrosis, with higher *Havcr1* (KIM-1) and *Tgfb1* levels (Figure 1, C and D). Gene ontology molecular function (GO:MF) analysis of differentially expressed genes (DEGs) revealed affected functions, including kinase binding, DNA binding, transcription regulation, ECM, and integrin binding (Figure 2A). Gene set enrichment analysis showed that TGF-β signaling was negatively enriched in WT mice compared with Smoc2*-*tg mice (Figure 2B). WT mice undergoing UUO also showed negative enrichment for the mitotic spindle and G2/M checkpoint compared with Smoc2-tg mice (Figure 2, C and D), suggesting increased cell accumulation in the G2/M phase after injury.

### SMOC2 expression and localization are associated with tubular injury, pEMT, and fibrosis in UUO, ischemia-reperfusion injury, and folic acid nephropathy.

**Figure 3:**
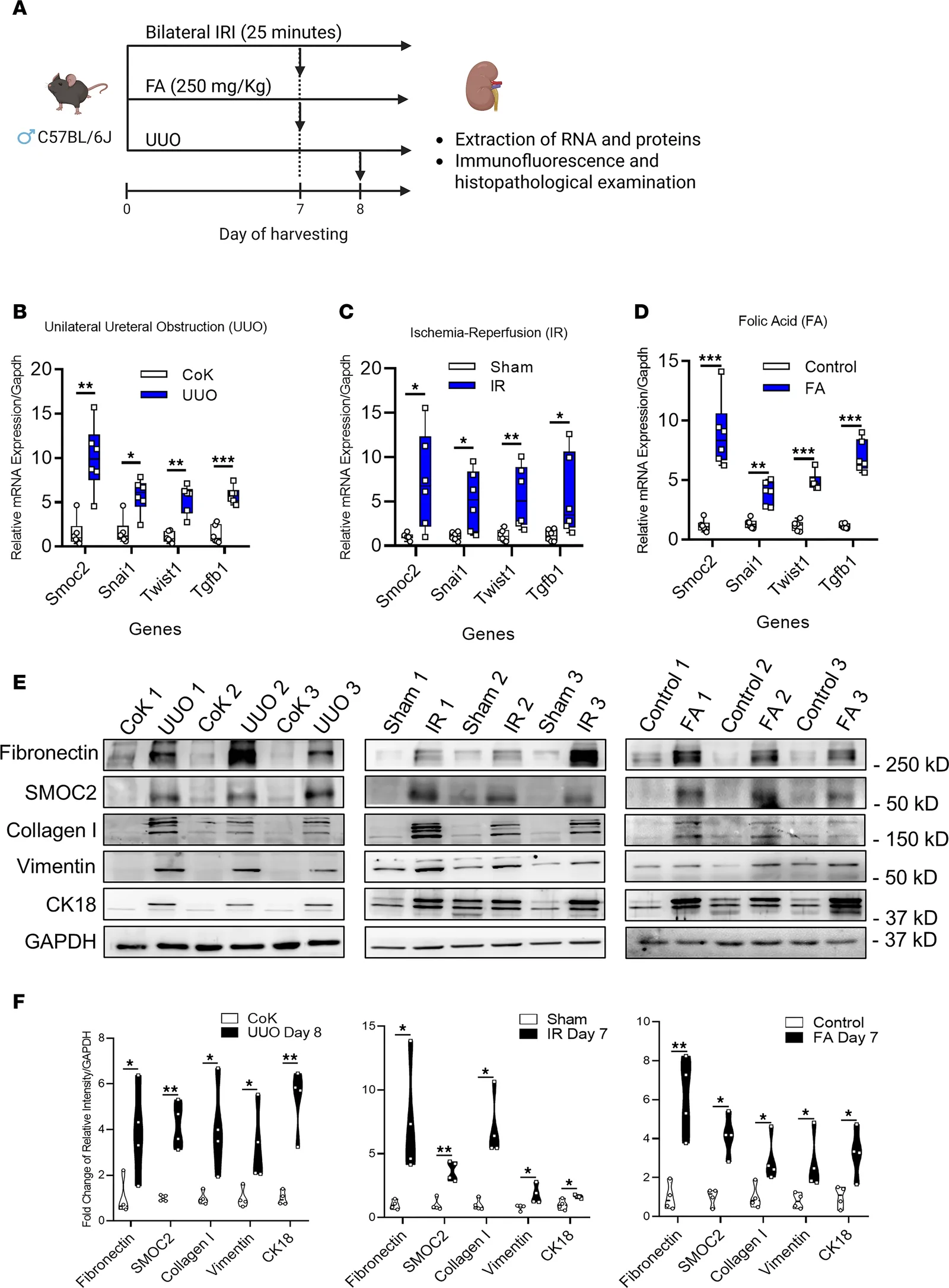
SMOC2 upregulation is concomitant with pEMT, injury, and fibrotic markers in obstructive, ischemic, and toxicant models of kidney injury. (**A**) Diagram depicting the study design for the murine kidney injury models. Male C57BL/6J mice were subjected to bilateral ischemia-reperfusion injury for 25 minutes, folic acid injection (250 mg/kg), or UUO. Mice were euthanized after 8 days for UUO and after 7 days for the other models before tissue collection and paraffin embedding. (**B**–**D**) Box plots showing the gene expression by qPCR of *Smoc2*, the pEMT transcription factors *Snai1* and *Twist1*, and the fibrotic gene *Tgfb1* in UUO, folic acid (FA) nephropathy, and ischemia-reperfusion (IR) injury, respectively. The expression of each gene was normalized by the expression of the housekeeping gene *Gapdh*. Data are presented as mean ± SD. (**E**) Western blots showing the levels of SMOC2, fibrotic proteins (fibronectin and collagen I), pEMT, and injury proteins (vimentin and CK-18, respectively) in all 3 models of kidney injury. (**F**) Violin plots showing the quantification of the Western blots in **E**. CoK, Contralateral kidney. Unpaired 2-tailed Student’s *t* test with Welch’s correction. *P < 0.05, **P < 0.01, ***P < 0.001; *n* = 4–6 for each model.

**Figure 4:**
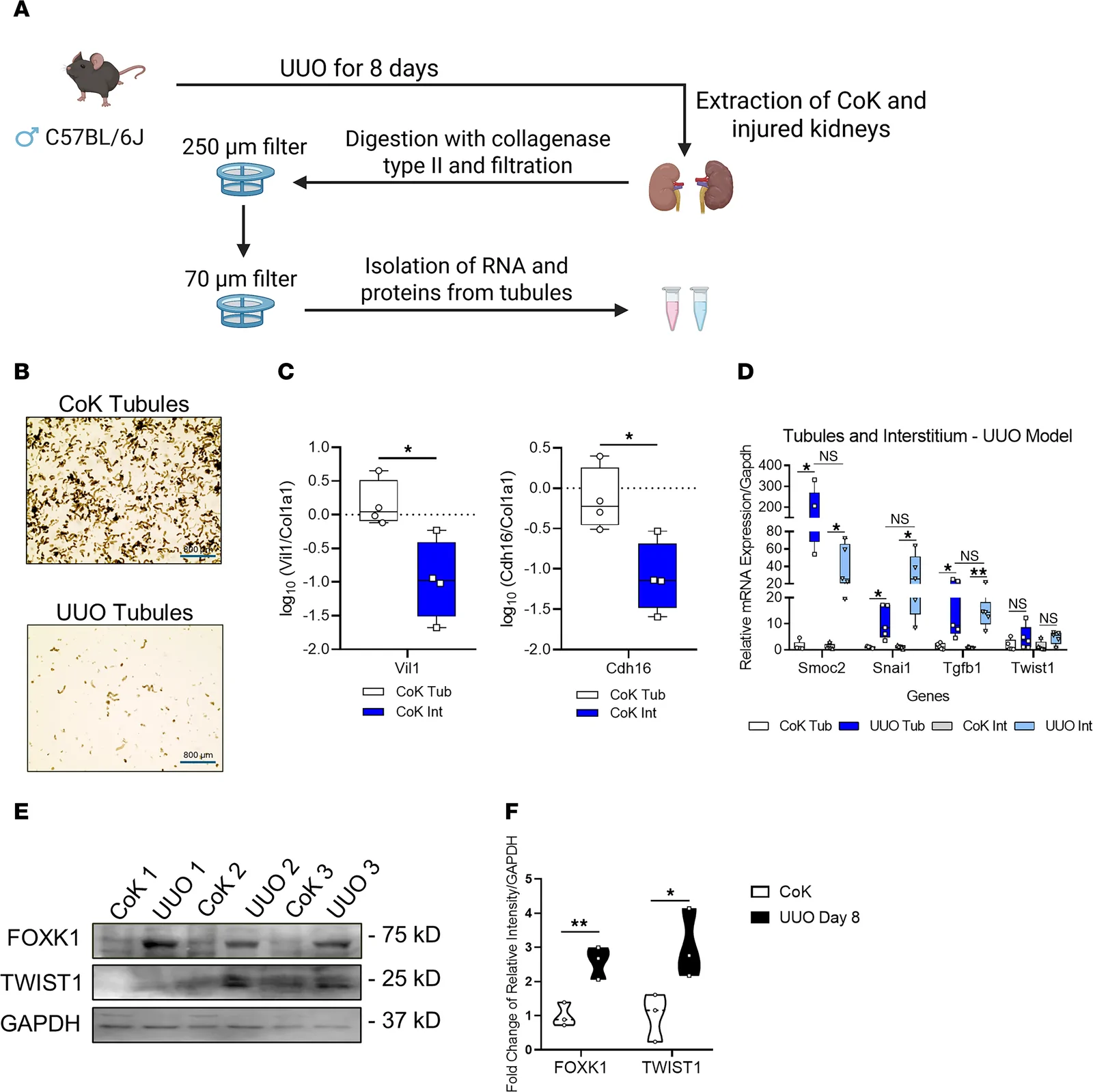
Injured tubules after UUO undergo pEMT and significantly contribute to *Smoc2* upregulation. (**A**) Diagram depicting the study design for the isolation of kidney tubules by size exclusion before qPCR and Western blot analyses. (**B**) Microscopic images of the isolated tubules from contralateral (CoK) and UUO kidneys after 8 days of injury. Images were taken using a 10× objective. (**C**) Box plots showing the enrichment for the ratio of epithelial genes (*Vil1* and *chd16*) over collagen I (*Col1a1*) for the tubules (Tub) and the interstitium (Int) following the extraction of tubules. Gene expression was measured by qPCR, and statistical tests were performed after log transformation. (**D**) Box plots showing the gene expression by qPCR of *Smoc2*, the pEMT transcription factors *Snai1* and *Twist1*, and the fibrotic gene *Tgfb1* in tubules (Tub) and interstitium (Int) from contralateral and UUO kidneys. The expression of each gene was normalized by the expression of the housekeeping gene *Gapdh*. Data are presented as mean ± SD. (**E**) Western blots showing the protein levels of pEMT transcription factors TWIST1 and FOXK1 in tubules from contralateral and UUO kidneys. (**F**) Violin plots showing the quantification of the Western blots in **E**. Unpaired 2-tailed Student’s *t* test with Welch’s correction (**C** and **F**), 1-way ANOVA (**D**). *P < 0.05, **P < 0.01; *n* = 3–4.

Loss of kidney transporters, inflammation, and fibrosis are linked to pEMT in CKD models (13, 16). The forkhead box protein K1 (FOXK1) regulates pEMT in tubular cells after injury (43). Additional analyses revealed that Smoc2-tg mice upregulate mesenchymal and fibrotic genes such as *Tnc*, *Snai1*, *Col1a1*, and *Mmp3* (Supplemental Figure 1B). We therefore examined SMOC2 in relation to pEMT and fibrosis in UUO, folic acid nephropathy, and ischemia-reperfusion injury (Figure 3A). Male C57BL/6J mice showed increased *Smoc2* after UUO, folic acid (FA), or ischemia-reperfusion (IR), along with higher *Snai1*, *Twist1*, and *Tgfb1* (Figure 3, B–D). Increased SMOC2, fibronectin, collagen I, vimentin, and the TEC injury marker CK-18 were confirmed in injured tissues (Figure 3, E and F). Although *Snai1* expression is mainly associated with tubules (13, 16, 44), genes such as *Twist1* (45, 46), *Tgfb1* (39), and *Smoc2* (38, 39) are also expressed in fibroblasts, contributing to their activation and inflammation. We isolated tubules in the UUO model to assess their contribution to these genes (Figure 4, A and B). Tubules are enriched for *Vil1* and *Cdh16* relative to *Col1a1*, confirming successful isolation (Figure 4C). Tubules from injured kidneys showed increased *Smoc2*, *Snai1*, and *Tgfb1*, with no significant difference compared with interstitial cells, while *Twist1* levels remained unchanged (Figure 4D). Considering RNA turnover after kidney injury is dynamic (47), and TWIST1 can be stabilized posttranslationally (47, 48), we assessed TWIST1 and FOXK1 protein levels in isolated tubules (Figure 4, E and F). Both proteins increased in injured tubules (Figure 4, E and F), confirming TECs as major contributors to the induction of *Smoc2*, mesenchymal, and profibrotic genes following injury.

**Figure 5:**
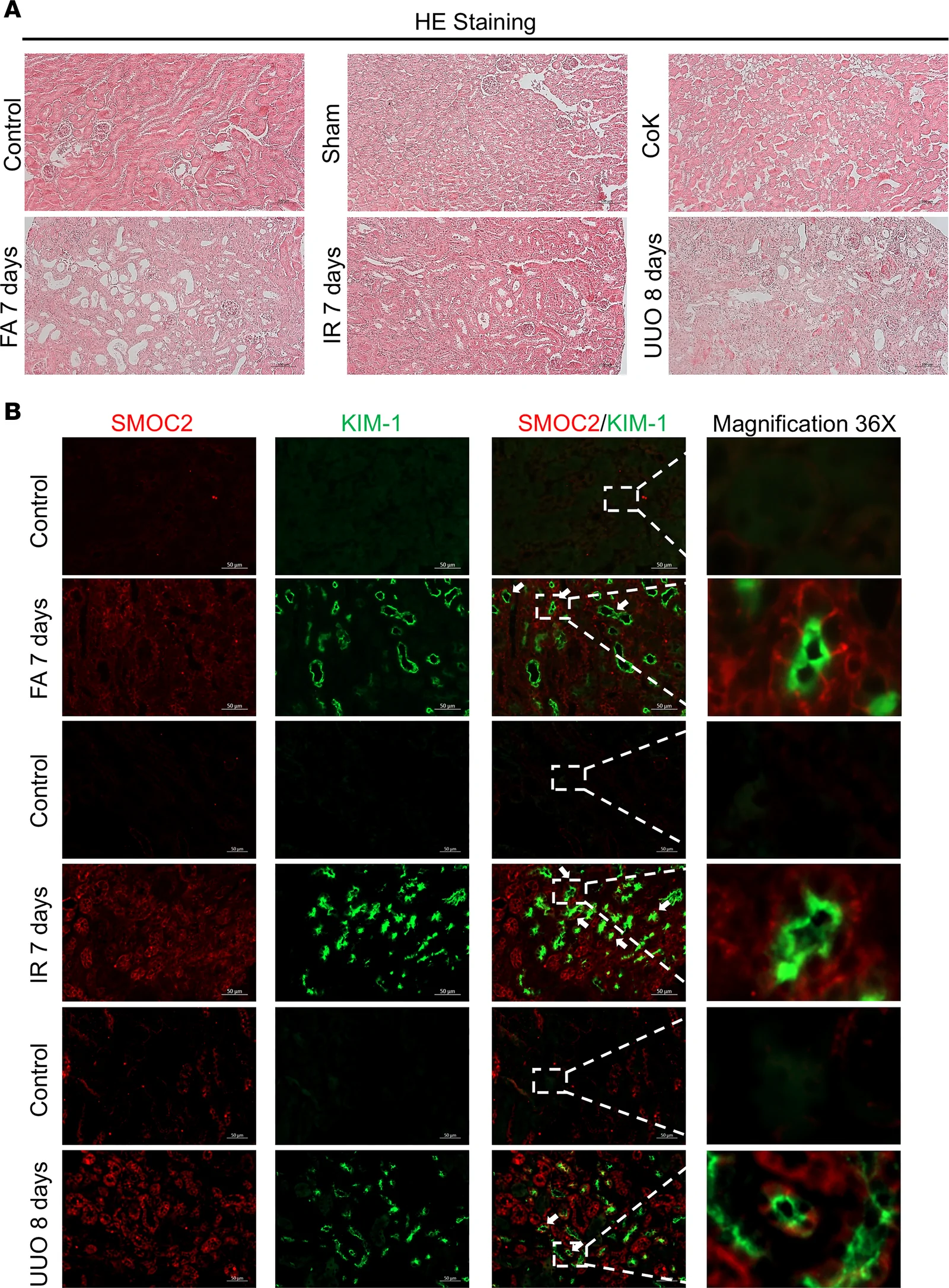
SMOC2 is localized at the tubular epithelium basement membrane and colocalized with tubular injury marker in different kidney regions. (**A**) H&E staining depicting the morphological structures of control and injured kidneys in folic acid (FA), ischemia-reperfusion (IR), and UUO models of kidney injury after 7 or 8 days. (**B**) Immunofluorescence showing the localization of SMOC2 and KIM-1 in folic acid (FA), ischemia-reperfusion (IR), and UUO models of kidney injury after 7 or 8 days. The arrows point to tubules positive for SMOC2 and KIM-1, while the dashed boxes indicate the magnified regions. The total original magnification is ×100; scale bar for HE staining: 100 μm; scale bar for immunofluorescence: 50 μm.

Previously, SMOC2 was found to localize primarily to the basement membrane of proximal and distal tubules (39). To determine whether SMOC2 localization is associated with TECs undergoing pEMT after injury, we first performed H&E staining to confirm injury in the mouse models (Figure 5A). We observed loss of brush borders and dilated tubules in the FA and UUO injury models, with less damage in the ischemia-reperfusion model, suggesting recovery in the latter (Figure 5A). We then performed immunofluorescence staining for SMOC2 and the proximal tubule injury marker KIM-1 in the UUO, FA, and IR models (Figure 5B). In all 3 models, both SMOC2 and KIM-1 were markedly increased in injured mice, with SMOC2 localized to the basement membrane of the kidney tubules and KIM-1 exhibiting an apical distribution (Figure 5B). Notably, several kidney tubules were double-positive for SMOC2 and KIM-1 (Figure 5B), particularly in the FA and IR models.

**Figure 6:**
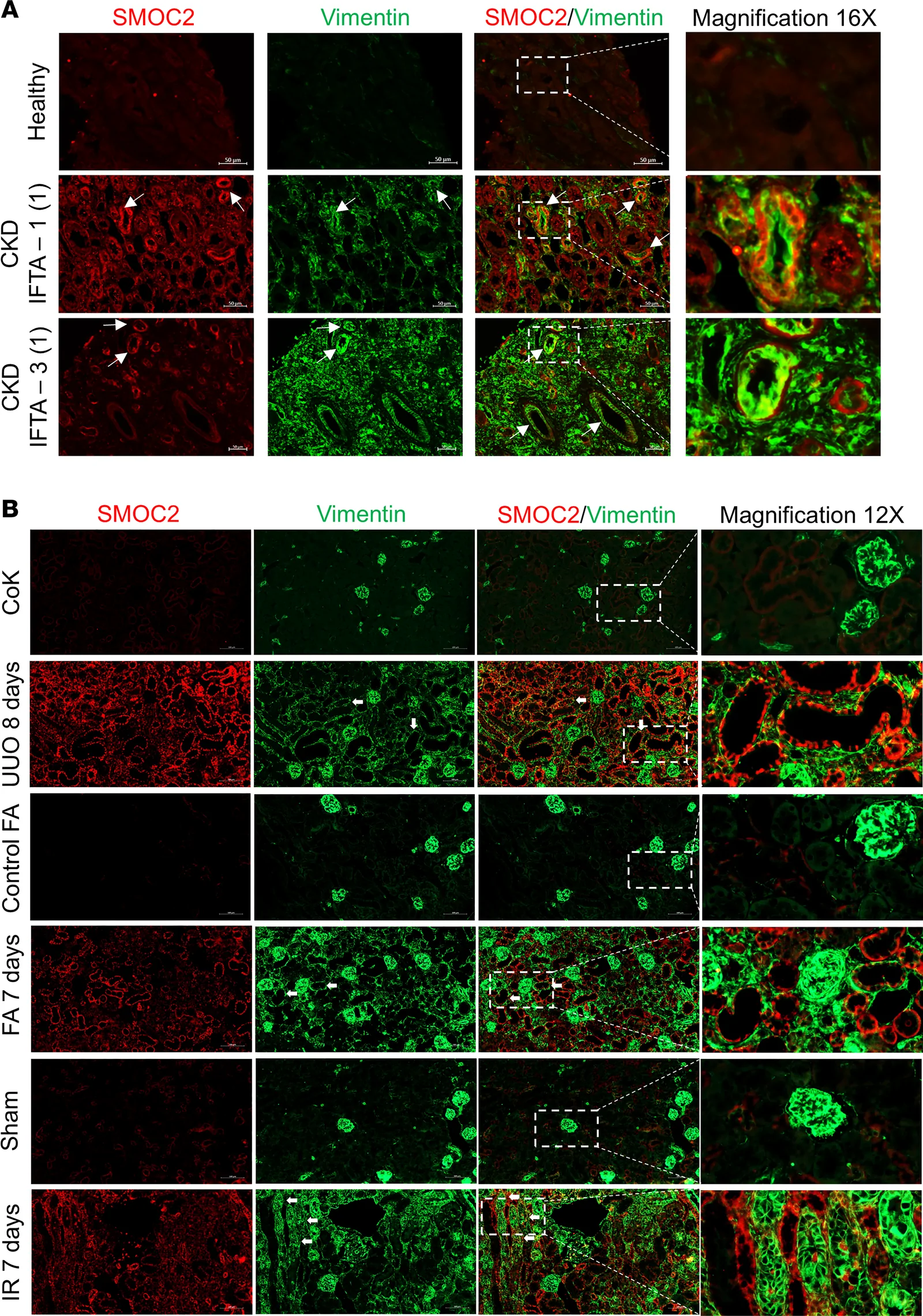
SMOC2 localization is associated with tubular cells undergoing pEMT in patients with CKD and murine models of kidney injury. (**A**) Immunofluorescence showing the localization of SMOC2 and vimentin in patients with CKD with a score of 1 or 3 for interstitial fibrosis and tubular atrophy (IFTA). (**B**) Immunofluorescence showing the localization of SMOC2 and vimentin in folic acid (FA), ischemia-reperfusion (IR), and UUO models of kidney injury after 7 or 8 days. The arrows point to tubules positive for SMOC2 and vimentin, while the dashed boxes indicate the magnified regions. Images were taken using a 10× objective. The total original magnification is ×100; scale bar for **A**: 50 μm; scale bar for **B**: 100 μm.

We then performed immunofluorescence for SMOC2 and the mesenchymal marker vimentin in patients with CKD and mouse kidney injury models. Kidneys of patients with CKD (IFTA scores 1 and 3) showed increased vimentin and SMOC2 levels compared with healthy kidneys, with SMOC2 localized in vimentin-expressing tubules, linking it to pEMT in CKD (Figure 6A and Supplemental Figure 2A). In all murine models, SMOC2 and vimentin increased after injury (Figure 6B). Vimentin was present in glomeruli in healthy and injured kidneys, with more interstitial expression after injury (Figure 6B). In the FA and UUO models, few tubules were positive for both markers, but ischemia-reperfusion showed more pronounced tubular staining for vimentin and SMOC2, indicating model-specific differences (Figure 6B). Immunofluorescence after 2 days of UUO confirmed similar patterns, with high expression in the glomeruli and interstitium but minimal in the tubules (Supplemental Figure 2B). Therefore, tubular vimentin expression showed a model-specific distribution rather than a temporal regulation. These results revealed that SMOC2 localization correlates with persistent tubular injury and pEMT, and that vimentin distribution varies by injury type.

### SMOC2 induces pEMT properties in proximal TECs.

**Figure 7:**
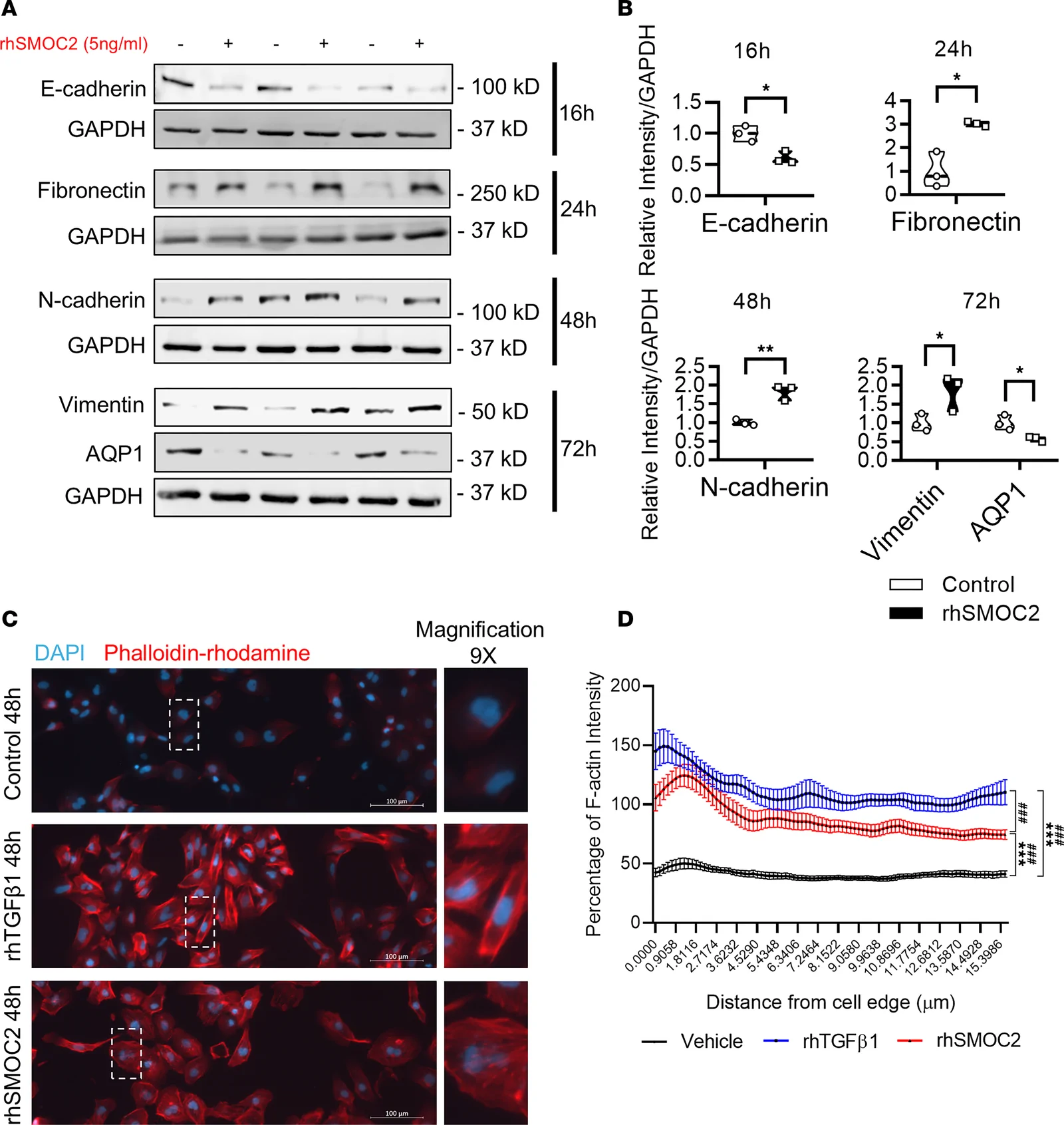
Recombinant SMOC2 stimulates the pEMT properties of proximal tubular epithelial cells. (**A**) Western blots showing the protein levels of E-cadherin, fibronectin, N-cadherin, vimentin, and aquaporin 1 (AQP1) in HK-2 cells stimulated with a recombinant human SMOC2 (rhSMOC2) protein at different timepoints. (**B**) Violin plots showing the quantification of the Western blots in **A**. (**C**) Phalloidin staining depicting the morphology and actin filament structures of HK-2 cells stimulated with rhSMOC2 or recombinant human TGF-β1 (rhTGF-β1) for 48 hours. The dashed boxes represent the magnified areas. Images were taken using a 10× objective. The total original magnification is ×100; scale bar for **C**: 100 μm. (**D**) Graph showing the actin distribution in control, rhSMOC2- or rhTGF-β1–treated HK-2 cells starting from a cell edge. Unpaired 2-tailed Student’s *t* test with Welch’s correction (**B**), 2-way ANOVA (**D**). ***P < 0.001 for interaction, ^###^*P* < 0.001 for column factor; *n* = 10 per condition. For Western blot analyses, *P < 0.05, **P < 0.01; *n* = 3 per condition.

**Figure 8:**
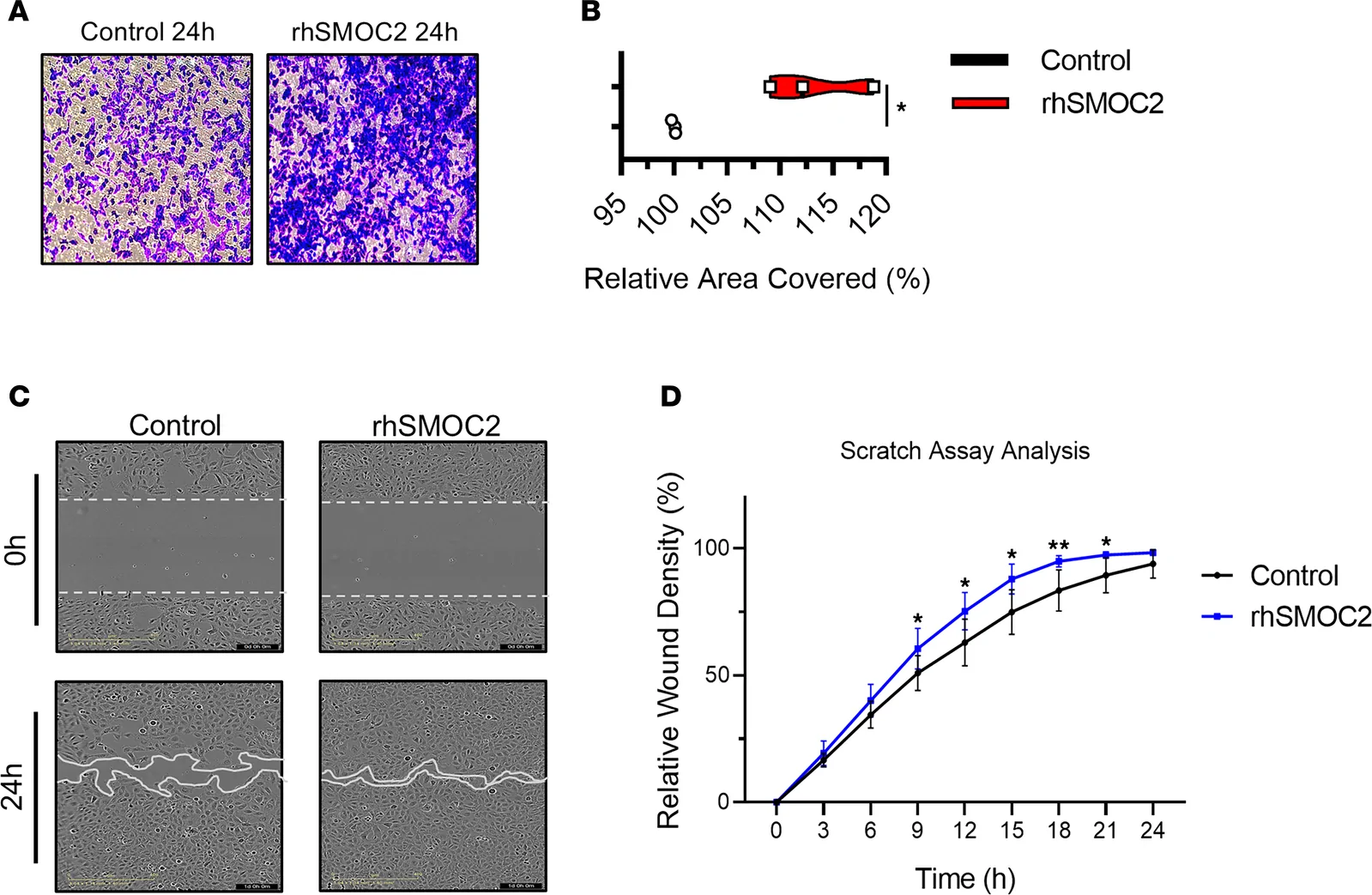
Recombinant SMOC2 increases the motility of proximal tubular epithelial cells. (**A** and **B**) Transwell assay and the corresponding quantification showing the chemotaxis of HK-2 cells stimulated with rhSMOC2 after 24 hours compared with nonstimulated (control) cells. The total original magnification for **A** and **C** is ×100; scale bar for **C**: 400 μm. (**C**) Scratch assay indicating the migration of HK-2 cells stimulated with rhSMOC2 after 24 hours compared with control cells. (**D**) Analysis of the scratch assay in **C** showing the relative wound density of rhSMOC2-stimulated HK-2 cells compared with the corresponding controls. Unpaired 2-tailed Student’s *t* test with Welch’s correction. *P < 0.05, **P < 0.01; *n* = 3 for each experiment.

Our findings in mouse tissues and patients with CKD linking SMOC2 to pEMT prompted us to test if SMOC2 induces pEMT in TECs. We stimulated HK-2 cells with recombinant human SMOC2 (rhSMOC2) at levels similar to those in the serum of patients with CKD (37). After stimulation, HK-2 cells showed an early decrease in E-cadherin and a gradual increase in fibronectin, N-cadherin, and vimentin (Figure 7, A and B), confirming pEMT as a dynamic process (43). SMOC2 modulates mesenchymal markers over time, with sustained vimentin and reduced N-cadherin during pEMT development (Supplemental Figure 3, A–E). Stimulated cells also exhibited lower AQP1, a proximal tubule water transporter (Figure 7, A and B). To observe morphological changes, we performed phalloidin staining, using recombinant human TGF-β1 (rhTGF-β1) as a positive control. After 48 hours, rhTGF-β1–treated HK-2 cells exhibited pEMT features, including increased actin filament formation and an elongated shape. Similarly, rhSMOC2-treated cells appeared enlarged with actin reorganized into stress fibers (Figure 7, C and D). We also evaluated chemotaxis and migration of rhSMOC2-stimulated HK-2 cells using transwell and scratch assays, respectively (Figure 8, A–D). Both confirmed that SMOC2 enhances TEC motility (Figure 8, A–D).

**Figure 9:**
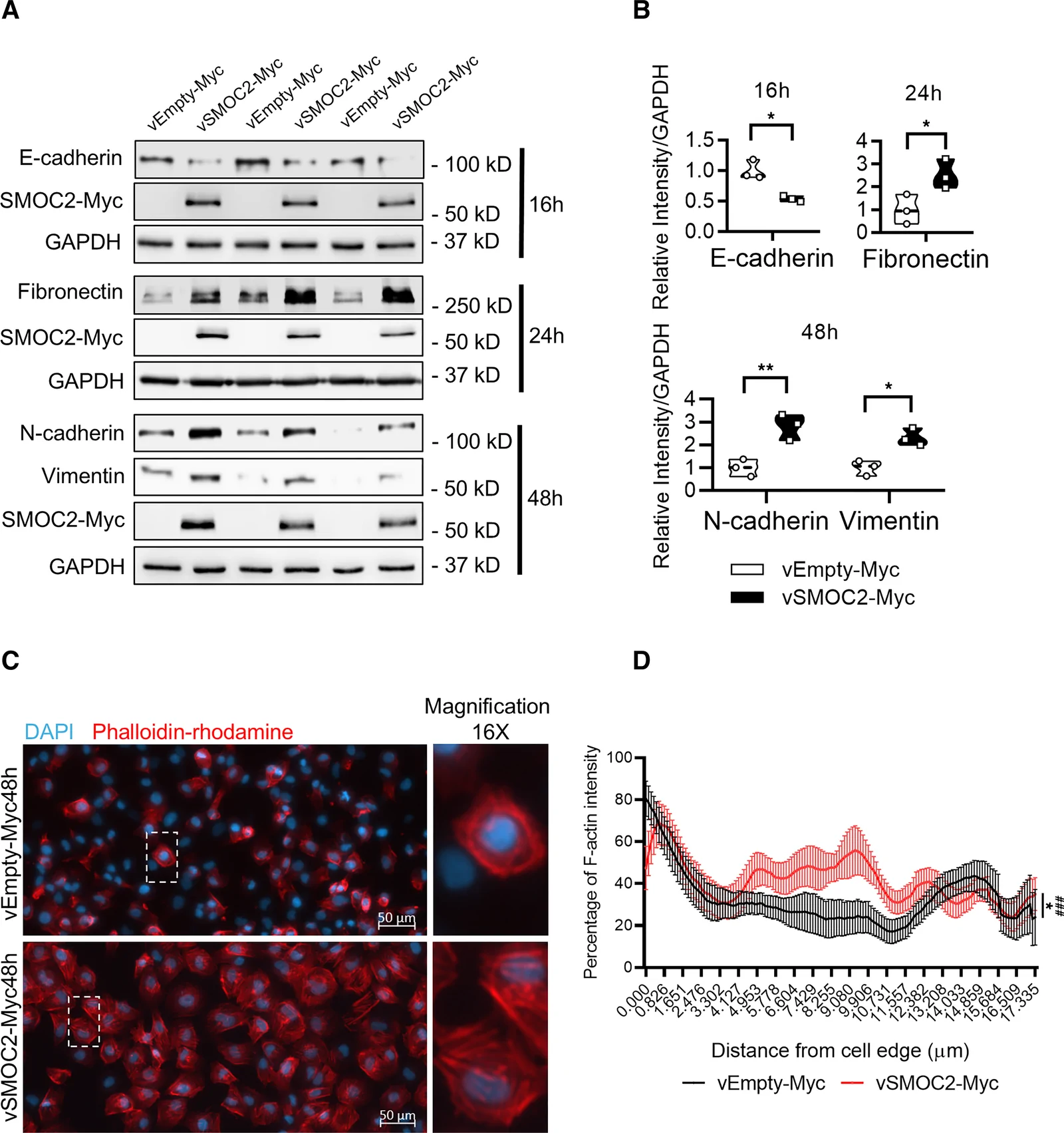
Ectopic expression of SMOC2 induces the pEMT properties of proximal tubular epithelial cells. (**A**) Western blots showing the protein levels of E-cadherin, fibronectin, N-cadherin, and vimentin in HK-2 cells ectopically expressing a SMOC2-Myc vector (vSMOC2-Myc) or the corresponding control vector with only the Myc-tag (vEmpty-Myc) at different time points. (**B**) Violin plots showing the quantification of the Western blots in **A**. (**C**) Phalloidin staining indicating the morphology and actin filament structures of HK-2 cells ectopically expressing vSMOC2-Myc or vEmpty-Myc for 48 hours. The dashed boxes represent the magnified regions. Images were taken using a 10× objective. The total original magnification is ×100; scale bar: 50 μm. (**D**) Graph showing the actin distribution in HK-2 cells ectopically expressing a Myc-tag (vEmpty-Myc) or a SMOC2-Myc (vSMOC2-Myc) vector. Unpaired 2-tailed Student’s *t* test with Welch’s correction (**B**), 2-way ANOVA (**D**). *P < 0.05 for interaction, ^###^*P* < 0.001 for column factor; *n* = 10 per condition. For Western blot analyses, *P < 0.05, **P < 0.01; *n* = 3 per condition.

**Figure 10:**
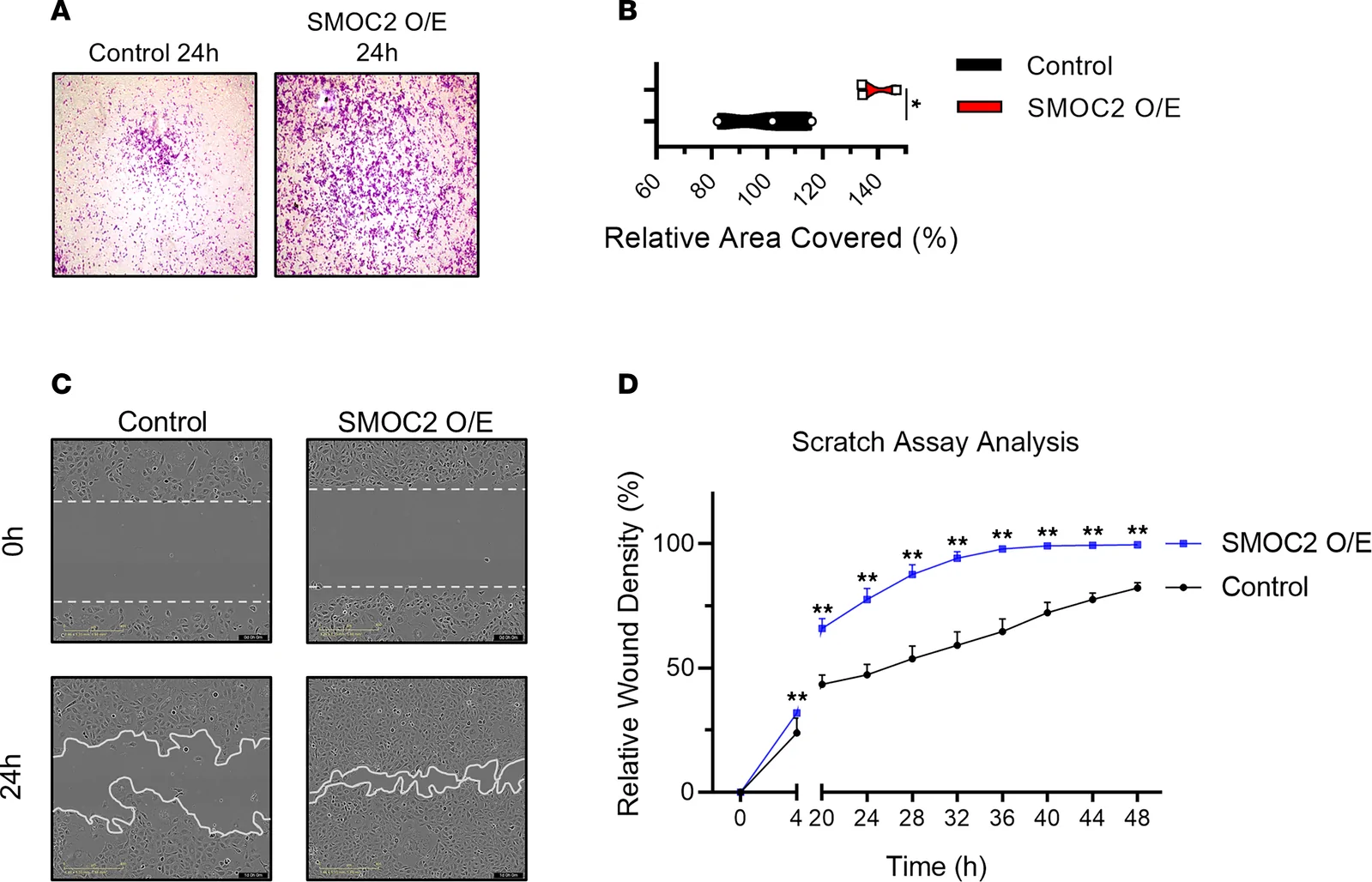
Ectopic expression of SMOC2 increases the motility of proximal tubular epithelial cells. (**A** and **B**) Transwell assay and the corresponding quantification (**B**) showing the chemotaxis of HK-2 cells transduced with a lentiviral vector containing SMOC2 (SMOC2 O/E) or a control lentiviral vector for 24 hours. (**C**) Scratch assay indicating the migration of transduced HK-2 cells ectopically expressing SMOC2 or the control lentiviral vector for 48 hours. Total magnification ×40 for **A** and ×100 for **C**. Scale bar for **C**: 400 μm. (**D**) Analysis of the scratch assay in **C** showing the relative wound density of HK-2 cells overexpressing SMOC2 compared with the cells expressing the control lentiviral vector. Unpaired 2-tailed Student’s *t* test with Welch’s correction. *P < 0.05, **P < 0.01; *n* = 3 per condition.

We next explored whether ectopic expression of SMOC2 in HK-2 cells would exert the same effects. Similar to rhSMOC2 treatment, ectopic expression of a vector encoding the full-length SMOC2 with a Myc tag (vSMOC2-Myc) decreased the protein levels of E-cadherin and increased fibronectin, N-cadherin, and vimentin when compared with the control vector (vEmpty-Myc) (Figure 9, A and B). Compared with the control HK-2 cells, which exhibit a round shape and actin filaments lining beneath the cell cortex, HK-2 cells ectopically expressing the full-length SMOC2 are enlarged and display actin filaments rearranged into stress fibers (Figure 9, C and D). Similar to rhSMOC2 treatment, stable overexpression of SMOC2 via lentiviral transduction increased chemotaxis and migration of HK-2 cells (Figure 10, A–D).

### SMOC2 achieves ECM binding and affects TECs through its calcium-binding domain.

SMOC2 is a modular protein with 5 domains of unknown function in kidney TECs.

**Figure 11:**
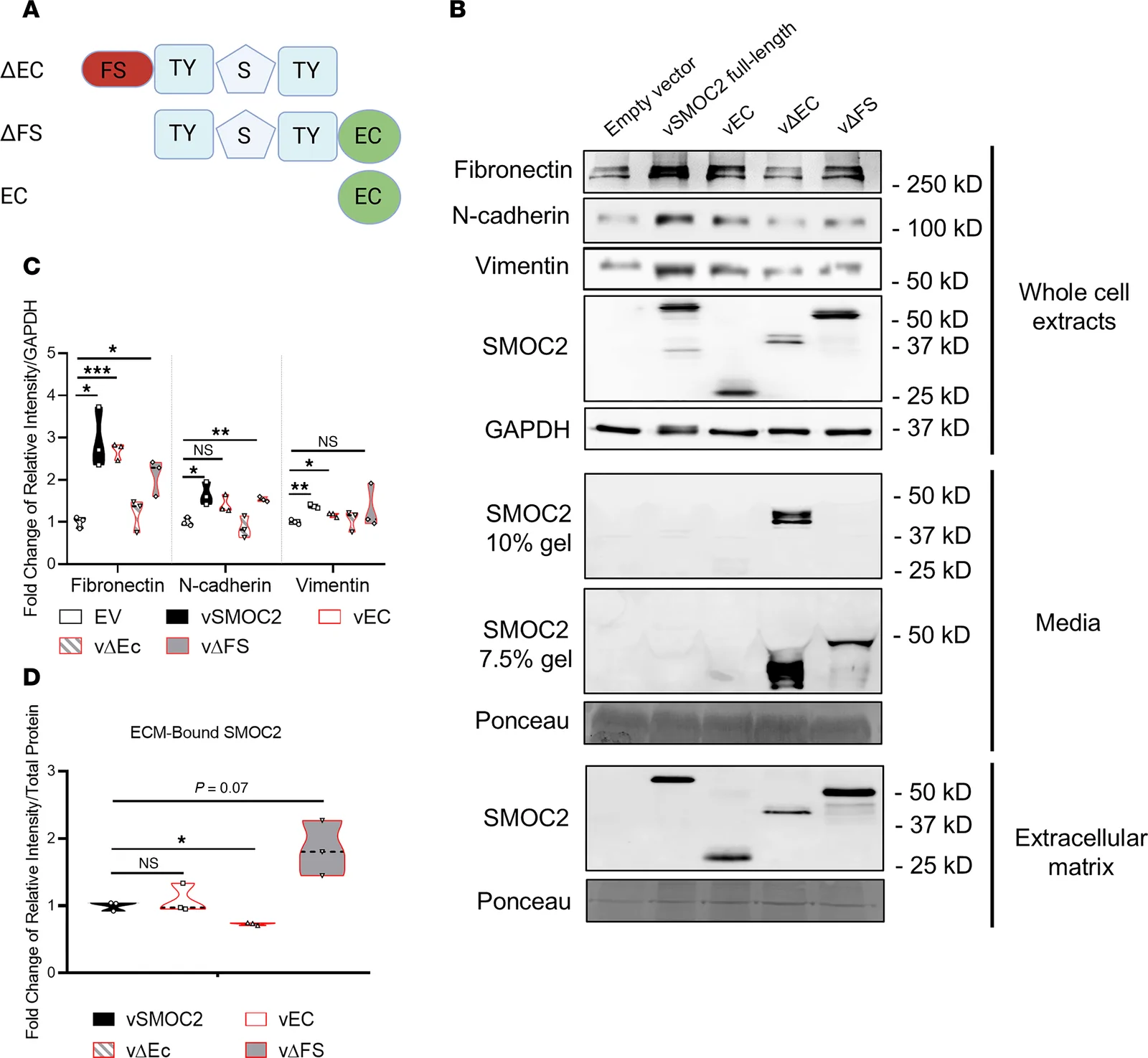
The extracellular calcium-binding domain of SMOC2 mediates its binding to the ECM to trigger the mesenchymal properties of tubular epithelial cells. (**A**) Schematic representation of the vector constructs used in the study. FS, follistatin-like domain; TY, thyroglobulin type I domains; S, SMOC-specific domain; EC, extracellular calcium-binding domain. (**B**) Western blots showing the protein levels of fibronectin, N-cadherin, vimentin, and SMOC2 in HK-2 cells ectopically expressing a control vector (Empty vector), full-length SMOC2, or different SMOC2 truncated mutants for 48 hours. The level of full-length SMOC2 and truncated mutants was measured in whole-cell extracts, the cells’ media, and the extracellular matrix. (**C**) Violin plots showing the quantification of the mesenchymal markers from whole-cell lysates in **B**. (**D**) Violin plots showing the quantification of the levels of SMOC2 and each truncated mutant in the ECM produced by the transfected HK-2 cells in **B**. One-way ANOVA was used for statistical analyses. *P < 0.05, **P < 0.01, ***P < 0.001; *n* = 3 per condition.

**Figure 12:**
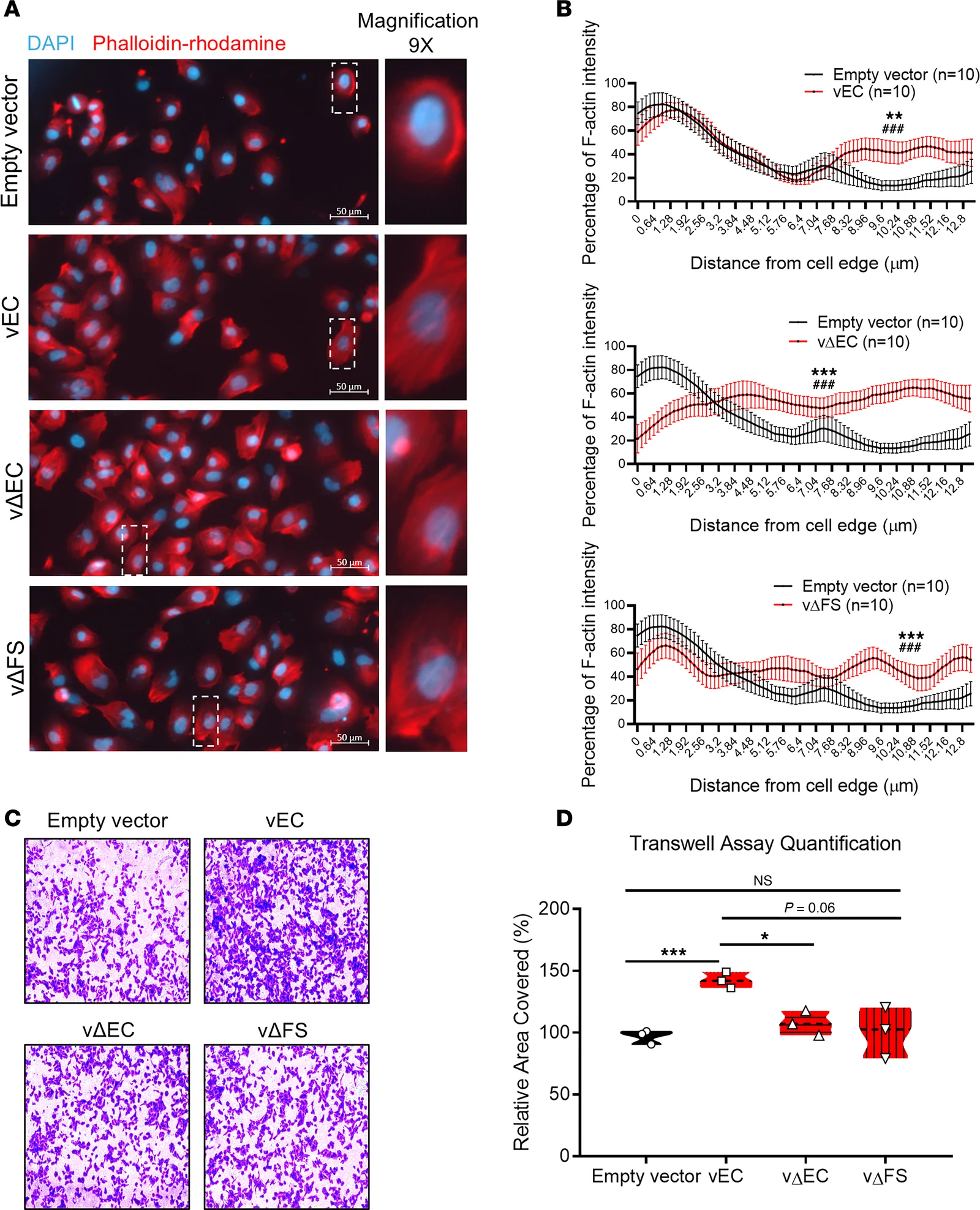
The extracellular calcium binding and the follistatin domains of SMOC2 contribute to cytoskeletal rearrangement and migration of tubular epithelial cells. (**A**) Phalloidin staining showing the morphology and actin filament structures of HK-2 cells ectopically expressing a control vector or different SMOC2 truncated mutants for 48 hours. The dashed boxes represent the magnified regions. Images were taken using a 10× objective. (**B**) Graphs showing the actin distribution in HK-2 cells expressing different SMOC2 truncated mutants compared with HK-2 cells expressing a control vector. A 2-way ANOVA was used for statistical analyses. **P < 0.01, ***P < 0.001 for interaction, ^###^*P* < 0.001 for column factor; *n* = 10 per condition. (**C**) Transwell assay showing the chemotaxis of HK-2 cells ectopically expressing a control vector or different SMOC2 truncated mutants for 24 hours. (**D**) Violin plots showing the quantification of the transwell assay in **C**. One-way ANOVA; *P < 0.05, ***P < 0.001, ns: not significant; *n* = 3 per condition. 25. The total original magnification for **A** and **C** is ×100; scale bar for **A**: 50 μm.

To identify which domain mediates effects in HK-2 cells, we used mutants lacking the extracellular calcium-binding domain (vΔEC, where v indicates vector and Δ indicates deletion), follistatin domain (vΔFS), or containing only the calcium-binding domain (vEC) (Figure 11A). The vEC and vΔFS mutants increased fibronectin, vimentin, and N-cadherin levels similarly to full-length SMOC2 in HK-2 cells; vΔEC had no effect (Figure 11, B and C). We then collected the media and the ECM from transfected cells to analyze the localization of each mutant, using 10% and 7.5% gels to detect small proteins and separate larger proteins from albumin. The analysis revealed that the ΔEC mutant was abundant in the media but low in the ECM, indicating the EC domain is necessary for matrix binding (Figure 11, B and D). The full-length protein and EC domain were mainly in the matrix, whereas ΔFS was in both the media and the matrix (Figure 11, B and D). Intriguingly, the actin cytoskeleton was affected by all constructs (Figure 12, A and B). The EC domain significantly enhanced chemotaxis in HK-2 cells, unlike the other mutants (Figure 12, C and D).

### SMOC2 promotes the activation of pEMT transcription factors.

**Figure 13:**
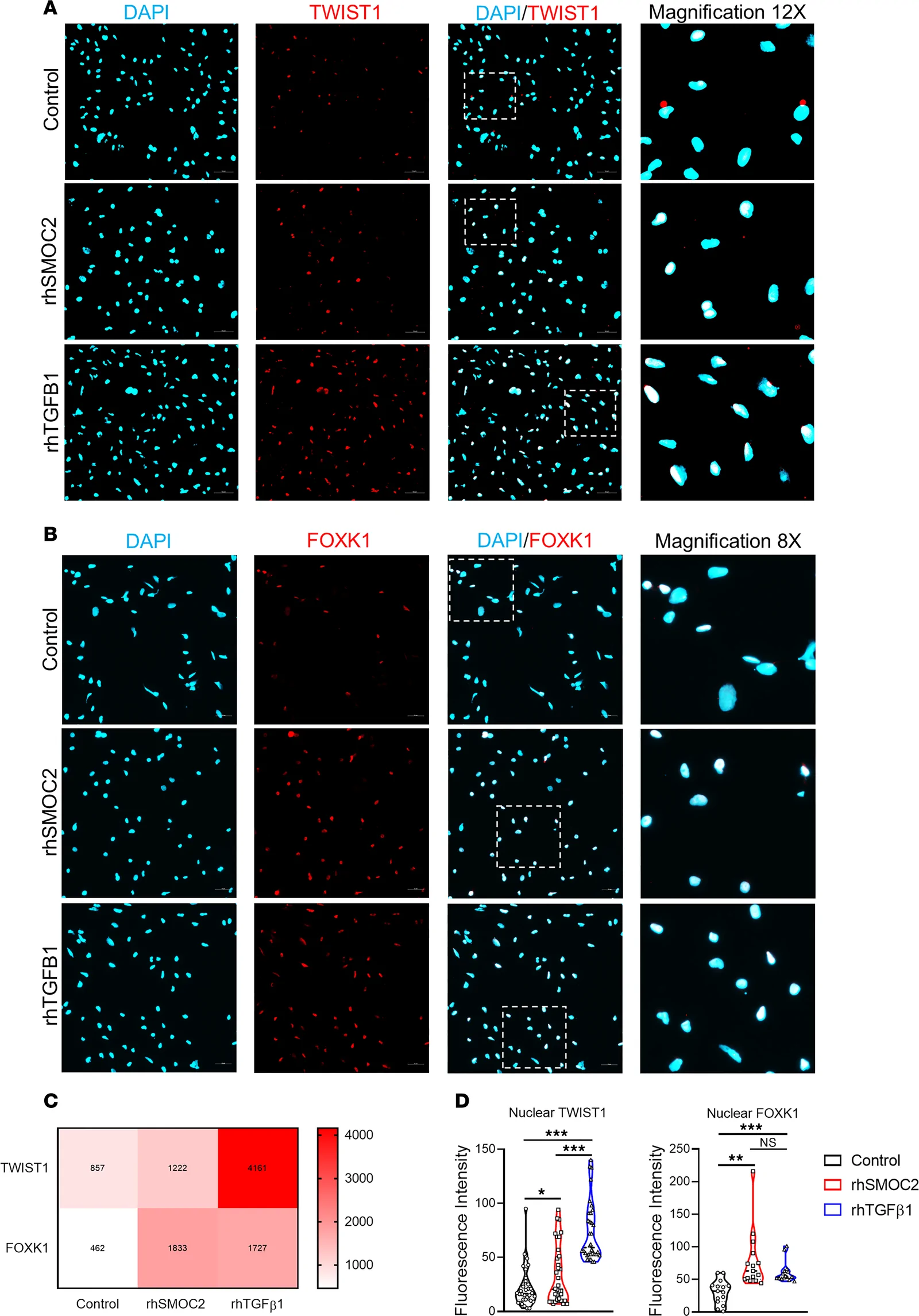
SMOC2 promotes the activation of pEMT transcription factors. (**A** and **B**) Immunofluorescence staining showing the expression and localization of TWIST1 (**A**) and FOXK1 (**B**) in unstimulated cells (control) and cells stimulated with rhSMOC2 or rhTGF-β1 for 48 hours. The dashed boxes represent the magnified areas. Images were taken using a 20× objective. (**C**) Heatmap showing the total level of TWIST1 and FOXK1 in control, rhSMOC2- or rhTGF-β1–treated cells. (**D**) Violin plots showing the nuclear signal for TWIST1 and FOXK1 in control, rhSMOC2- or rhTGF-β1–treated cells. One-way ANOVA; *P < 0.05, **P < 0.01 ***P < 0.001; *n* = 15–34. The total original magnification is ×100; scale bar for **A** and **B**: 50 μm.

Partial EMT is regulated by critical transcription factors, with SNAI1 and TWIST1 being the most studied (13, 16, 25, 26, 44, 48). To investigate SMOC2’s effect on pEMT transcription factors, we performed qPCR for *SNAI1* and *TWIST1* in HK-2 cells stimulated by rhSMOC2 or transfected to overexpress SMOC2. The expression of both was unaffected by SMOC2 (Supplemental Figure 3F). We then analyzed the levels and localization of pEMT transcription factors using immunofluorescence for TWIST1 and FOXK1. After rhSMOC2 treatment, HK-2 cells showed mild upregulation of TWIST1 but strong nuclear localization compared with controls (Figure 13A). FOXK1 was upregulated and localized to the nucleus, similar to the effects of rhTGF-β1 (Figure 13, B–D). These findings suggest that SMOC2 activates pEMT transcription factors without increasing their gene expression.

### SMOC2 stimulates the mesenchymal properties of TECs through integrin signaling.

**Figure 14:**
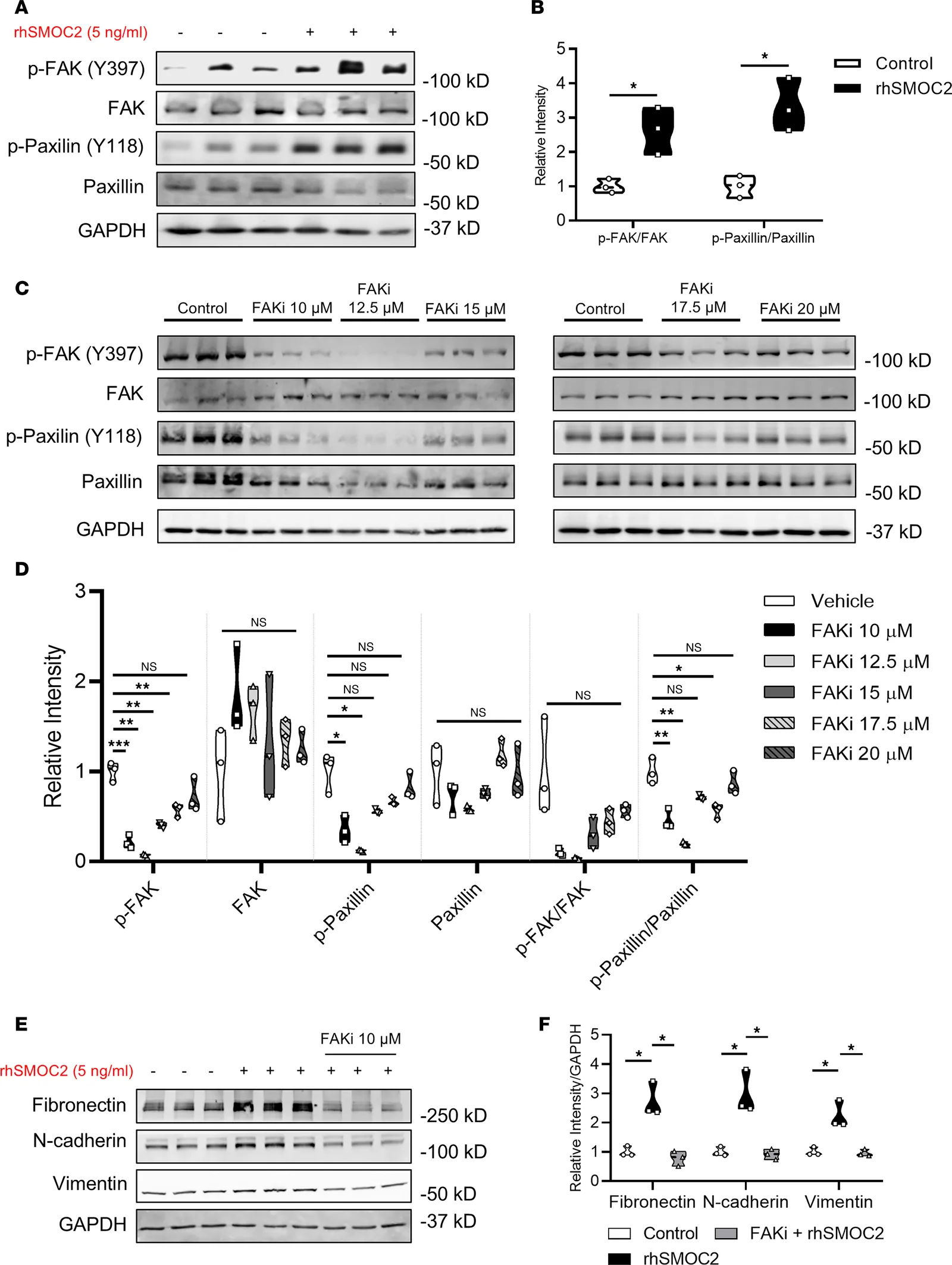
SMOC2 activates integrin signaling to stimulate the pEMT properties of tubular epithelial cells. (**A**) Western blots showing the protein levels of phosphorylated FAK (pFAK), phosphorylated paxillin (pPaxillin), and their total levels in rhSMOC2-treated HK-2 cells after 10 minutes. (**B**) Quantification of the Western blots in **A**. (**C**) Western blots showing pFAK, pPaxillin levels, and their total levels in HK-2 cells treated with a gradient of FAK inhibitor (FAKi) for 48 hours. (**D**) Quantification of the Western blots in **C**. (**E**) Western blots indicating the levels of mesenchymal markers fibronectin, N-cadherin, and vimentin in HK-2 cells stimulated with rhSMOC2 with or without FAKi treatment. (**F**) Quantification of the Western blots in **E**. Unpaired 2-tailed Student’s *t* test with Welch’s correction (**B**), 1-way ANOVA (**D** and **F**). *P < 0.05, **P < 0.01, ***P < 0.001; *n* = 3 for each experiment.

SMOC2 was previously found to interact with integrin β1 in muscle stem cells, keratinocytes, and kidney fibroblasts (39, 49, 50). In clear cell renal cell carcinoma (ccRCC) cells, SMOC2 also activates key components of the integrin signaling pathway, including focal adhesion kinase (FAK) and paxillin (33). To investigate whether SMOC2 activates integrin signaling in TECs, we stimulated HK-2 cells with rhSMOC2 for 10 minutes. SMOC2 increased FAK autophosphorylation on tyrosine 397 and downstream phosphorylation of paxillin on tyrosine 118 (Figure 14, A and B). We then inhibited FAK with a small-molecule inhibitor (FAKi). A concentration gradient showed that 10 μM and 12.5 μM effectively prevented phosphorylation of FAK and paxillin after 48 hours (Figure 14, C and D). We used 10 μM for subsequent experiments due to minimal toxicity. Preincubating HK-2 with 10 μM FAKi abrogated the upregulation of fibronectin, N-cadherin, and vimentin levels by rhSMOC2 (Figure 14, E and F). Since TGF-β1 is a key profibrotic factor, we tested if SMOC2 influences its pathway. While SMOC2 regulates TGF-β1 signaling via SMADs in pulmonary fibrosis (36), our results show it does not affect SMAD phosphorylation in tubular cells (Supplemental Figure 4, A and B). Cotreatment with SMOC2 and TGF-β1 further increased mesenchymal markers compared with TGF-β1 alone, indicating an additive effect in promoting pEMT (Supplemental Figure 4, C and D).

### Integrins β1 and β3 are important receptors for SMOC2 in TECs.

**Figure 15:**
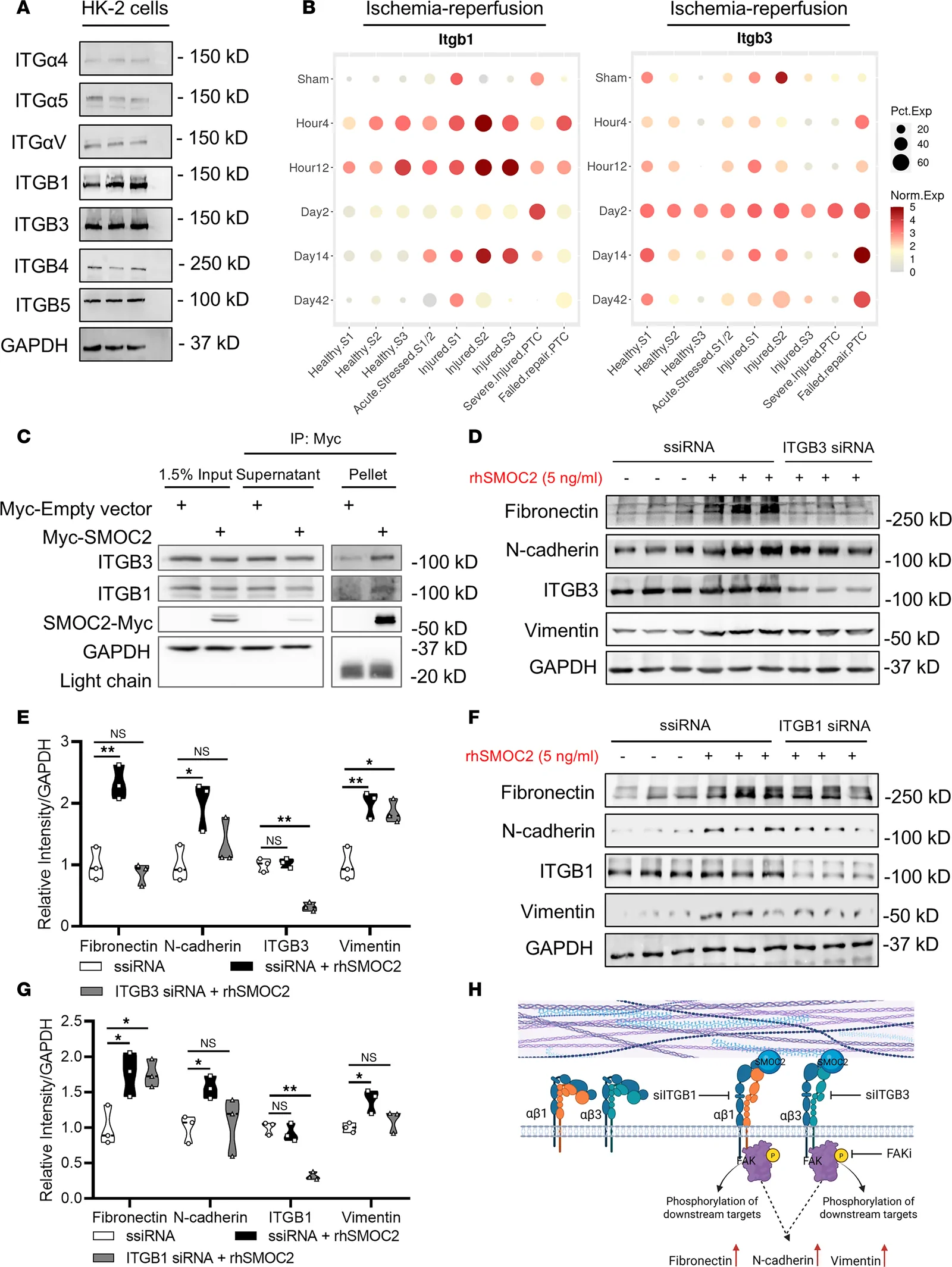
Integrins β1 and β3 are important ECM receptors mediating the effects of SMOC2 in tubular epithelial cells. (**A**) Western blots showing the expression of several integrins in HK-2 cells under normal conditions. (**B**) Single-cell RNA-seq data showing the gene expression of integrins β1 (Itgb1) and β3 (Itgb3) in proximal tubular cells (PTC) after ischemia-reperfusion injury. These images were generated by the Software from the Kidney Interactive Transcriptomics. (**C**) Immunoprecipitation indicating the binding of SMOC2 to integrins β1 and β3 in HK-2 cells. (**D** and **F**) Western blots showing the expression of mesenchymal markers in HK-2 cells transfected with a scrambled siRNA (ssiRNA), or a siRNA targeting integrin β3 (ITGB3 siRNA) (**D**) or integrin β1 (ITGB1 siRNA) (**F**). The transfected cells were stimulated with rhSMOC2 or left nonstimulated for 48 hours. (**E** and **G**) Quantification of the Western blots in (**D** and **F**), respectively. (**H**) Schematic representation of the mechanism for SMOC2-regulated mesenchymal proteins in tubular epithelial cells. One-way ANOVA; *P < 0.05, **P < 0.01; *n* = 3 for each experiment.

To further investigate the integrins involved in FAK activation after rhSMOC2 treatment, we first examined their expression in HK-2 cells and found that they expressed several α and β integrins at both protein and mRNA levels (Figure 15A and Supplemental Figure 5A). Analysis of existing single-cell RNA-seq data shows that integrins β1 and β3 were upregulated in TECs in UUO and ischemia-reperfusion (Figure 15B and Supplemental Figure 5, B–E) (17, 51). Integrin β3 is notably upregulated in failed-repair proximal tubular cells during chronic ischemia-reperfusion, suggesting a role in maladaptive repair (Figure 15B). To test if SMOC2 interacts with these integrins, we performed immunoprecipitation in HK-2 cells expressing SMOC2-Myc. Using Myc beads, we found that SMOC2 interacts with integrins β1 and β3 (Figure 15C). We then studied these integrins’ roles in mesenchymal marker induction by rhSMOC2. Silencing integrin β3 with siRNA prevented fibronectin upregulation after rhSMOC2, with no effect on vimentin (Figure 15, D and E). Silencing integrin β1 did not affect fibronectin but affected vimentin (Figure 15, F and G). Both integrins modestly affected N-cadherin (Figure 15, D–H). To explore their signaling, we silenced integrins β1 and β3 24 hours before stimulating HK-2 cells with rhSMOC2 for 30 minutes. Silencing integrin β3 decreased phosphorylation of FAK and paxillin; integrin β1 silencing had a modest effect (Supplemental Figure 6, A and B). We then used Cilengitide, which has higher affinity for integrin β3 and affects integrin β1 at higher doses (52, 53). At low doses (1–15 nM), cilengitide modestly reduced FAK phosphorylation after rhSMOC2, suggesting β3-mediated FAK phosphorylation (Supplemental Figure 6, C and D). These findings show that integrins β1 and β3 are SMOC2 receptors in TECs and cooperate to induce mesenchymal markers, mainly via β3.

### SMOC2 regulates integrin signaling and pEMT in injured kidneys following UUO.

**Figure 16:**
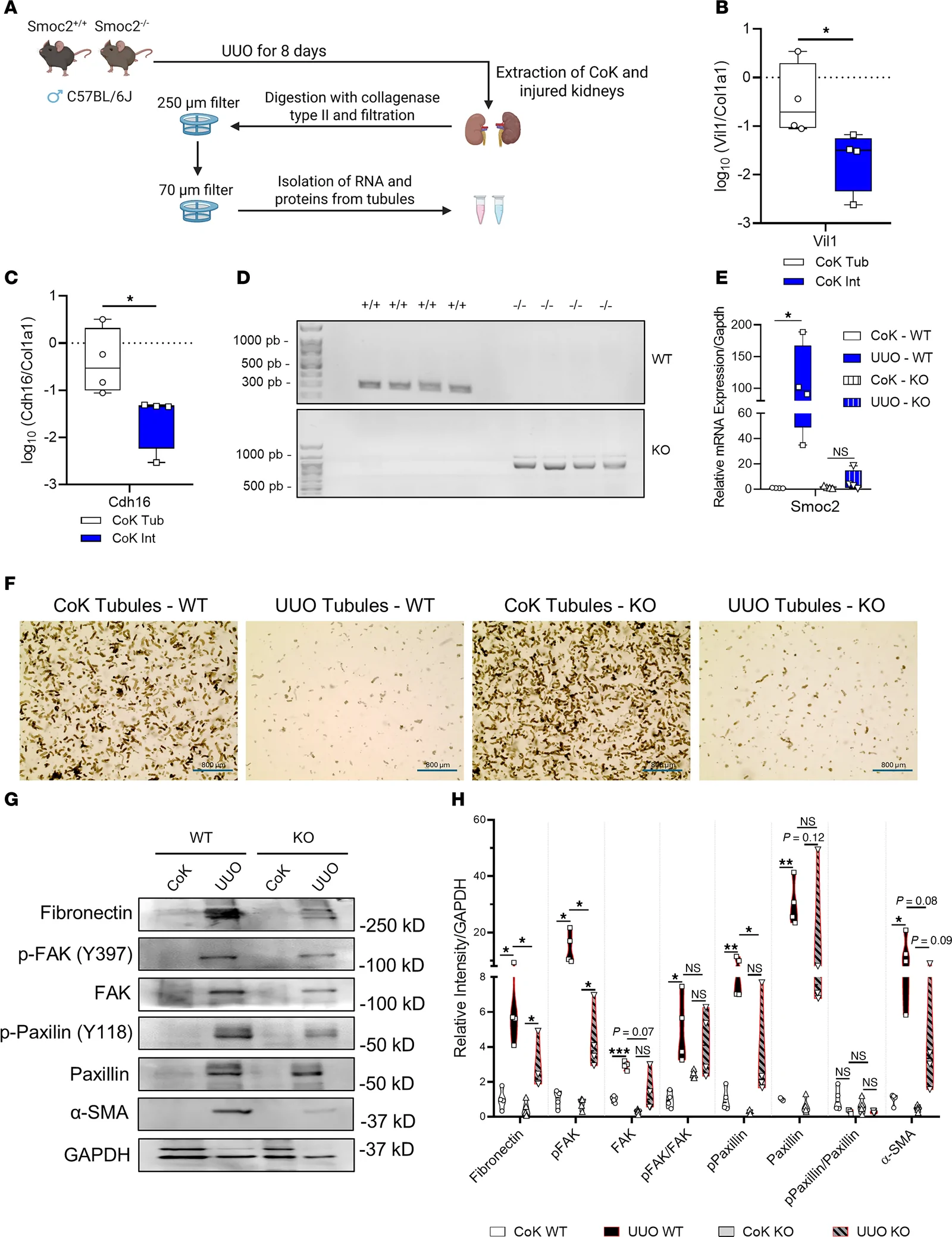
SMOC2 regulates integrin signaling and pEMT in mice undergoing UUO. (**A**) Diagram depicting the study design for the analyses of tubules after UUO. Male C57BL/6J mice, WT and Smoc2-KO, were subjected to UUO for 8 days. Tubules were isolated from control and injured kidneys for qPCR and Western blot analyses. (**B** and **C**) Box plots showing the enrichment for the ratio of epithelial genes *Vil1* (**B**) and *chd16* (**C**) over collagen I (*Col1a1*) for the tubules (Tub) and the interstitium (Int) following the extraction of tubules. Gene expression was measured by qPCR, and statistical tests were performed after log transformation. (**D**) Genotyping confirming the presence of the *Smoc2* gene in WT mice (300 pb) and the disrupted gene in Smoc2-KO mice (900 pb). (**E**) Box plots showing the gene expression of *Smoc2* measured by qPCR in contralateral and UUO tubules from WT and Smoc2-KO mice. Data are presented as mean ± SD. (**F**) Microscopic images of tubules isolated from contralateral and UUO kidneys from WT and Smoc2-KO mice. Images were taken using a 10× objective. (**G**) Western blots showing the total levels of FAK and paxillin, and the levels of autophosphorylated FAK (p-FAK, Y397), phosphorylated paxillin (p-paxillin, Y118), fibronectin, and α-SMA from contralateral and UUO tubules of WT and Smoc2-KO mice. (**H**) Violin plots showing the quantification of the Western blots in **G**. Unpaired 2-tailed Student’s *t* test with Welch’s correction (**B** and **C**), 1-way ANOVA (**E** and **H**). *P < 0.05, **P < 0.01, ***P < 0.001; *n* = 4 per condition. Scale bar: 800 μm.

Our in vivo findings linking SMOC2 expression and pEMT, along with in vitro pathway insights, led us to hypothesize that SMOC2 regulates pEMT in injured kidneys. To evaluate this, we isolated tubules from WT and Smoc2-KO mice after UUO (Figure 16A). qPCR showed that tubules are enriched for *Vil1* and *Cdh16* relative to *Col1a1*, confirming successful isolation (Figure 16, B and C). Genotyping confirmed the absence of *Smoc2*, and qPCR showed unchanged *Smoc2* expression in KO tubules after UUO (Figure 16, D and E). Tubule morphology was not different between WT and Smoc2-KO mice (Figure 16F). Western blots indicated decreased phosphorylation of FAK and paxillin in injured kidneys of Smoc2-KO mice versus WT littermates (Figure 16, G and H). Total FAK increased in injured kidneys and decreased in KO mice compared with WT, while paxillin was unchanged (Figure 16, G and H). Partial EMT markers fibronectin and α-SMA decreased in injured KO mice compared with WT, linking integrin signaling to pEMT (Figure 16, G and H).

### Kidney TECs stimulated by rhSMOC2 crosstalk with fibroblasts to promote fibrosis.

**Figure 17:**
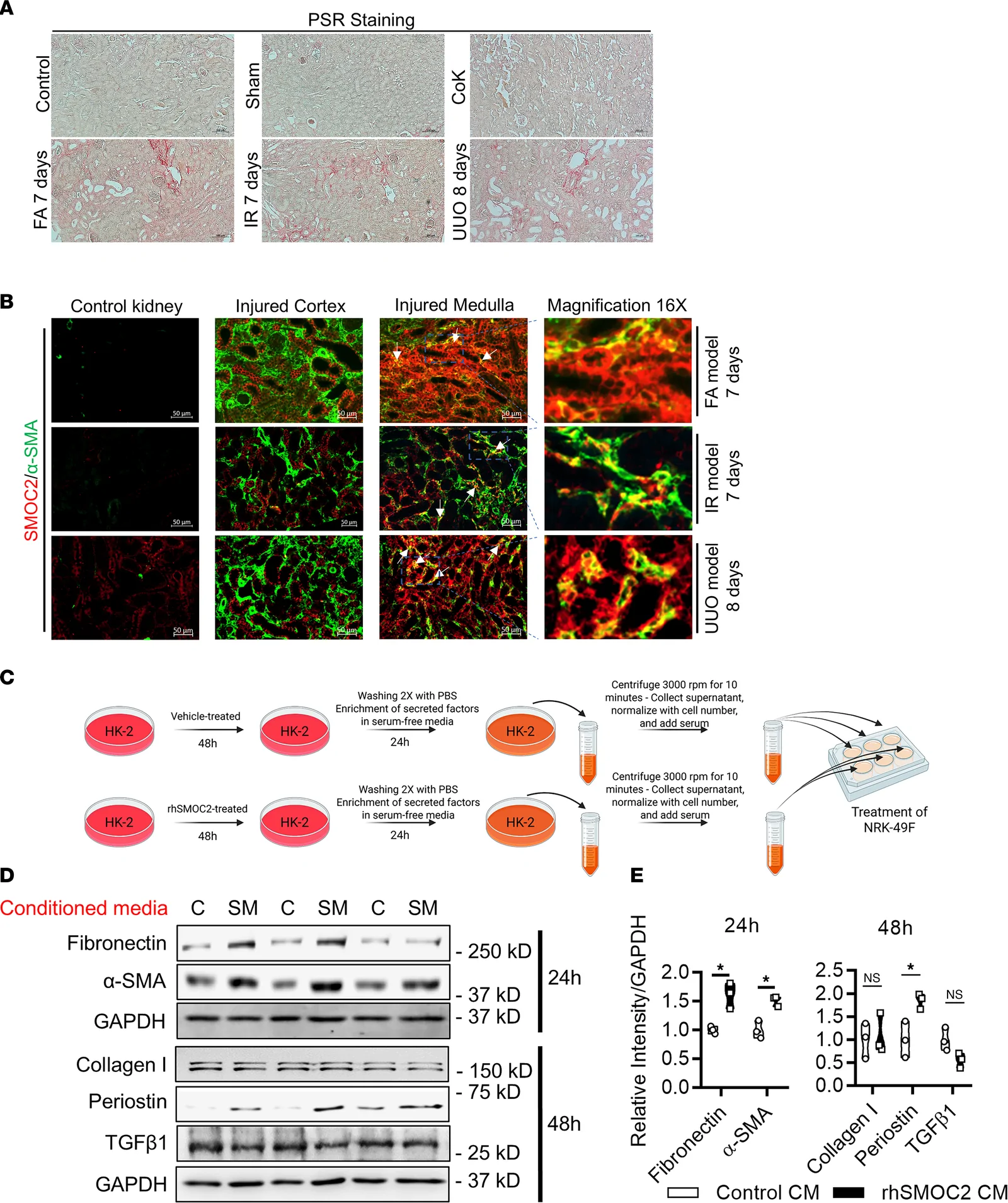
SMOC2 promotes an epithelial-fibroblast crosstalk during fibrogenesis. (**A**) Picrosirius red (PSR) staining depicting the burden of fibrosis after 7 days of folic acid (FA), ischemia-reperfusion (IR), and 8 days of UUO in mice kidneys. (**B**) Immunofluorescence indicating the localization of SMOC2 and the myofibroblast marker α-SMA in the cortex and medulla of injured mice in all 3 models of kidney injury. The arrows indicate kidney regions where SMOC2 and α-SMA are colocalized; the dashed boxes indicate the magnified areas. Images were taken using a 10× objective. (**C**) Diagram depicting the experimental design of the conditioned media treatment. HK-2 cells were stimulated with rhSMOC2 or vehicle-treated for 48 hours. The secreted factors were enriched for 24 hours, and NRK-49F cells were treated with the conditioned media for 24 and 48 hours. (**D**) Western blots showing the protein levels of fibronectin, α-SMA, collagen I, periostin, and the activated form of TGF-β1 in NRK-49F stimulated with conditioned media from vehicle-treated (**C** for control) or rhSMOC2-treated (SM for SMOC2) HK-2 cells for the indicated times. (**E**) Quantification of the Western blots in **D** for NRK-49F cells stimulated with the conditioned media (CM) from rhSMOC2-treated or control HK-2 cells. Unpaired 2-tailed Student’s *t* test with Welch’s correction. *P < 0.05, **P < 0.01; *n* = 3 per condition. The total original magnification is ×100; scale bar for **A**: 100 µm; scale bar for **B**: 50 μm.

Previous studies on genetic mouse models where pEMT is prevented show less fibroblast activation and fibrosis after kidney injury (13, 16). We assessed fibrosis in our kidney injury models with Picrosirius red (PSR) staining, detecting positive staining in FA and IR models after 7 days and in the UUO model after 8 days, confirming fibrosis (Figure 17A). Earlier research indicates SMOC2 interacts with fibroblasts to promote activation (38, 39). Our immunofluorescence for SMOC2 and α-SMA showed 2 patterns: in the cortex, SMOC2 localized to the tubular basement membrane without overlapping α-SMA, while in the medulla, SMOC2 partly overlapped with α-SMA, indicating interaction with fibroblasts (Figure 17B). We then hypothesized that SMOC2 activates fibroblasts paracrinely, based on prior pEMT findings suggesting crosstalk between TECs and fibroblasts to promote fibrosis (13, 16). Using conditioned media from rhSMOC2-stimulated HK-2 cells, we stimulated NRK-49F cells (Figure 17C). After 24 hours, fibroblasts showed increased levels of fibronectin and α-SMA, early activation markers, in rhSMOC2-stimulated conditioned media (Figure 17, D and E). At 48 hours, periostin was elevated, but collagen I was unchanged (Figure 17, D and E). Since TGF-β1 is essential for collagen I production (54), we evaluated activated TGF-β1 levels, which remained unchanged, explaining the lack of collagen I (Figure 17, D and E). These findings reveal that rhSMOC2-stimulated TECs undergoing pEMT activate nearby fibroblasts via paracrine signaling.

## Discussion

TECs are the most abundant cell type in the renal parenchyma and are highly susceptible to injury, leading to the development of CKD (10, 11, 55). After injury, TECs also respond to proinflammatory and reparative factors to foster recovery, but prolonged activity becomes maladaptive, leading to kidney fibrosis and organ failure (11, 56, 57). SMOC2 is a secreted protein that promotes fibrosis in ischemic, obstructive, and toxicant models of kidney injury through its profibrotic effects on fibroblasts (38, 39). This study emphasizes the role of TECs as a key cell type affected by SMOC2, which is essential for understanding how SMOC2 influences kidney fibrosis. We demonstrate that sustained SMOC2 expression correlates with maladaptive TEC repair after kidney injury and colocalizes with KIM-1 in 3 mouse models. The absence of colocalization in some kidney regions suggests that SMOC2 may be restricted to a specific cellular state or injury phase that has progressed to a maladaptive repair phenotype. Consequently, SMOC2 does not label all injured tubules but rather marks a distinct subset of epithelial cells undergoing pEMT. In patients with CKD and mouse models, SMOC2 was localized in TECs expressing vimentin, a mesenchymal marker. Our data also show that SMOC2 induces pEMT in TECs in vitro and modulates pEMT in injured kidneys after UUO. Mechanistically, SMOC2 interacts via its EC-binding domain with the ECM and influences the mesenchymal features of TECs. Furthermore, SMOC2 activates β1 and β3 integrins, with each receptor contributing to specific mesenchymal characteristics. Similar to MCPs such as osteopontin (58–60), periostin (61, 62), and tenascin-C (41, 63), SMOC2 is induced following injury and contributes to tissue remodeling and fibrogenesis. However, our findings highlight a potentially distinct role for SMOC2 in directly regulating epithelial cell plasticity, as we show that SMOC2 promotes a pEMT program in TECs. While previous work, including our own, has demonstrated that SMOC2 deficiency attenuates fibrosis, the present study extends these observations by identifying a cell-intrinsic mechanism linking SMOC2 to epithelial reprogramming during CKD.

pEMT is a process in which TECs downregulate epithelial genes and express mesenchymal markers while remaining attached to their basement membrane (10, 13, 16, 25, 27, 43). Moreover, they promote fibroblast activation into myofibroblasts by secreting profibrotic factors (13, 16, 26, 27, 43). This process is regulated by critical transcription factors such as SNAI1 and TWIST1 (13, 16, 27). Recently, FOXK1 has been shown to be crucial in regulating maladaptive repair of TECs in CKD by activating glycolysis and pEMT (42, 43). This study shows that Smoc2-transgenic mice exhibit increased *Foxk1* and mesenchymal gene expression after UUO, reduced epithelial transporters, and enhanced profibrotic pathways. SMOC2 promotes the nuclear translocation of FOXK1 and TWIST1 in TECs in vitro, likely via posttranslational modifications, as their expression is unaffected. It promotes crosstalk between TECs and fibroblasts and activates pEMT in TECs via integrin signaling, as confirmed by in vitro and in vivo results. Repressing *Smoc2* affects total FAK levels in tubules after UUO but not in HK-2 cells. In fact, FAK expression increases after kidney injury (64), highlighting transcriptional regulation in addition to its activation by phosphorylation. These findings are consistent with studies highlighting the involvement of SMOC2 in integrin signaling in pathologies such as RCC, impairment of muscle stem cells, and CKD (33, 39, 49). We also found that SMOC2 and TGF-β1 exert additive effects on mesenchymal marker expression in HK-2 cells, acting through SMAD-independent and SMAD-dependent pathways, respectively. This suggests that the 2 ligands promote pEMT in TECs via distinct signaling mechanisms. SMOC2 can induce pEMT and activate fibroblasts, but Smoc2-tg mice don’t develop kidney fibrosis spontaneously (39). This phenomenon may be attributed to the low levels of integrins β1 and β3 in healthy kidneys, which are subsequently upregulated during injury. In the absence of these receptors, SMOC2 exerts minimal influence on healthy renal tissue. These integrins are markedly upregulated in TECs following injury, thereby elucidating the role of SMOC2 in maladaptive repair processes.

SMOC2 is a matricellular protein with 5 domains (34). The protein contains a FS domain with a Kazal-like structure, 2 TY domains, a SMOC-specific disordered domain, and an EC domain (34). In this study, the truncated SMOC2 mutant lacking the EC domain failed to induce mesenchymal markers in TECs, whereas the EC domain was sufficient to activate the pEMT properties of TECs. This indicates that the EC domain is the most active domain of SMOC2, consistent with previous studies in keratinocytes and osteoprogenitor cells (50, 65). We also showed that this domain enables SMOC2 to adhere to the ECM. Intriguingly, the FS domain is important in modulating SMOC2 activity. Although the SMOC2 mutant lacking this domain induces mesenchymal markers, it does not affect TEC migration, even in the presence of the EC domain. This indicates that the FS domain of SMOC2 may regulate the activity of its EC domain. It is conceivable that the FS domain plays a significant role in the physical interaction of SMOC2 between the cell and the ECM, thereby influencing cell migration. This is further supported by observations of the ΔFS mutant, which is partially released into the media and partially retained within the matrix, in contrast to the full-length protein. Protein interactions involving both domains may facilitate this process by orienting the EC domain in an optimal conformation for more effective binding to the matrix. In fact, SPARC (also known as BM-40) was found to adopt an active biological conformation when its EC domain interacts with its FS domain via a small, polar interface (66). The amino acids involved in this interaction are highly conserved (66). Similar interactions may occur in the SMOC2 protein, explaining the reduced activity of the EC domain in the absence of the FS domain. Although the EC domain plays a major role in SMOC2-regulated pEMT, other findings, such as cytoskeletal changes evaluated by phalloidin staining and the chemotaxis assessed by the transwell assay, reveal that each SMOC2 domain plays a substantial role in some pEMT-related processes.

Elucidating the role of pEMT in CKD has led to the discovery of its association with renal fibrosis and cell cycle arrest (13, 16, 43, 48). While the former is well characterized and consistent, the latter seems debatable, with evidence showing a direct link between pEMT and cell cycle arrest (13, 16), and other findings suggesting the independence of both processes (15). Further studies are needed to investigate whether SMOC2 is sufficient to induce cell cycle arrest or regulates cell cycle arrest in damaged TECs.

In conclusion, our results further elucidate the role of SMOC2 in maladaptive repair by providing evidence for its regulation of pEMT. We also propose a region-specific role of SMOC2 in kidney fibrosis, in which it mainly influences TECs in the cortex but potentially targets TECs and fibroblasts in the medulla. The effects of SMOC2 on TECs and its ability to bind to the ECM are dependent on the presence of the EC domain, with support from the FS domain. SMOC2 also binds and activates integrins implicated in kidney injury, highlighting the integrin pathway as a potential therapeutic strategy to protect the renal parenchyma after injury.

## Methods

### Sex as a biological variable.

All experiments were performed using male C57BL/6 mice to ensure consistency and reproducibility across the UUO, folic acid nephropathy, and ischemia-reperfusion models. Male mice were selected because they exhibit robust injury responses, particularly in ischemia-reperfusion, where female mice are relatively protected (67). The use of single sex also minimized variability from hormonal fluctuations. Kidney transplant biopsy specimens were collected from both male and female patients.

### Chemicals and reagents.

Ammonium persulfate (APS), Ponceau S, focal adhesion kinase inhibitor (324877-5MG), and tetramethylethylenediamine (TEMED) were purchased from Sigma-Aldrich. Acrylamide, ethylenediaminetetraacetic acid (EDTA), bis-acrylamide, sodium dodecyl sulfate (SDS), Tween 20, methanol, Tris-base, and glycine were purchased from VWR International. Rhodamine Phalloidin (R415), 10% buffered formalin, and 4% Paraformaldehyde (PFA) were purchased from Thermo Fisher Scientific. Ethanol (P016EAAN) was purchased from Greenfield Global. Cilengitide (HY-16141) was purchased from MedChem Express, and collagenase type 2 (LS004176) was purchased from Worthington.

### Animal models.

Male C57BL/6 mice aged 8–12 weeks were purchased from The Jackson Laboratory. Animals were housed at constant ambient temperature in a 12-hour light cycle and fed ad libitum. Folic acid nephropathy (FAN) was induced using a single i.p. injection of folic acid (250 mg/kg body weight; Sigma-Aldrich, F8758). Folic acid was dissolved in sodium bicarbonate at 12.5 mg/L, and control mice were injected with an equivalent volume of sodium bicarbonate. For UUO, mice were anesthetized with isoflurane 2% before the dorsal incision to expose the left kidney. The ureter of the exposed kidney was ligated using 4-0 silk sutures, while the contralateral kidney was used as a control. For ischemia-reperfusion injury (IRI), mice were injected with buprenorphine (0.05 mg/kg) for analgesia and were anesthetized with a mixture of ketamine (100 mg/kg) and xylazine (10 mg/kg). Bilateral ischemia-reperfusion was achieved after clamping the renal pedicles for 25 minutes. Control mice were sham operated. The body temperature of the mice was maintained at 37°C during all the surgeries. Mice were euthanized, and kidneys were isolated after 2 and 8 days for UUO and after 7 days for IRI and FAN. Kidneys were paraffin-embedded for immunofluorescence staining or frozen in liquid nitrogen for RNA and protein extraction.

### Isolation of kidney tubules.

Tubules were isolated from contralateral and injured kidneys after UUO using WT and Smoc2-KO mice. Fresh kidneys were cut into small pieces and digested in dissociation buffer (2 mg/mL collagenase type 2 in PBS) at 37°C with agitation. Digestion was monitored under a microscope every 10 minutes. Once complete, collagenase activity was halted with an equal volume of stop buffer (5% FBS in PBS). The mixture was sieved through 250 μm and 70 μm cell strainers to separate tubules from interstitial cells. The filtrates were centrifuged at 300*g* for 5 minutes. Pellets were suspended in TRIzol or RIPA with inhibitors for RNA and protein extraction.

### Immunofluorescence staining.

Paraffin-embedded kidney tissues were sectioned at 5 μm using a microtome. Kidney sections were deparaffinized and rehydrated. Epitope retrieval was performed using citrate buffer (10 mM citric acid and 0.05% Tween 20, pH 6.0) at 95°C for 30 minutes. After blocking with 5% donkey serum, the sections were incubated with the following antibodies: anti-vimentin (1: 50, Santa Cruz Biotechnology, sc-6260) for patients with CKD, anti-vimentin (1:50, R&D Systems, AF2105) for mouse tissues, anti-KIM1 (1:400, R&D Systems, AF1817-SP), anti-α-SMA (1: 800, Sigma, A2547), anti-SMOC2 (1:100, gift from Ursula Hartmann, University of Cologne, Köln, Germany). After washing with PBS, they were incubated with secondary antibodies conjugated with Alexa Fluor 647 or Cy3 (1:200, Jackson ImmunoResearch Laboratories). For immunofluorescence of cultured HK-2 cells, cells were seeded in an 8-well chamber (Ibidi, Lot number: 260303/3) prior to treatment with rhSMOC2 or rhTGF-β1. After 48 hours of treatment, cells were washed with PBS and fixed in 4% paraformaldehyde for 10 minutes. The cells were then permeabilized using 0.1% Triton-X for 10 minutes, washed, and blocked in donkey serum (10% serum in PBS) for 1 hour. Anti-TWIST1 (1:20, Abcam, ab50887) and anti-FOXK1 (1:50, Santa Cruz Biotechnology, sc-373810) were added in 5% donkey serum. After washing with PBS, the cells were incubated with secondary antibodies conjugated with Alexa Fluor 647 (1:200, Jackson ImmunoResearch Laboratories) prior to removing the chambers. Fluoroshield with DAPI (MilliporeSigma) was used for nuclear staining and mounting. Slides were imaged using a Zeiss AxioObserver.Z1 inverted microscope coupled to an X-Cite 120LED Boost High-Power LED illumination system.

### H&E staining.

Paraffin-embedded kidney tissues, sectioned at 5 μm, were deparaffinized and rehydrated. Microscopic slides were incubated in hematoxylin (Sigma Aldrich, GHS216) for 5 minutes, washed, and differentiated in acid alcohol (30 mM HCl in 70% ethanol) for 10 seconds. Slides were incubated in Scott’s tap water, washed, dehydrated, and counterstained with alcoholic eosin Y (Sigma Aldrich, HT110216-500ML) for 5 minutes. Mounting medium (Sigma Aldrich, 03989-500ML) was added, and slides were imaged using a Zeiss AxioObserver.Z1 inverted microscope coupled to an X-Cite 120LED Boost High-Power LED illumination system.

### PSR staining.

Paraffin-embedded kidney tissues, sectioned at 5 μm, were deparaffinized and rehydrated. Microscopic slides were incubated in hematoxylin (Sigma Aldrich, GHS216) for 8 minutes and washed in tap water. Slides were incubated in Sirius red solution (Sigma-Aldrich, 365548) for 1 hour and washed twice in acidified water for 5 minutes. Slides were dehydrated, and mounting medium (Sigma Aldrich, 03989-500ML) was added. Images were taken using a Zeiss AxioObserver.Z1 inverted microscope coupled to an X-Cite 120LED Boost High-Power LED illumination system.

### Coimmunoprecipitation.

HK-2 cells were seeded on 100 mm dishes and transfected with an empty vector or a vector with full-length SMOC2. Both vectors contained a Myc tag. Cells were lysed in an IPH buffer (50 mM Tris, pH 8, 150 mM NaCl, 5 mM EDTA, 0.5% NP-40) with protease and phosphatase inhibitors. The agarose c-myc beads (Sigma, A7470-1ML) were washed 5 times with 1 mL of cold PBS and resuspended in 50 μL of IPH buffer. For the immunoprecipitation, 0.5–1 mg of cell lysate was incubated with 50 μL of beads on a rotator at 4°C overnight. The beads were washed 3-4 times and centrifuged at 376*g* for 2 minutes. Sample buffer 2X was added to the beads, and the mixture was denatured at 95°C for 10 minutes. Mouse IgG was used as a loading control.

### Western blot analysis.

Cold radioimmunoprecipitation assay (RIPA) buffer (Thermo Fisher Scientific) containing phosphatase and protease inhibitors (Roche Diagnostics) was used to homogenize kidney tissues and cell cultures. The concentration of cellular lysate was determined using a bicinchoninic acid (BCA) protein assay kit (Pierce, 23225). Protein lysates (15 μg for cellular lysate and 40 μg for kidney lysates) were separated by electrophoresis on 7.5%–10% polyacrylamide gels containing 0.4% SDS, followed by transfer onto 0.22 μm nitrocellulose membranes (Amersham Protran, GE Healthcare) or polyvinylidene difluoride membranes (Bio-Rad). The following primary antibodies were used: anti-fibronectin (FN, 1:2000, Abcam, ab23750), anti-collagen I (1:500, SouthernBiotech, 1310-01), anti-E-cadherin (1:1000, Abcam, ab15148), anti-N-cadherin (1:1000, BD, 610920), anti-SPARC-related modular calcium binding 2 (SMOC2, 1:500, gift from Ursula Hartmann, University of Cologne, Köln, Germany), anti-periostin (1:500, Santa Cruz Biotechnology, sc-398631), anti-vimentin (1:500, Santa Cruz Biotechnology, sc-6260), anti-cytokeratin-18 (CK-18, 1:2000, Proteintech, 10830-1-AP), anti-aquaporin-1 (AQP1, 1:500, Santa Cruz Biotechnology, sc-25287), anti-Myc tag (1:1000, Cell Signaling Technology, 2276), anti-α-smooth muscle actin (α-SMA, 1:2000, Sigma, A2547), anti-phospho-focal adhesion kinase (pFAK (Y397), 1:1000, Cell Signaling Technology, 3283), anti-FAK (1:1000, Cell Signaling Technology, 3285), anti-phospho-paxillin (pPaxillin (Y118), 1:1000 Cell Signaling Technology, 2541), anti-paxillin (1:1000, Cell Signaling Technology, 2542), anti-integrin β1 (ITGB1, 1:2000, Cell Signaling Technology, 9699), anti-integrin β3 (ITGB3; 1:2000, Cell Signaling Technology, 13166), anti-phospho-smad3 (pSmad3, 1:2000, Cell Signaling Technology, 9520), anti-smad2/3 (Smad3, 1:2000, Cell Signaling Technology, 3102), anti-FOXK1 (1:500; Santa Cruz Biotechnology, sc-373810), anti-TWIST1 (1:500, Abcam, ab50887), anti-TGF-β1 (1:1000, R&D Systems, MAB2402), and anti-glyceraldehyde 3-phosphate dehydrogenase (GAPDH; 1:2000; Abcam, ab9485). Horseradish peroxidase-conjugated secondary antibodies against goat (Sigma, A5420), mouse (Santa Cruz Biotechnology, sc-516102), and rabbit (Santa Cruz Biotechnology, sc-2357) were used to detect the appropriate primary antibody. Bands were detected with the Clarity Max Western ECL Substrate from Bio-Rad Laboratories (Hercules, USA). Densitometry analyses were performed using ImageJ (NIH).

### qPCR.

RNA was extracted from murine kidneys or HK-2 cells using TRIzol (Invitrogen, Burlington, ON, Canada) and then purified using the RNeasy Mini kit (Qiagen, Toronto, ON, Canada) according to the manufacturer’s protocol; 1 μg of total RNA was reverse transcribed into cDNA using SuperScript VILO cDNA Synthesis kit with ezDNase (Invitrogen). qPCR was performed using SsoAdvanced Universal SYBR Green Supermix (Bio-Rad) on an ABI 7500 Real-Time PCR System (Applied Biosystems). The list of mouse-specific primers is provided in Supplemental Table 1. We used 6 biological replicates for the mice’s kidney tissues, with technical triplicates for each reaction. Primer amplification efficiencies were calculated for each gene, and changes in the mRNA expression were determined using the Pfaffl method. The expression of each gene of interest was normalized against the expression of the housekeeping gene glyceraldehyde-3-phosphate dehydrogenase (*Gapdh*).

### Cell culture.

The human proximal tubule cell line (HK-2, derived from a male normal kidney), the human embryonic kidney cell (HEK293T), and the rat kidney fibroblast (NRK-49F, derived from a normal rat kidney) were obtained from the American Type Culture Collection (ATCC, USA) and maintained in a humidified atmosphere of 5% CO_2_ at 37°C. HK-2 cells were cultured with Dulbecco’s Modified Eagle’s Medium/F12 (DMEM/F12) supplemented with 10% heat-inactivated fetal bovine serum (FBS) (Gibco/Life Technologies). HEK293T cells were maintained in DMEM supplemented with 10% heat-inactivated FBS, whereas NRK-49F cells were cultured in DMEM supplemented with 5% heat-inactivated fetal calf serum (FCS).

### Cell treatment.

HK-2 cells were seeded in 6-well plates at densities adjusted according to the intended harvesting time (2 × 10^5^ cells for 16 h to 48 h, 1 × 10^5^ cells for 72 h) and allowed to adhere to the plates for 5 h. After adhesion, the media was replaced by DMEM/F12 supplemented with 1% FBS overnight. Cells were treated with fresh DMEM/F12 + 1% FBS containing the appropriate concentration of rhSMOC2 (R&D Systems, 5140-SM) or rhTGF-β1 (PeproTech, 100-21C). The treatment was replenished every 24 hours. For FAKi and rhSMOC2 cotreatment, cells were seeded as previously described and pretreated with 10 μM FAKi for 5 hours before rhSMOC2 treatment. The media were replaced after 24 hours with fresh DMEM/F12 + 1% FBS containing FAKi and rhSMOC2 at the appropriate concentration. For cilengitide treatment prior to rhSMOC2 stimulation, cells were seeded and exposed to a gradient of cilengitide (1 nM, 2.5 nM, 5 nM, 10 nM, 15 nM). The media were replaced after 24 hours with fresh DMEM/F12 + 1% FBS containing cilengitide and rhSMOC2 at the appropriate concentration.

### Conditioned media treatment.

HK-2 cells were seeded in 15 cm dishes and treated with vehicle or rhSMOC2 for 48 hours. NRK-49F cells were plated in parallel in 6-well plates containing DMEM + 5% FCS. Enrichment of secreted factors was performed in serum-free DMEM for 24 hours. HK-2 cell media were collected and centrifuged at 1,801*g* for 10 minutes on the day of treatment. The HK-2 cells in the dishes were trypsinized and counted. The volume of conditioned media were adjusted to account for the effects of 7 × 10^5^ HK-2 cells per condition. Fetal calf serum was added to reach a final concentration of 0.5%. The fibroblasts were stimulated for 24 and 48 hours.

### Cell transfection.

HK-2 cells were seeded in 6-well plates (2 × 10^5^ cells per well) in DMEM/F12 + 10% FBS. Twenty-four hours later, 2 μg of DNA was transfected using PolyJet (SignaGen Laboratories, SL100688) according to the manufacturer’s protocol. The SMOC2-Myc was purchased from Origene (RC211979), and the SMOC2 truncated mutants were a gift from Ursula Hartmann’s laboratory. For siRNA transfection, 80 pmol of scrambled (Santa Cruz Biotechnology, sc-37007), integrin β1 (Santa Cruz Biotechnology, sc-35674), or integrin β3 (Santa Cruz Biotechnology, sc-29375) siRNA was transfected in cells using Lipofectamine 2000 (Invitrogen, 52887). The media were replaced after 5 hours with DMEM/F12 + 10% FBS.

### Production of control vectors.

The vector containing the ΔEC mutant was digested using NheI (New England Biolabs, R3131S) and NotI (New England Biolabs, R3189S) in rCutSmart Buffer according to the manufacturer’s protocol. Blunt ends and ligation were generated using the Quick Blunting and Quick Ligation Kits (New England Biolabs, E0542S). Competent bacteria were transformed to amplify the vector. Vector purification was done using the PureLink HiPure Plasmid Filter Midiprep Kit (Invitrogen, K210014).

### Lentiviral transduction.

HEK293T cells were seeded in a 15 cm dish at 50% confluency to produce lentiviral particles. Twenty-four hours later, cells were cotransfected with 1.25 μg of pMD2.G (Addgene, 12259), 3.75 μg of psPAX2 (Addgene, 12260), and 6 μg of pLK0.1-puro (Addgene, 8453/Control) or 6 μg of pLenti-C-mGFP-P2A-Puro-SMOC2 (Origene, RC2111979L4/SMOC2) using Lipofectamine 2000. The medium was replaced 6 hours after transfection. Viral supernatant was collected after 48 hours and filtered with a 0.45 μm PVDF syringe filter. HK-2 cells were transduced using 8 mL of lentivirus-containing medium with 10% FBS supplemented with polybrene at 8 μg/mL. After 24 hours of transduction, the medium was replaced with fresh medium, and the selection of positive clones was done using puromycin at 2 μg/mL for 72 hours. Cells were split and maintained in puromycin for 5 days to finalize the selection.

### Phalloidin staining.

HK-2 cells in 12-well plates were washed twice with prewarmed PBS (pH 7.4) and fixed in 4% paraformaldehyde for 10 minutes at room temperature. The cells were washed twice with PBS and incubated with PBS containing 1% bovine serum albumin (BSA) for 30 minutes. Phalloidin 1× (Thermo Fisher Scientific, R415) in PBS with 1% BSA was added and incubated for 20 minutes at room temperature. The cells were washed twice with PBS, and DAPI was added to stain the nucleus before measuring fluorescence.

### Quantification of phalloidin staining.

F-actin distribution was quantified using the protocol described by Zonderland et al. (68). The ImageJ software was used to assess F-actin distribution in HK-2 cells. Channels were split to separate DAPI (blue) and phalloidin (red). The red channel was used in the analysis. The channel type was changed to 8-bit, and the scale was set in microns using the scale bar displayed on the microscopic image. A default threshold was applied to the 8-bit image to isolate actin fibers and create a binary image. A straight line was drawn across the cell, from edge to edge, and the plot profile was obtained. The data representing actin intensity across the line were exported and analyzed using GraphPad Prism, version 8. A 2-way ANOVA was used to determine statistical significance for the F-actin distribution (Interaction) and overall F-actin intensity (column factor). The average profile of 10 cells was used for each condition, and data are presented at the highest common distance across the cells analyzed.

### Transwell assay.

HK-2 cells were seeded in 6-well plates for transfection and trypsinized on the day of the migration assay. Cells were counted, resuspended in DMEM/F12 + 1% FBS, and put into 8 μm Falcon inserts (Corning, 353097). For rhSMOC2-treated cells, the appropriate concentration of recombinant protein was added to the media before resuspension. The inserts were put in a 24-well plate with DMEM/F12 + 10% FBS in the lower chamber. The cells were incubated at 37°C for the indicated times. The inserts were washed with PBS, fixed in 10% formalin for 10 minutes, and stained with 0.5% crystal violet for thirty minutes. The inserts were washed with water and left to dry before images were taken.

### Scratch assay.

HK-2 cells, ranging from 6 × 10^3^ to 2.4 × 10^4^ cells, were seeded in a 96-well plate and allowed to adhere. The scratch was made with a sterile Incucyte Woundmaker Tool. The plate was washed to remove floating cells, and media were added to the cells. For rhSMOC2-treated cells, DMEM/F12 supplemented with 1% FBS was used, with or without the recombinant protein. Images were taken every 3 or 4 hours with 10× objectives of the Incucyte Live-Cell Imaging and Analysis System. Cell migration analysis was performed using the Scratch Wound Analysis Software Module.

### ECM isolation.

ECM isolation was performed as described by Fu et al. (69). HK-2 cells were seeded in 100 mm dishes for ECM production and transfected for 48 hours. ECM isolation was achieved using EGTA (5 × 10^-4^ M; pH 7.4) and constant agitation at 4°C. Cells were washed with PBS after 2 hours, and the chelating agent was replenished until the cells were fully detached. The plates were allowed to dry, and the ECM was recovered in 100 μL of RIPA buffer with protease and phosphatase inhibitors.

### RNA-seq analysis.

Bioinformatic analyses were performed at the Bioinformatics core facility of the Montreal Clinical Research Institute (IRCM). The differential expression of genes from the raw alignment was calculated using DESeq2. *P* values were adjusted for multiple testing using the Benjamini-Hochberg FDR, and shrunken log_2_ fold changes were obtained using DESeq2’s shrinkage estimator. Functional enrichment analyses of DEGs (gene ontology and pathway enrichment) were performed with the gprofiler2 R package. GSEA 4.3.2 was used to perform the GSEA Preranked with M5 and MH gene sets from 2024 Mus musculus MSigDB. For single-cell RNA-seq on mouse kidney injury models, data for integrin expression were retrieved from the Kidney Interactive Transcriptomics (KIT) database of the Humphreys laboratory (http://humphreyslab.com/SingleCell/) (Washington University in St. Louis, St. Louis, Missouri, USA). Analyses were performed by the Software from Wu et al. (70). For the expression of integrin genes in HK-2 cells, we used the publicly available database of the Epithelial Systems Biology Laboratory (ESBL) for the major proximal tubule cell culture models commonly used in kidney research.

### Statistics.

All experimental data were obtained from at least 3 independent experiments and presented as mean ± SD. Differences between the 2 groups were determined using an unpaired 2-tailed Student’s *t* test with Welch’s correction. Differences between multiple groups were assessed using a 1-way ANOVA. GraphPad Prism, version 8, was used for statistical analyses and graph generation. *P* < 0.05 was considered statistically significant.

### Study approval.

Experiments in vivo were carried out according to the Canadian Council on Animal Care guidelines for the use of laboratory animals under the supervision and approval of our local animal care committee (Comité Protection des Animaux du Centre Intégré Universitaire de Santé et de Services Sociaux [CIUSSS] de l’Est-de-l’ile-de-Montréal) with the approved protocol nos. 2022-2895 and 2022-2908 in compliance with the Animal Research: Reporting of in vivo Experiments (ARRIVE) Guidelines.

### Ethical approval.

Deidentified renal biopsy specimens from kidney transplant patients (*n* = 10) were obtained after the completion of the diagnosis by the Pathology Department (diagnosis: interstitial fibrosis and tubular atrophy score = 1/3). All patient recruitment procedures, including consent to use renal biopsy samples for research purposes, were approved by the Maisonneuve-Rosemont Hospital Research Ethics Board (no. 2023-3307), and written informed consent was obtained from each patient.

### Data availability.

The dataset for transgenic mice overexpressing *Smoc2* is available in the NCBI GEO database under accession number GSE85209. All data used to generate the graphs in this study are available in the Supporting Data Values file.

## Author contributions

SC contributed conceptualization, investigation, methodology, formal analysis, and review and editing. NH contributed investigation and methodology. PG contributed investigation and methodology. CL contributed methodology and formal analysis. XSL contributed conceptualization. FAM contributed funding acquisition (coapplicant) and investigation. JBC contributed conceptualization, investigation, formal analysis, and review and editing. CG contributed conceptualization, supervision, funding acquisition, and review and editing.

## Funding support

- Cancer Research Society and the Kidney Cancer Research Network of Canada (cofunded Operating Grant Program #24347 to CG).
- Canadian Institutes of Health Research (CIHR Project Grant #428250 to primary investigator CG, coapplicant FAM).
- Kidney Health Research Grant (674586-21KHRG).
- Natural Sciences and Engineering Research Council of Canada (NSERC Discovery Grant #DGECR-2019-00141 to CG).
- Hôpital Maisonneuve-Rosemont Foundation (to CG).
- Fonds de recherche du Québec – Santé (Bourses de chercheurs-boursiers Junior 1 # 298956 and Junior 2 # 367954 (to CG.)
- Fonds de recherche du Québec - Santé (Bourse de formation de doctorat #351233) (to SC).

## Supplementary Material

Supplemental data

Unedited blot and gel images

Supporting data values

## Version 1 08/12/2026

In-Press Preview

## Version 2 09/22/2026

Electronic publication
