# Supplementary material for: SMOC2 promotes partial epithelial-mesenchymal transition and maladaptive repair in renal tubular epithelial cells
Source: JCI Insight. 2026 Aug 11;11(18):e198555. doi: 10.1172/jci.insight.198555 (PMC13596733; doi:10.1172/jci.insight.198555)
Supplement: Supplementary file 1 [file jciinsight-11-198555-s041.pdf]

A

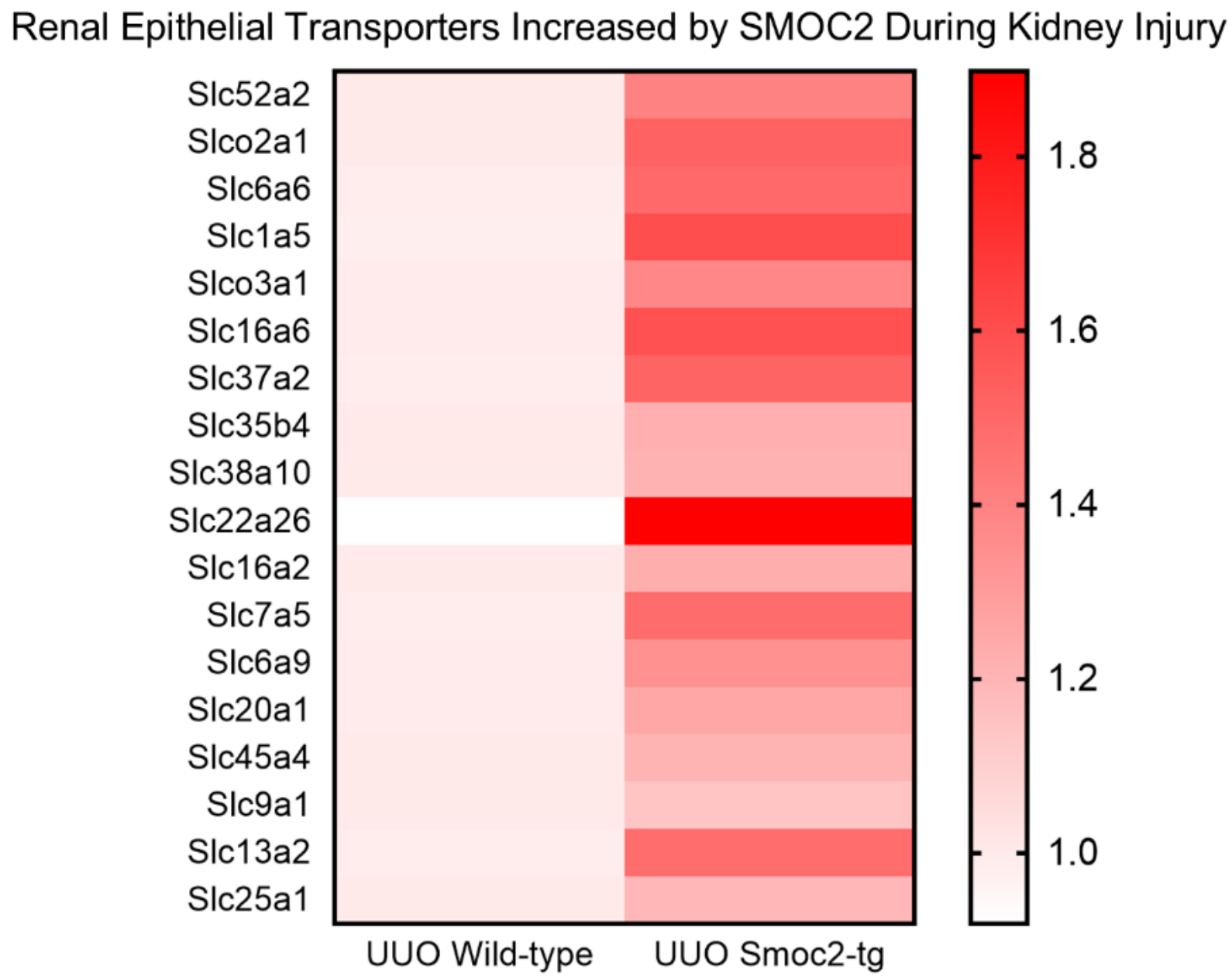

Renal Epithelial Transporters Increased by SMOC2 During Kidney Injury
Slc52a2
Slco2a1
Slc6a6
Slc1a5
Slco3a1
Slc16a6
Slc37a2
Slc35b4
Slc38a10
Slc22a26
Slc16a2
Slc7a5
Slc6a9
Slc20a1
Slc45a4
Slc9a1
Slc13a2
Slc25a1
UUO Wild-type
UUO Smoc2-tg
1.8
1.6
1.4
1.2
1.0

B

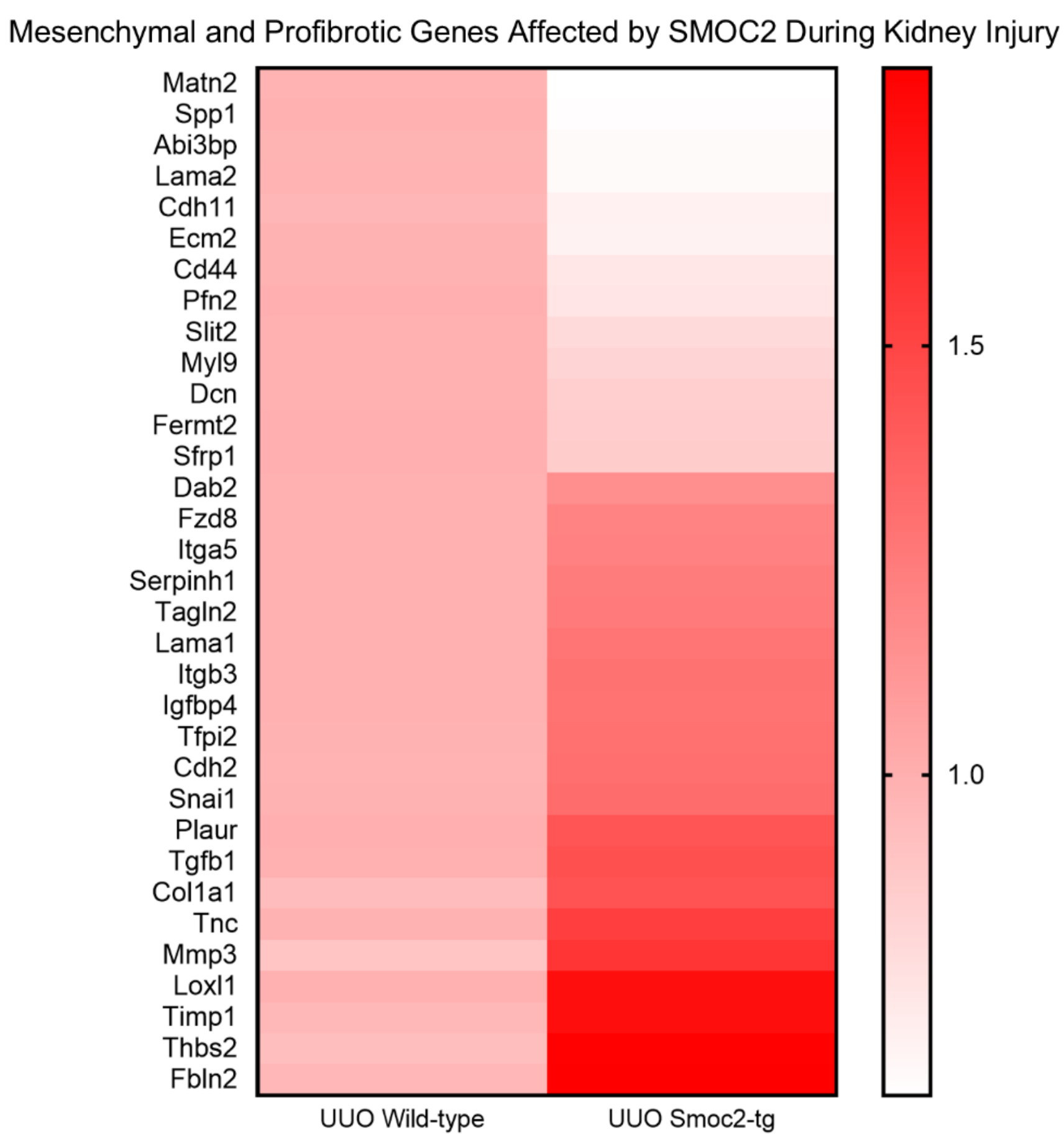

Mesenchymal and Profibrotic Genes Affected by SMOC2 During Kidney Injury
Matn2
Spp1
Abi3bp
Lama2
Cdh11
Ecm2
Cd44
Pfn2
Slit2
Myl9
Dcn
Fermt2
Sfrp1
Dab2
Fzd8
Itga5
Serpinh1
Tagln2
Lama1
Itgb3
Igfbp4
Tfpi2
Cdh2
Snai1
Plaur
Tgfb1
Col1a1
Tnc
Mmp3
Loxl1
Timp1
Thbs2
Fbln2
UUO Wild-type
UUO Smoc2-tg
1.5
1.0

Figure S1: Persistent *Smoc2* expression affects kidney epithelial transporters after UUO and is associated with the expression of mesenchymal and fibrosis-related genes. A) Heatmap displaying the relative expression of increased kidney epithelial transporter genes in *Smoc2*-transgenic (Smoc2-tg) mice compared to wild-type littermates seven days after UUO. **B)** Heatmap showing the relative expression of mesenchymal and profibrotic genes in wild-type or *Smoc2*-transgenic (Smoc2-tg) seven days after UUO.

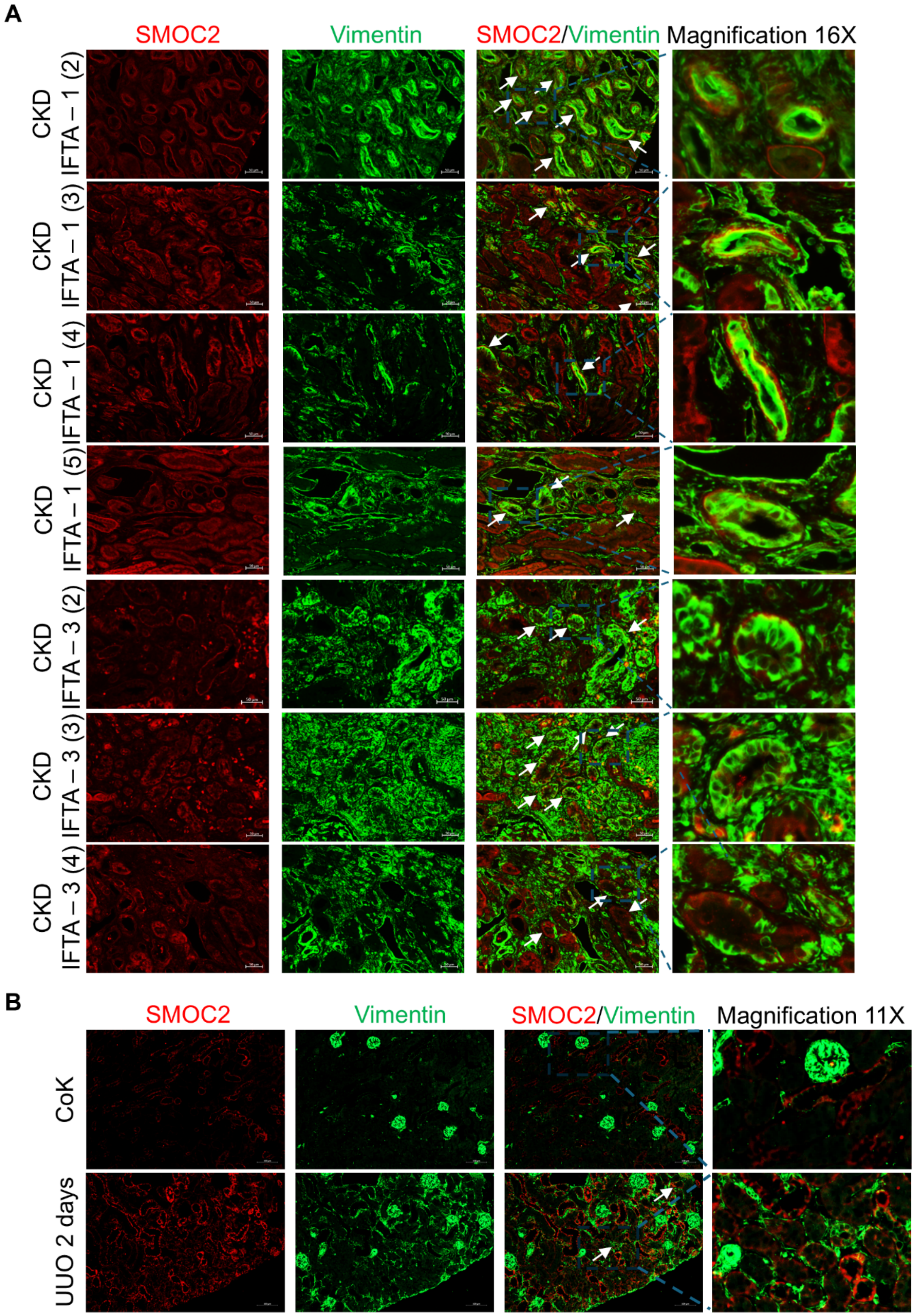
A
SMOC2
Vimentin
SMOC2/Vimentin
Magnification 16X
CKD IFTA – 1 (2)
CKD IFTA – 1 (3)
CKD IFTA – 1 (4)
CKD IFTA – 1 (5)
CKD IFTA – 3 (2)
CKD IFTA – 3 (3)
CKD IFTA – 3 (4)
B
SMOC2
Vimentin
SMOC2/Vimentin
Magnification 11X
CoK
UUO 2 days

Figure S2: SMOC2 localization is associated with tubular expression of vimentin in CKD patients, with little colocalization in UUO. A) Immunofluorescence showing the localization of SMOC2 and vimentin in CKD patients with a score of 1 or 3 for interstitial fibrosis and tubular atrophy (IFTA). **B)** Immunofluorescence showing the localization of SMOC2 and vimentin in the UUO model of kidney injury after 2 days. The arrows point to tubules positive for SMOC2 and vimentin, while the dashed boxes indicate the magnified regions. Images were taken using a 10X objective.

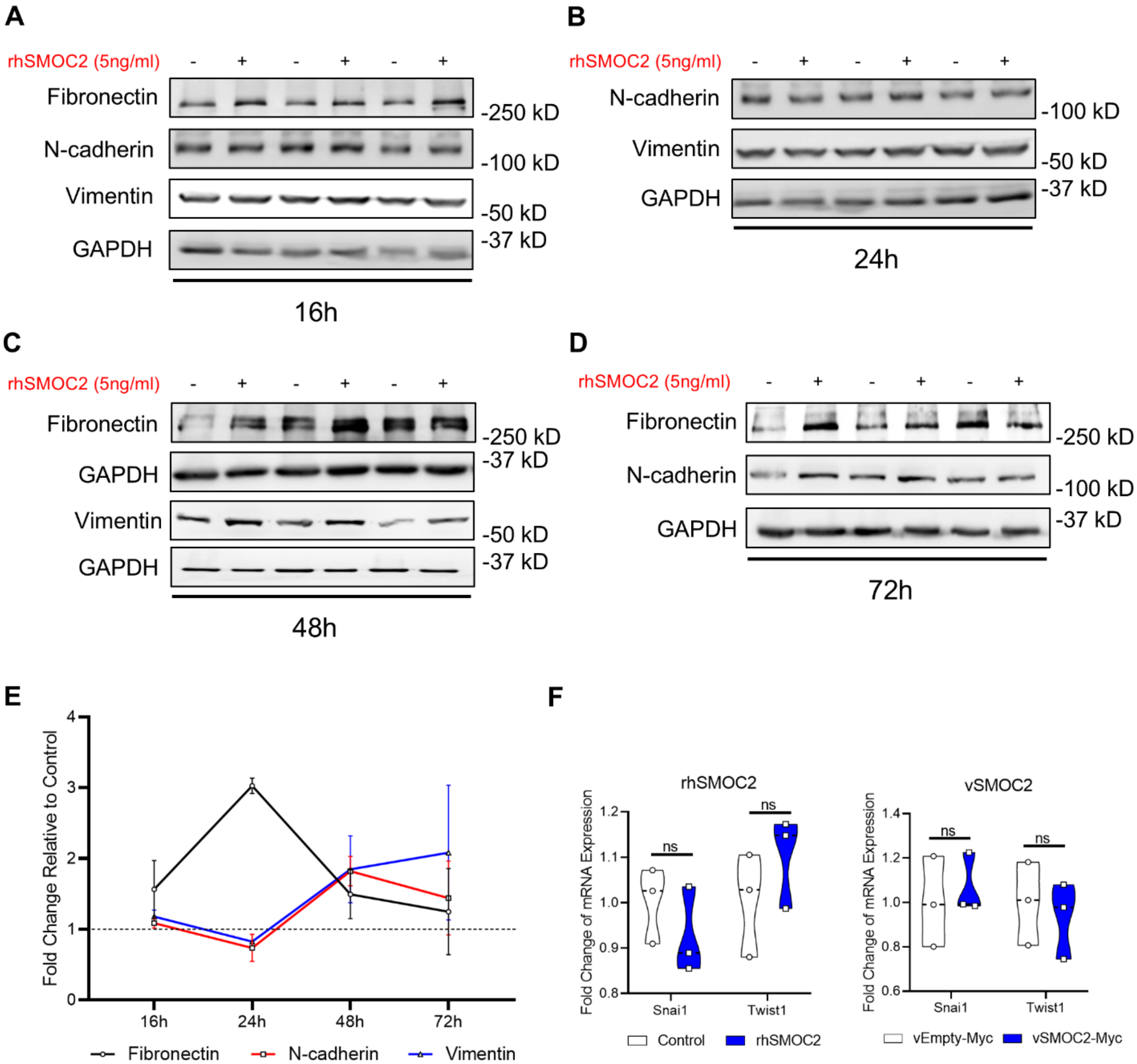
A
rhSMOC2 (5ng/ml)
- + - + - +
Fibronectin
N-cadherin
Vimentin
GAPDH
-250 kD
-100 kD
-50 kD
-37 kD
16h
B
rhSMOC2 (5ng/ml)
- + - + - +
N-cadherin
Vimentin
GAPDH
-100 kD
-50 kD
-37 kD
24h
C
rhSMOC2 (5ng/ml)
- + - + - +
Fibronectin
GAPDH
Vimentin
GAPDH
-250 kD
-37 kD
-50 kD
-37 kD
48h
D
rhSMOC2 (5ng/ml)
- + - + - +
Fibronectin
N-cadherin
GAPDH
-250 kD
-100 kD
-37 kD
72h
E
Fold Change Relative to Control
0
1
2
3
4
16h
24h
48h
72h
Fibronectin
N-cadherin
Vimentin
F
rhSMOC2
Fold Change of mRNA Expression
0.8
0.9
1.0
1.1
1.2
ns
ns
Snai1
Twist1
Control
rhSMOC2
vSMOC2
Fold Change of mRNA Expression
0.6
0.8
1.0
1.2
1.4
ns
ns
Snai1
Twist1
vEmpty-Myc
vSMOC2-Myc

Figure S3: SMOC2 dynamically regulates mesenchymal markers in HK-2 cells with no effect on the gene expression of pEMT transcription factors. A-D. Western blots showing the levels of mesenchymal markers after 16h (A), 24h (B), 48h (C), and 72h (D) of rhSMOC2 stimulation of HK-2 cells. These markers come from the same experiments as Figure 5. **E)** Graph showing the expression of mesenchymal markers across the time points analyzed. **F)** Violin plots showing the gene expression measured by qPCR of *SNAI1* and *TWIST1* after stimulation with rhSMOC2 or SMOC2 ectopic expression in HK-2 cells for 16h. The data was analyzed using an unpaired two-tailed Welch's t-test. Gene expression was normalized to the housekeeping gene *GAPDH*.

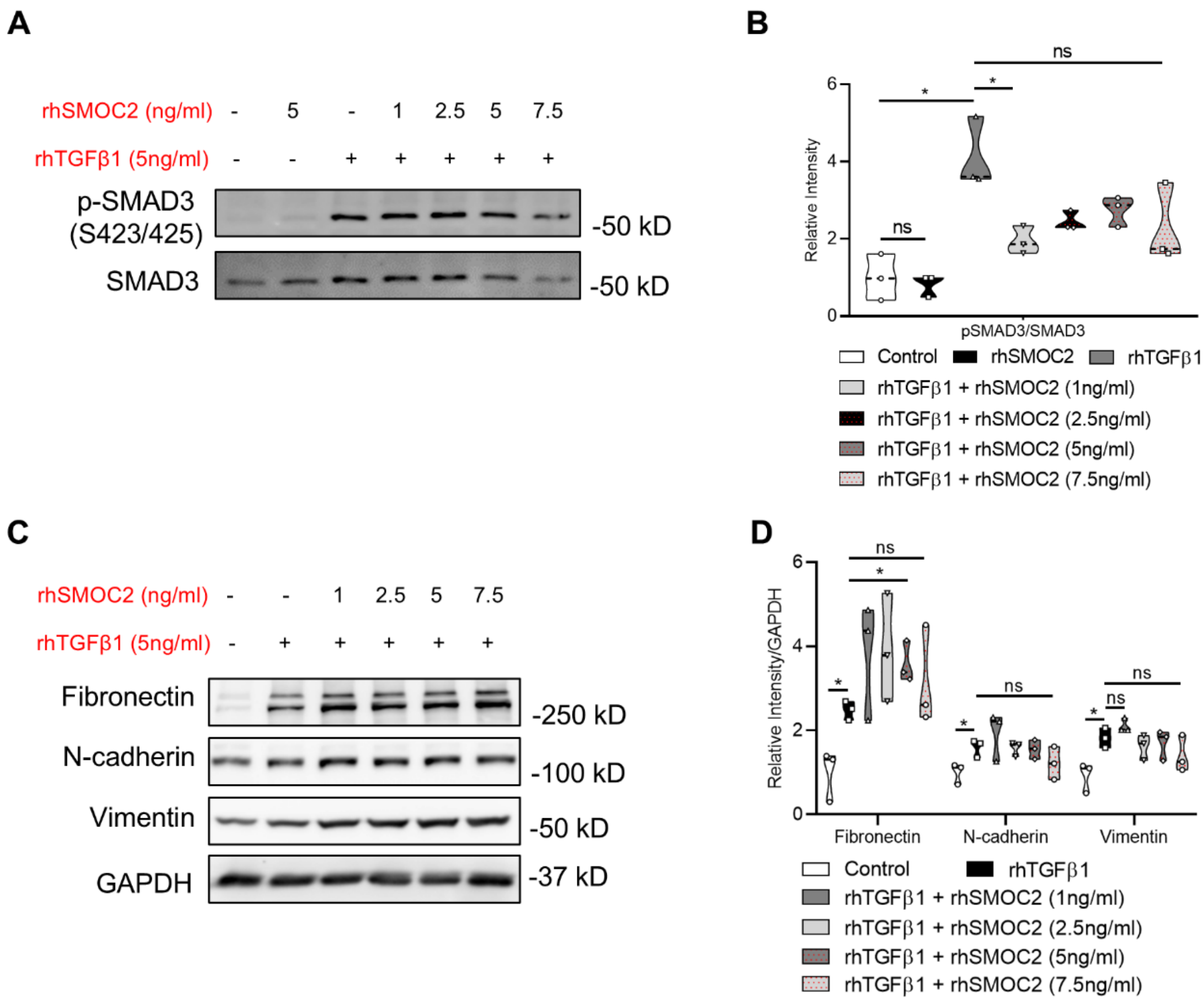
A
rhSMOC2 (ng/ml) - 5 - 1 2.5 5 7.5
rhTGFβ1 (5ng/ml) - - + + + + +
p-SMAD3 (S423/425)
SMAD3
-50 kD
-50 kD
B
Relative Intensity
pSMAD3/SMAD3
ns
*
Control
rhSMOC2
rhTGFβ1
rhTGFβ1 + rhSMOC2 (1ng/ml)
rhTGFβ1 + rhSMOC2 (2.5ng/ml)
rhTGFβ1 + rhSMOC2 (5ng/ml)
rhTGFβ1 + rhSMOC2 (7.5ng/ml)
C
rhSMOC2 (ng/ml) - - 1 2.5 5 7.5
rhTGFβ1 (5ng/ml) - + + + + +
Fibronectin
N-cadherin
Vimentin
GAPDH
-250 kD
-100 kD
-50 kD
-37 kD
D
Relative Intensity/GAPDH
Fibronectin
N-cadherin
Vimentin
ns
*
Control
rhTGFβ1
rhTGFβ1 + rhSMOC2 (1ng/ml)
rhTGFβ1 + rhSMOC2 (2.5ng/ml)
rhTGFβ1 + rhSMOC2 (5ng/ml)
rhTGFβ1 + rhSMOC2 (7.5ng/ml)

Figure S4: SMOC2 and TGFβ1 have additive effects on pEMT markers. A) Western blots showing the protein levels of phosphorylated SMAD3 (pSMAD3) and its total levels in HK-2 cells stimulated with rhTGFβ1, rhSMOC2, or with rhTGFβ1 and a gradient of rhSMOC2 for one hour. **B)** Quantification of the western blots in (A). **C)** Western blots showing the levels of mesenchymal markers in HK-2 cells stimulated with rhTGFβ1 alone or combined with a gradient of rhSMOC2 for 48 hours. **D)** Quantification of the western blots in (C). Data are presented as mean ± SD; n=3 for each experiment and analyzed using a one-way ANOVA. *$P<0.05$, ns: not significant.

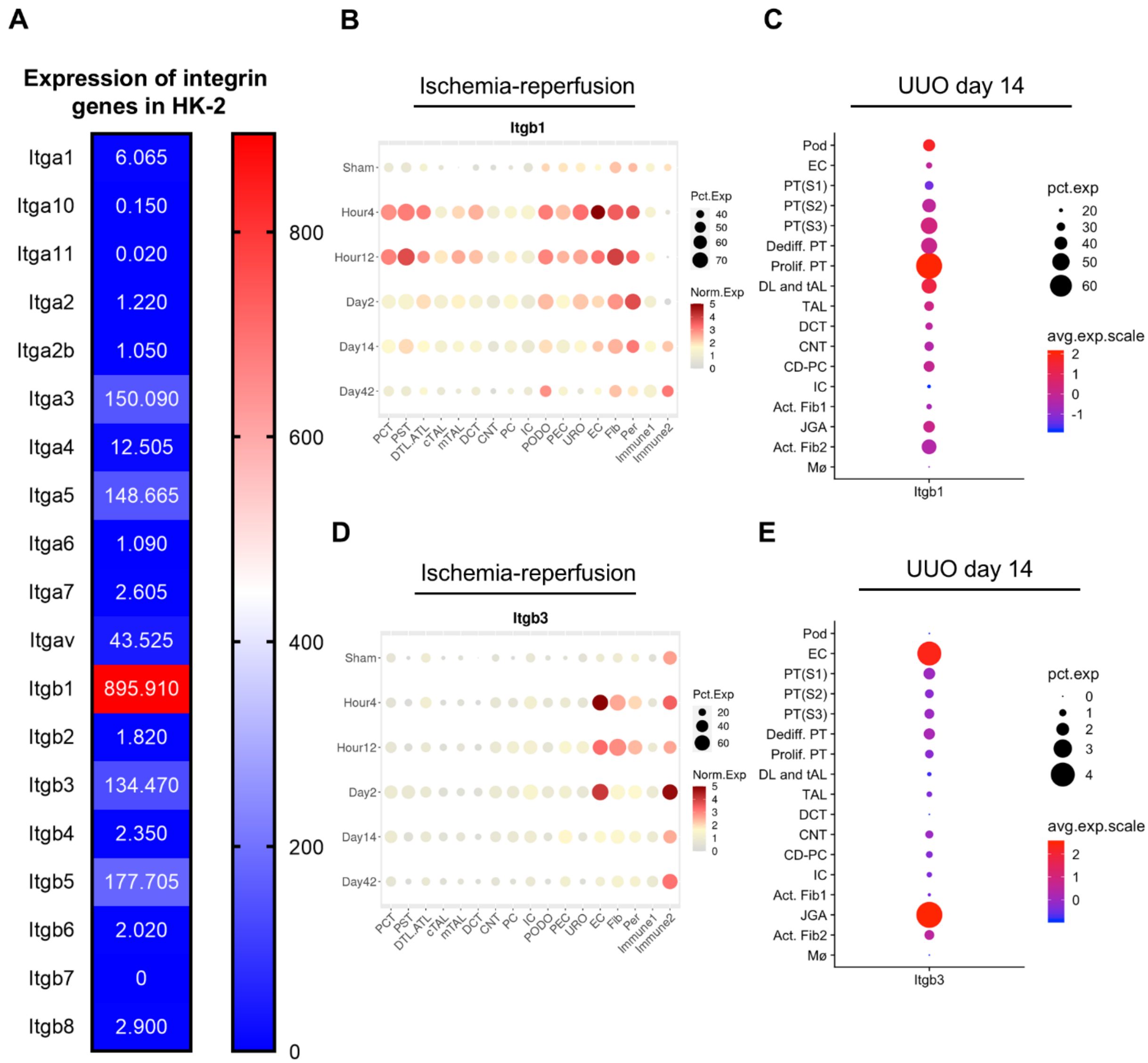
A
Expression of integrin genes in HK-2
Itga1 6.065
Itga10 0.150
Itga11 0.020
Itga2 1.220
Itga2b 1.050
Itga3 150.090
Itga4 12.505
Itga5 148.665
Itga6 1.090
Itga7 2.605
Itgav 43.525
Itgb1 895.910
Itgb2 1.820
Itgb3 134.470
Itgb4 2.350
Itgb5 177.705
Itgb6 2.020
Itgb7 0
Itgb8 2.900
800
600
400
200
0
B
Ischemia-reperfusion
Itgb1
Sham
Hour4
Hour12
Day2
Day14
Day42
PCT PST DTL.ATL cTAL mTAL DCT CNT PC IC PODO PEC URO EC Fib Per Immune1 Immune2
Pct.Exp
Norm.Exp
C
UUO day 14
Pod
EC
PT(S1)
PT(S2)
PT(S3)
Dediff. PT
Prolif. PT
DL and tAL
TAL
DCT
CNT
CD-PC
IC
Act. Fib1
JGA
Act. Fib2
Mø
Itgb1
pct.exp
avg.exp.scale
D
Ischemia-reperfusion
Itgb3
Sham
Hour4
Hour12
Day2
Day14
Day42
PCT PST DTL.ATL cTAL mTAL DCT CNT PC IC PODO PEC URO EC Fib Per Immune1 Immune2
Pct.Exp
Norm.Exp
E
UUO day 14
Pod
EC
PT(S1)
PT(S2)
PT(S3)
Dediff. PT
Prolif. PT
DL and tAL
TAL
DCT
CNT
CD-PC
IC
Act. Fib1
JGA
Act. Fib2
Mø
Itgb3
pct.exp
avg.exp.scale

Figure S5: Expression patterns of major integrins expressed in TECs in normal conditions and different models of kidney injury. A) Heatmap displaying the logTPM for the expression of integrins in HK-2 cells under normal conditions. **B)** and **C)** Dot plot of single-cell RNA sequencing showing the expression of *Itgb1* across multiple kidney cell types after ischemia-reperfusion (B) or 14 days after UUO (C). **D)** and **E)** Dot plot showing the expression of *Itgb3* across multiple kidney cell types after ischemia-reperfusion (D) or 14 days after UUO (E). The analyses of the single-cell RNA-sequencing data were performed by the Software of the Kidney Interactive Transcriptomics. Pod: podocytes, EC: endothelial cells, PT: proximal tubules, DL: descending limb, tAL: thin ascending limb, TAL: thick ascending limb, DCT: distal convoluted tubules, CNT: connecting tubules, CD: collecting ducts, PC: principal cells, IC: intercalated cells, Fib1: fibroblasts 1, JGA: juxtaglomerular apparatus, Fib2: fibroblasts 2, MΦ: macrophages; Dediff. : dedifferentiated, Prolif. : proliferating, Act. : activated.

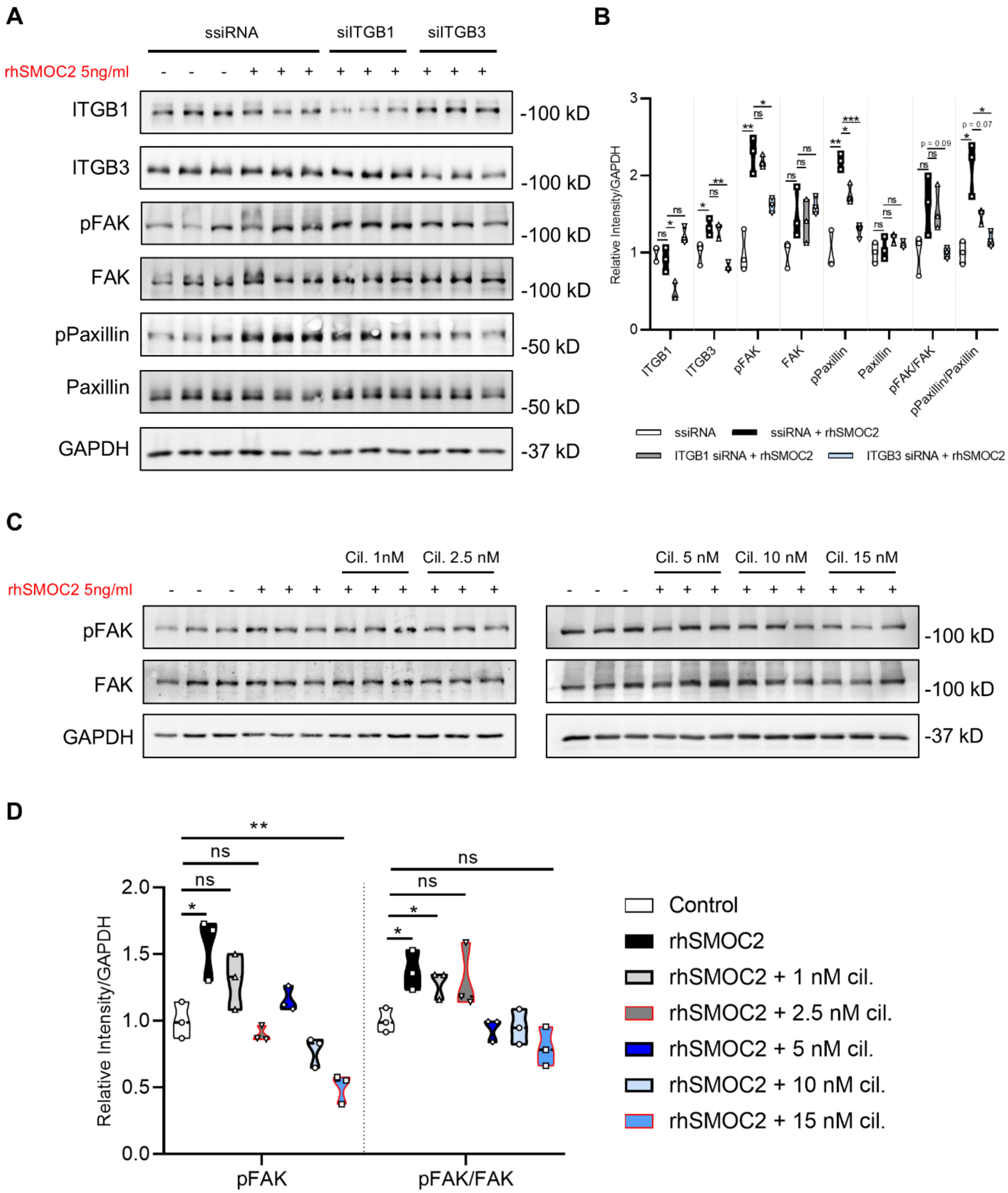
A
ssiRNA
siITGB1
siITGB3
rhSMOC2 5ng/ml
ITGB1
ITGB3
pFAK
FAK
pPaxillin
Paxillin
GAPDH
-100 kD
-100 kD
-100 kD
-100 kD
-50 kD
-50 kD
-37 kD
B
Relative Intensity/GAPDH
ITGB1
ITGB3
pFAK
FAK
pPaxillin
Paxillin
pFAK/FAK
pPaxillin/Paxillin
p = 0.09
p = 0.07
ssiRNA
ssiRNA + rhSMOC2
ITGB1 siRNA + rhSMOC2
ITGB3 siRNA + rhSMOC2
C
Cil. 1nM
Cil. 2.5 nM
Cil. 5 nM
Cil. 10 nM
Cil. 15 nM
rhSMOC2 5ng/ml
pFAK
FAK
GAPDH
-100 kD
-100 kD
-37 kD
D
Relative Intensity/GAPDH
pFAK
pFAK/FAK
Control
rhSMOC2
rhSMOC2 + 1 nM cil.
rhSMOC2 + 2.5 nM cil.
rhSMOC2 + 5 nM cil.
rhSMOC2 + 10 nM cil.
rhSMOC2 + 15 nM cil.

Figure S6: Integrin β3 is a major contributor to the signal transduction in HK-2 cells upon rhSMOC2 stimulation. A) Western blot showing the levels of integrin signaling molecules in HK-2 cells stimulated with rhSMOC2 and transfected with a scrambled siRNA (ssiRNA), or siRNA targeting integrin β1 (siITGB1) or β3 (siITGB3). **B)** Violin plots showing the quantification of the western blot in (A). **C)** Western blot showing the level of phosphorylated FAK (pFAK) and its total level in HK-2 cells stimulated with rhSMOC2 with or without a gradient of cilengitide (cil.). **D)** Violin plots showing the quantification of the western blot in (C). The data was analyzed using a one-way ANOVA. *$P<0.05$, **$P<0.01$, ***$P<0.001$; n = 3 for each experiment.

Table S1. List of primers used for qPCR analyses

**Genes**	**Forward (5’ to 3’)**	**Reverse (5’ to 3’)**
Gapdh	CGTGGAGTCTACTGGTGTCTTCA	GGCGGAGATGATGACCCTTT
Cdh16	TGTTATTCAGCTACCACTACCCC	CTAGGCTGGTGCTTCCACTG
Vil1	CCTTGAGACCCCTATCATCGTG	GCTCATAACCTCGTCAGCAATC
Col1a1	TGACTGGAAGAGCGGAGAGTA	GAGGGAACCAGATTGGGGTG
Smoc2	GGAGCAGGGAAAGCAGATGAT	GAGTGTAGGGTAGCGTGAGG
Tgfb1	CCGAAGCGGACTACTATGCTAA	TTCTCATAGATGGCGTTGTTGC
Twist1	GGCCGGAGACCTAGATGTCATT	CTGGGAATCTCTGTCCACGG
Snai1	TGTGTGGAGTTCACCTTCCAGC	CCAGGAGAGAGTCCCAGATGAG
SNAI1	TAGCGAGTGGTTCTTCTGCG	TGCTGGAAGGTAAACTCTGGA
TWIST1	TCGGACAAGCTGAGCAAGAT	TTCTCTGGAAACAATGACATCTAGG
GAPDH	GTCGGAGTCAACGGATT	AAGCTTCCCGTTCTCAG
