# Supplementary material for: SMOC2 promotes partial epithelial-mesenchymal transition and maladaptive repair in renal tubular epithelial cells
Source: JCI Insight. 2026 Aug 11;11(18):e198555. doi: 10.1172/jci.insight.198555 (PMC13596733; doi:10.1172/jci.insight.198555)

Raw Data for Western Blots

SMOC2-pEMT

2025-07-28

Figure 3

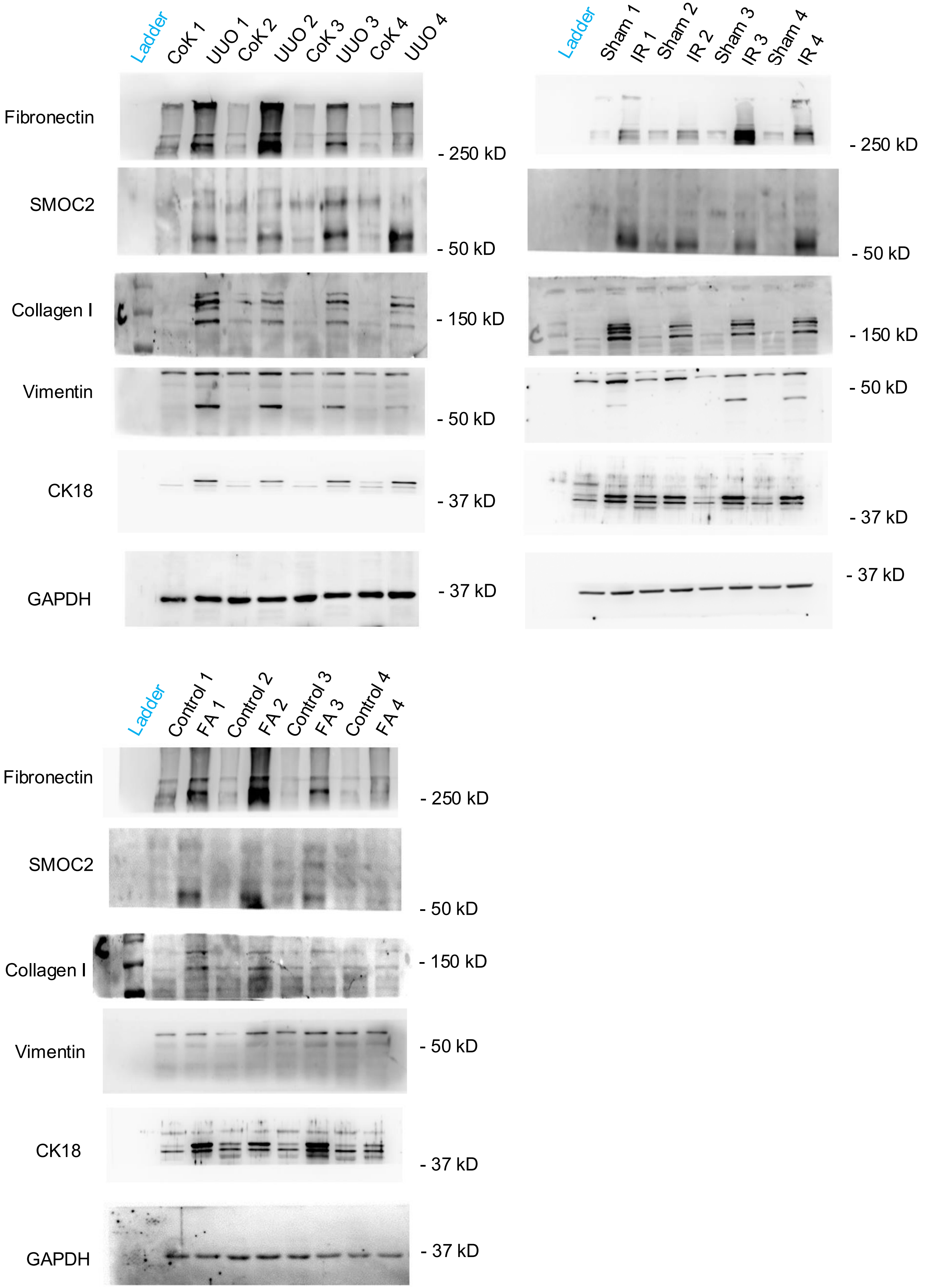
Ladder
CoK 1
UUO 1
CoK 2
UUO 2
CoK 3
UUO 3
CoK 4
UUO 4
Fibronectin
- 250 kD
SMOC2
- 50 kD
Collagen I
- 150 kD
Vimentin
- 50 kD
CK18
- 37 kD
GAPDH
- 37 kD
Ladder
Sham 1
IR 1
Sham 2
IR 2
Sham 3
IR 3
Sham 4
IR 4
- 250 kD
- 50 kD
- 150 kD
- 50 kD
- 37 kD
- 37 kD
Ladder
Control 1
FA 1
Control 2
FA 2
Control 3
FA 3
Control 4
FA 4
Fibronectin
- 250 kD
SMOC2
- 50 kD
Collagen I
- 150 kD
Vimentin
- 50 kD
CK18
- 37 kD
GAPDH
- 37 kD

Figure 4

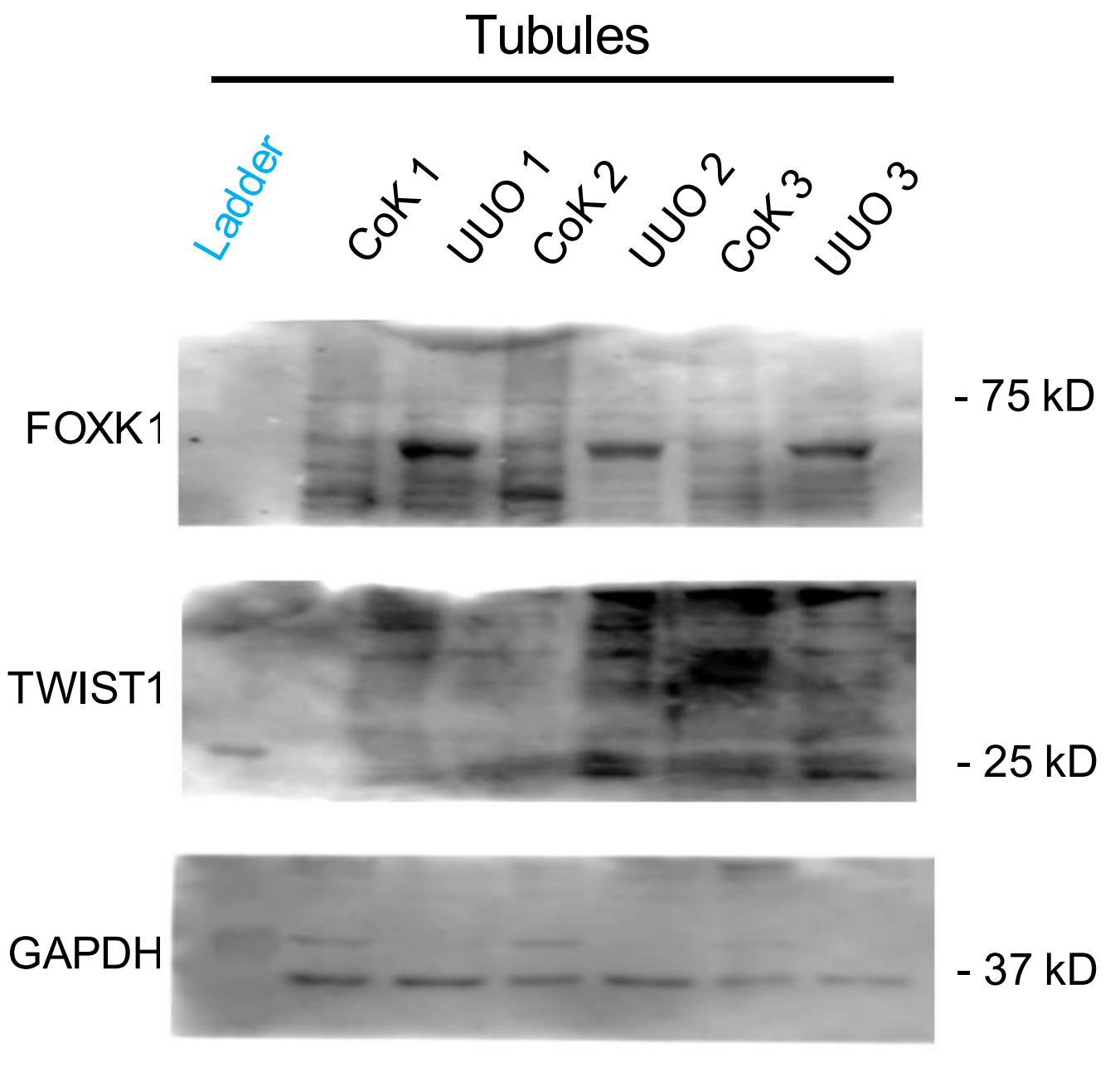
Tubules
Ladder
CoK 1
UUO 1
CoK 2
UUO 2
CoK 3
UUO 3
FOXK1
TWIST1
GAPDH
- 75 kD
- 25 kD
- 37 kD

Figures 7 and supplemental 3

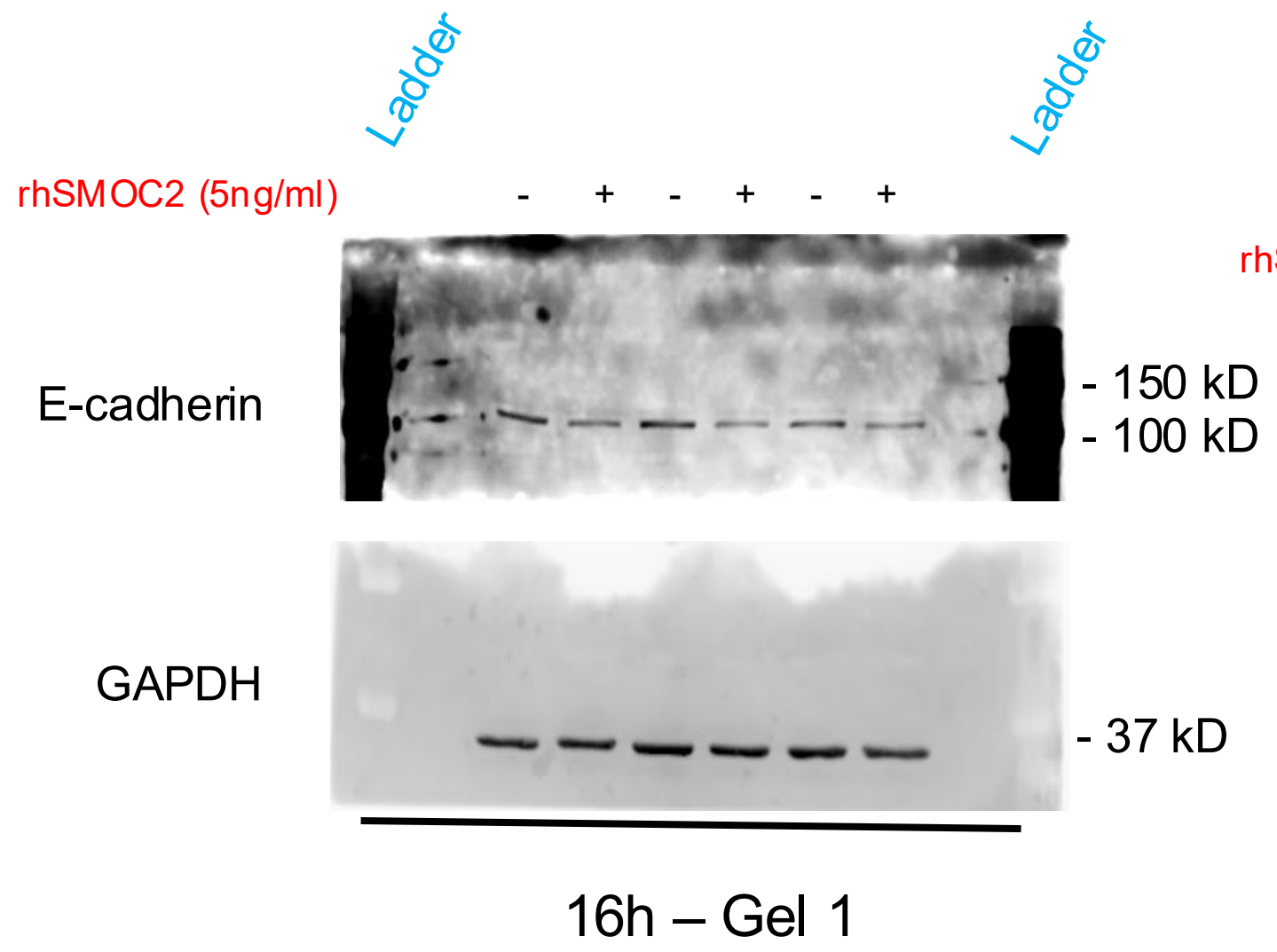

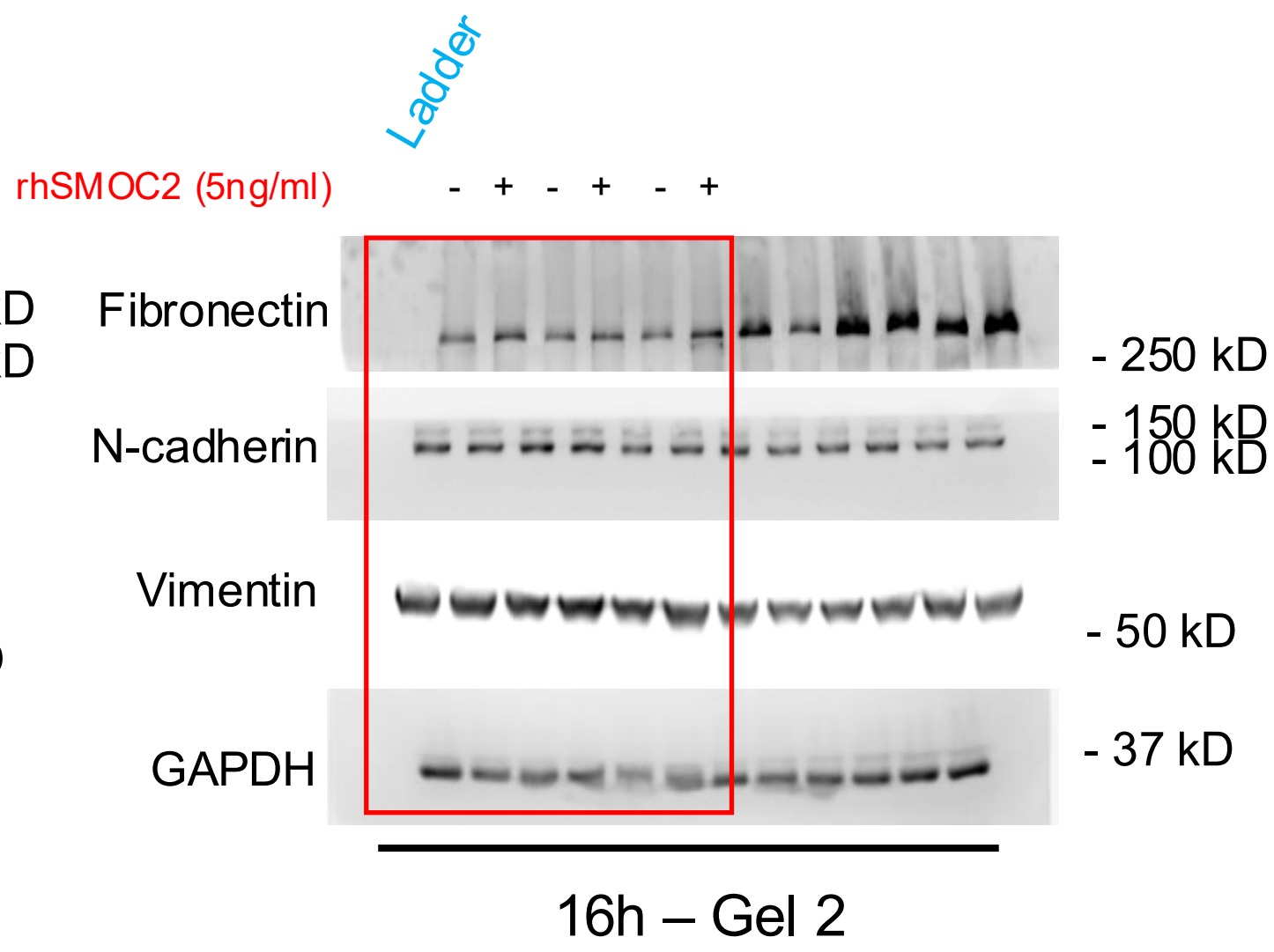

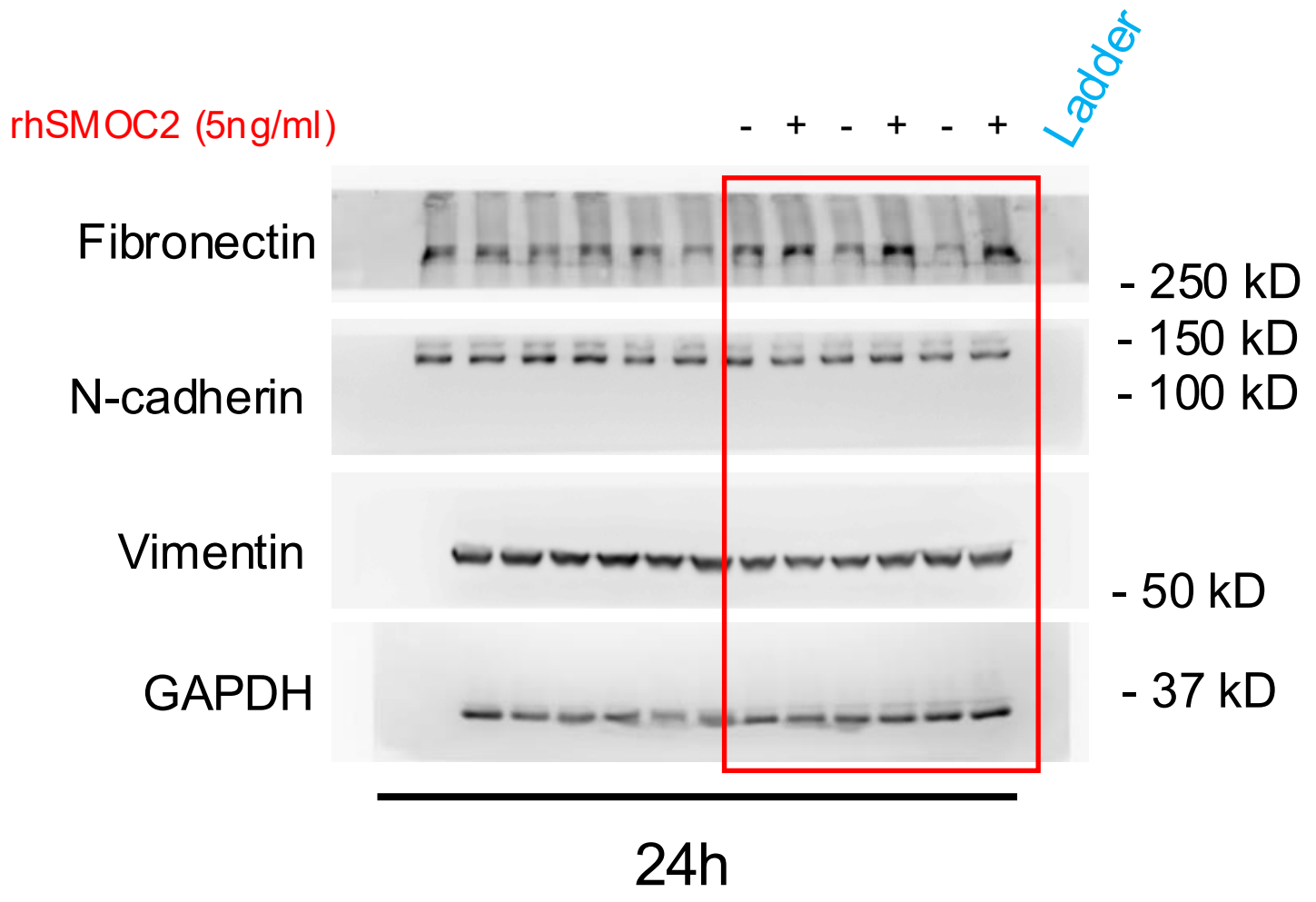

Figures 7 and supplemental 3 (cont.)

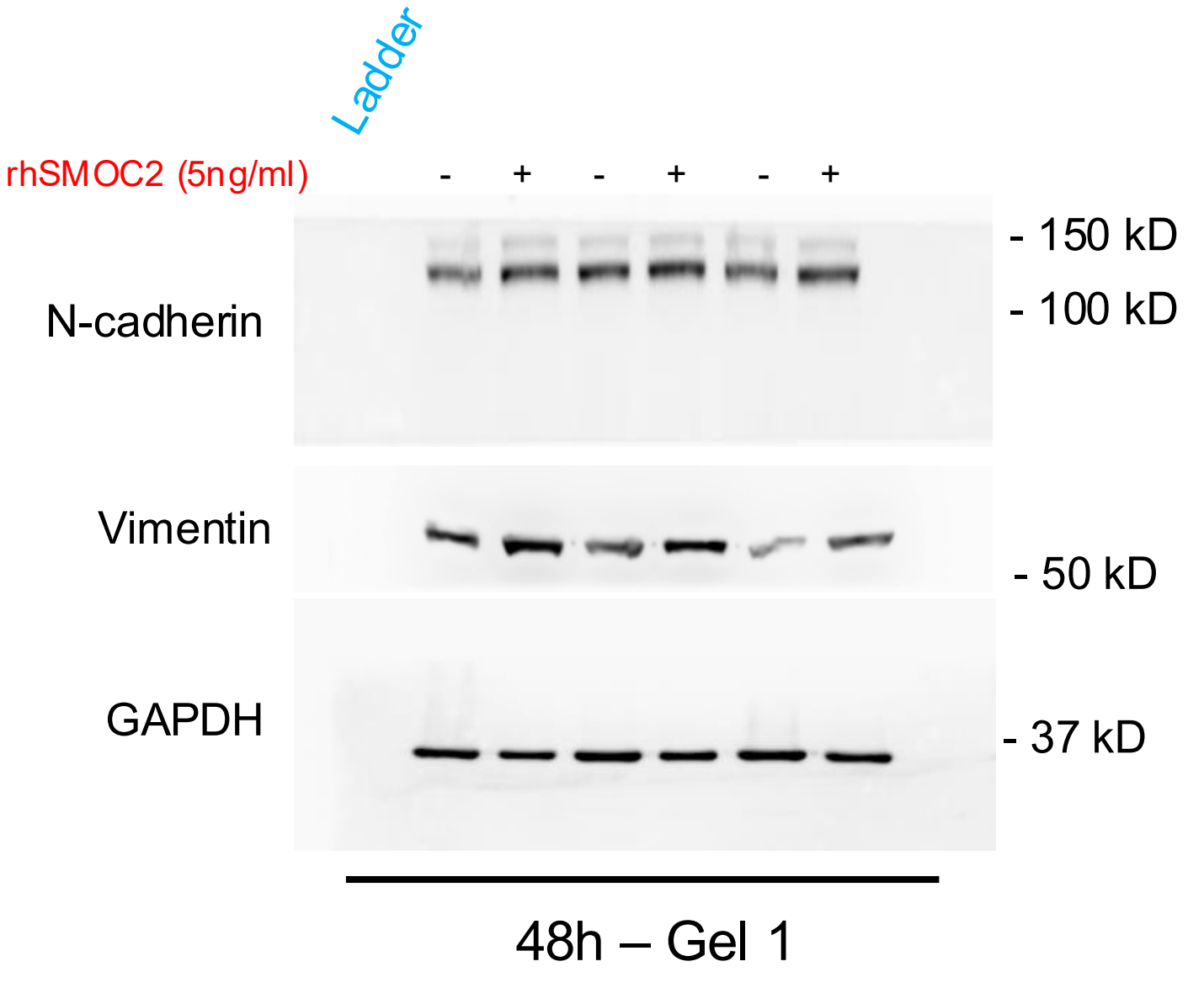

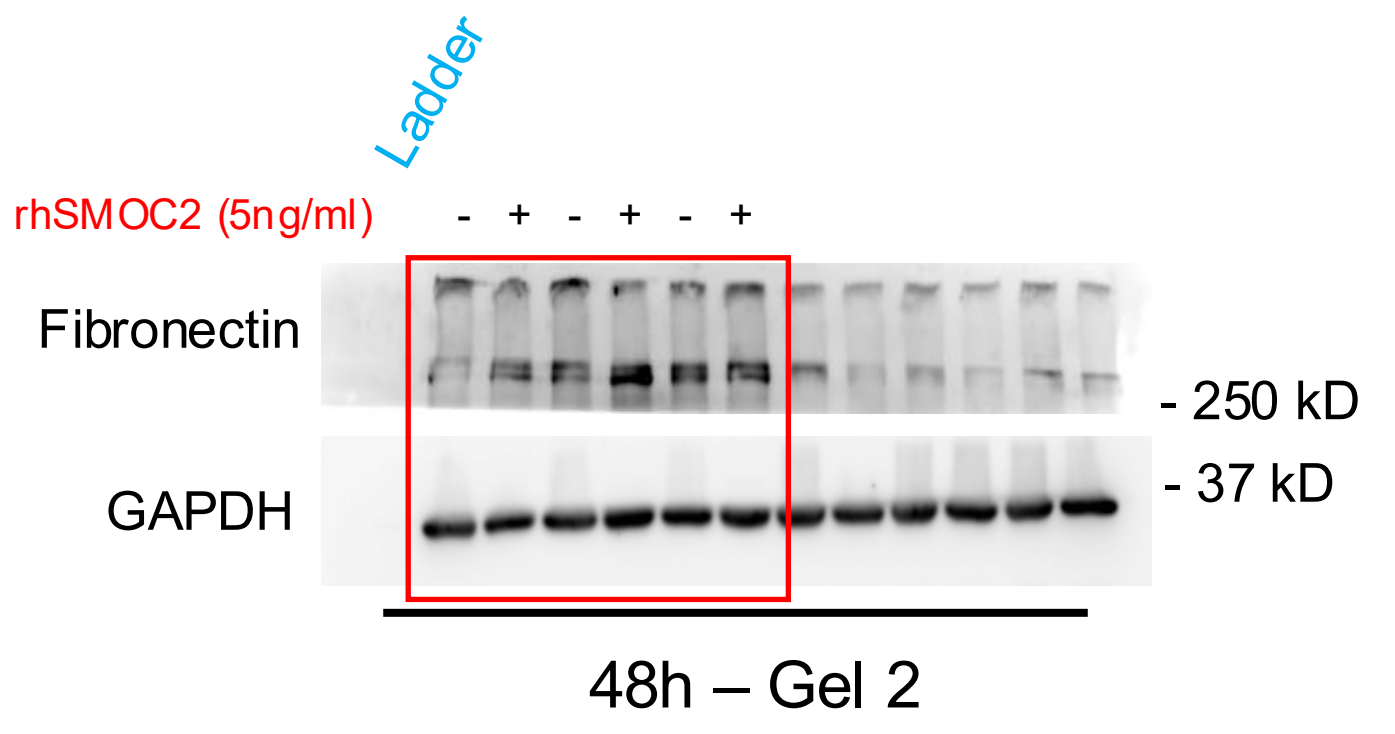

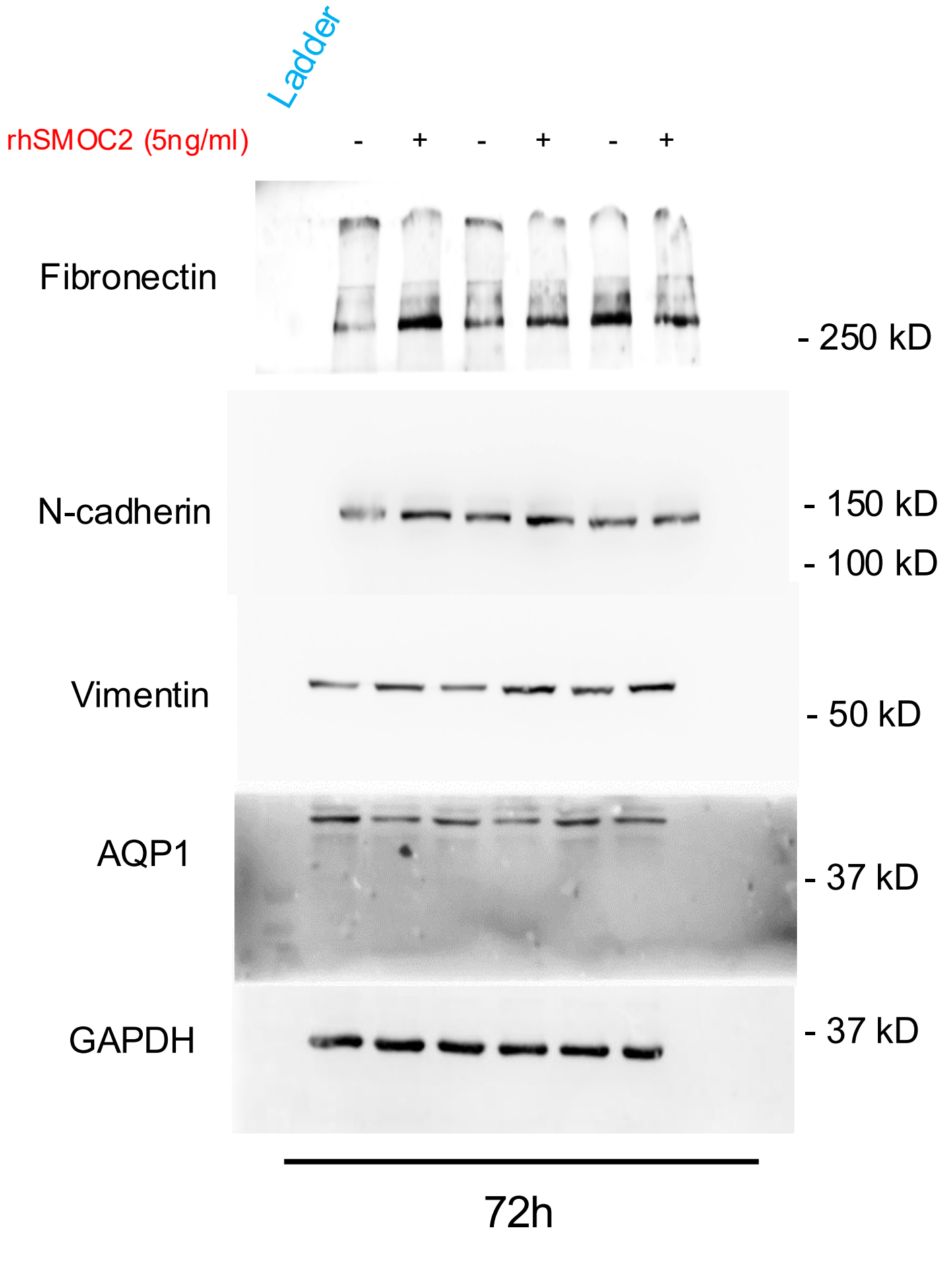

Figure 9

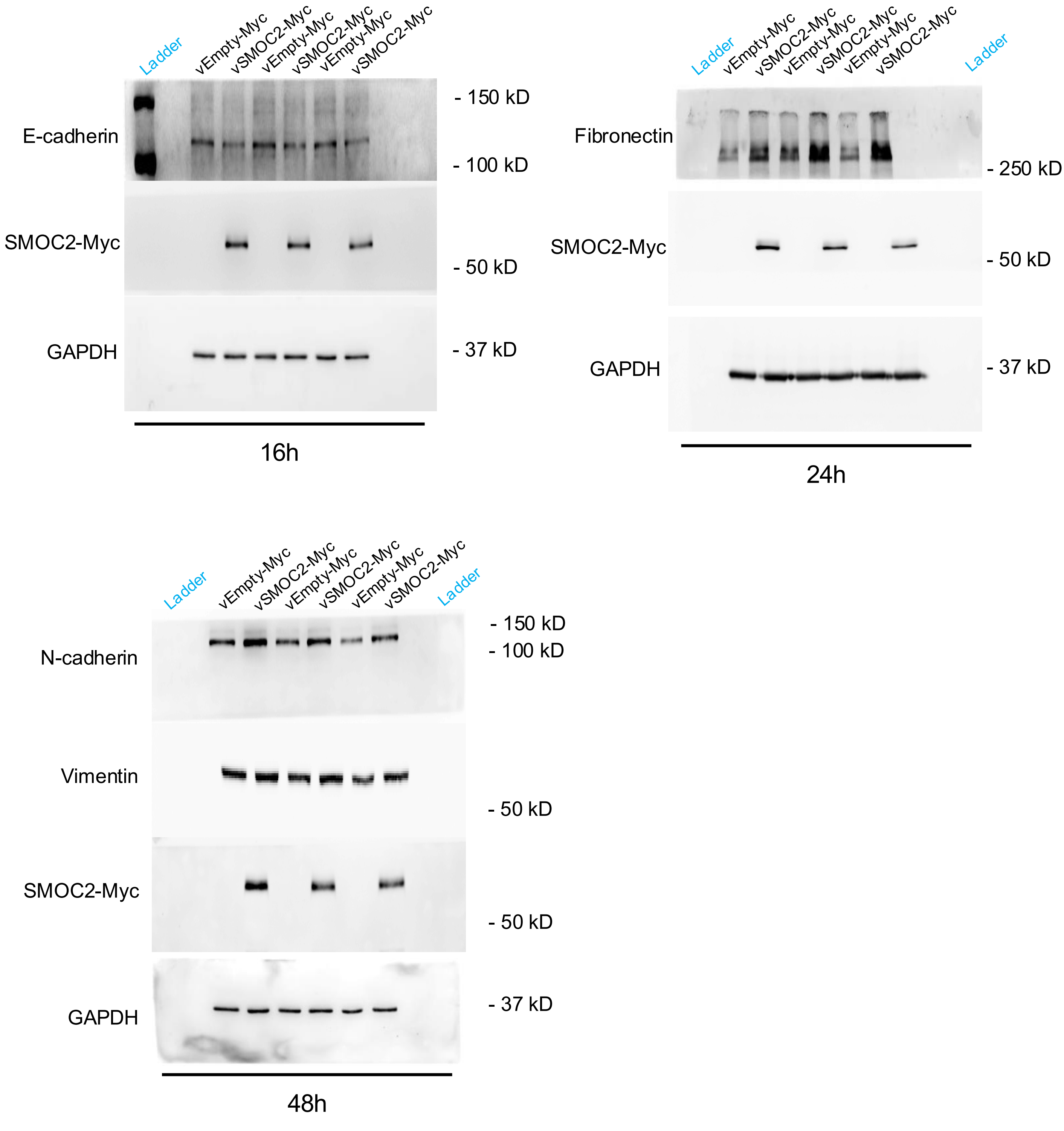

Ladder
vEmpty-Myc
vSMOC2-Myc
vEmpty-Myc
vSMOC2-Myc
vEmpty-Myc
vSMOC2-Myc
E-cadherin
SMOC2-Myc
GAPDH
- 150 kD
- 100 kD
- 50 kD
- 37 kD
16h
Ladder
vEmpty-Myc
vSMOC2-Myc
vEmpty-Myc
vSMOC2-Myc
vEmpty-Myc
vSMOC2-Myc
Ladder
Fibronectin
SMOC2-Myc
GAPDH
- 250 kD
- 50 kD
- 37 kD
24h
Ladder
vEmpty-Myc
vSMOC2-Myc
vEmpty-Myc
vSMOC2-Myc
vEmpty-Myc
vSMOC2-Myc
Ladder
N-cadherin
Vimentin
SMOC2-Myc
GAPDH
- 150 kD
- 100 kD
- 50 kD
- 50 kD
- 37 kD
48h

Figure 11

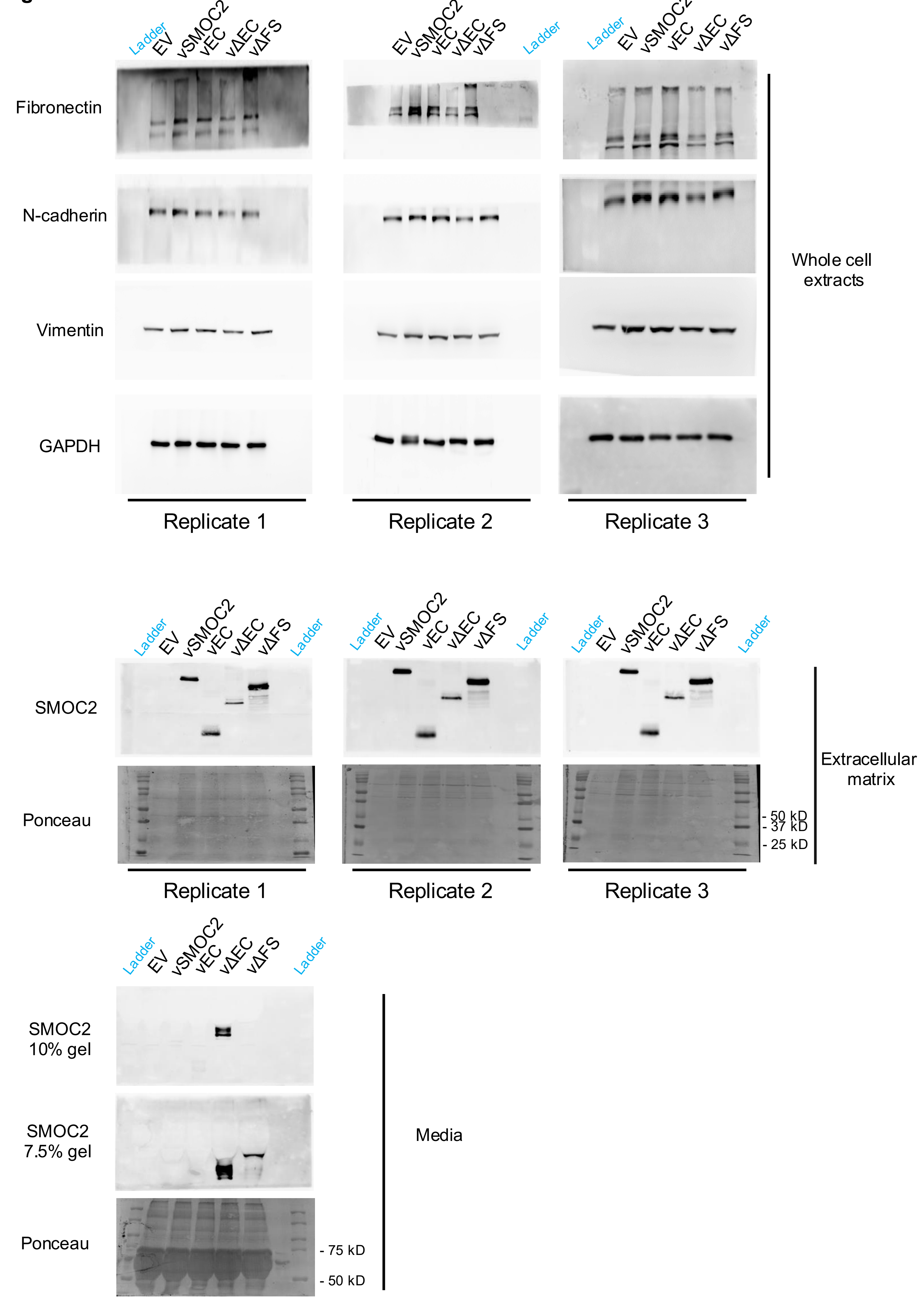

Ladder
EV
vSMOC2
vEC
vΔEC
vΔFS
Fibronectin
N-cadherin
Vimentin
GAPDH
Whole cell extracts
Replicate 1
Replicate 2
Replicate 3
SMOC2
Ponceau
Extracellular matrix
- 50 kD
- 37 kD
- 25 kD
SMOC2 10% gel
SMOC2 7.5% gel
Media
- 75 kD
- 50 kD

Figure 14

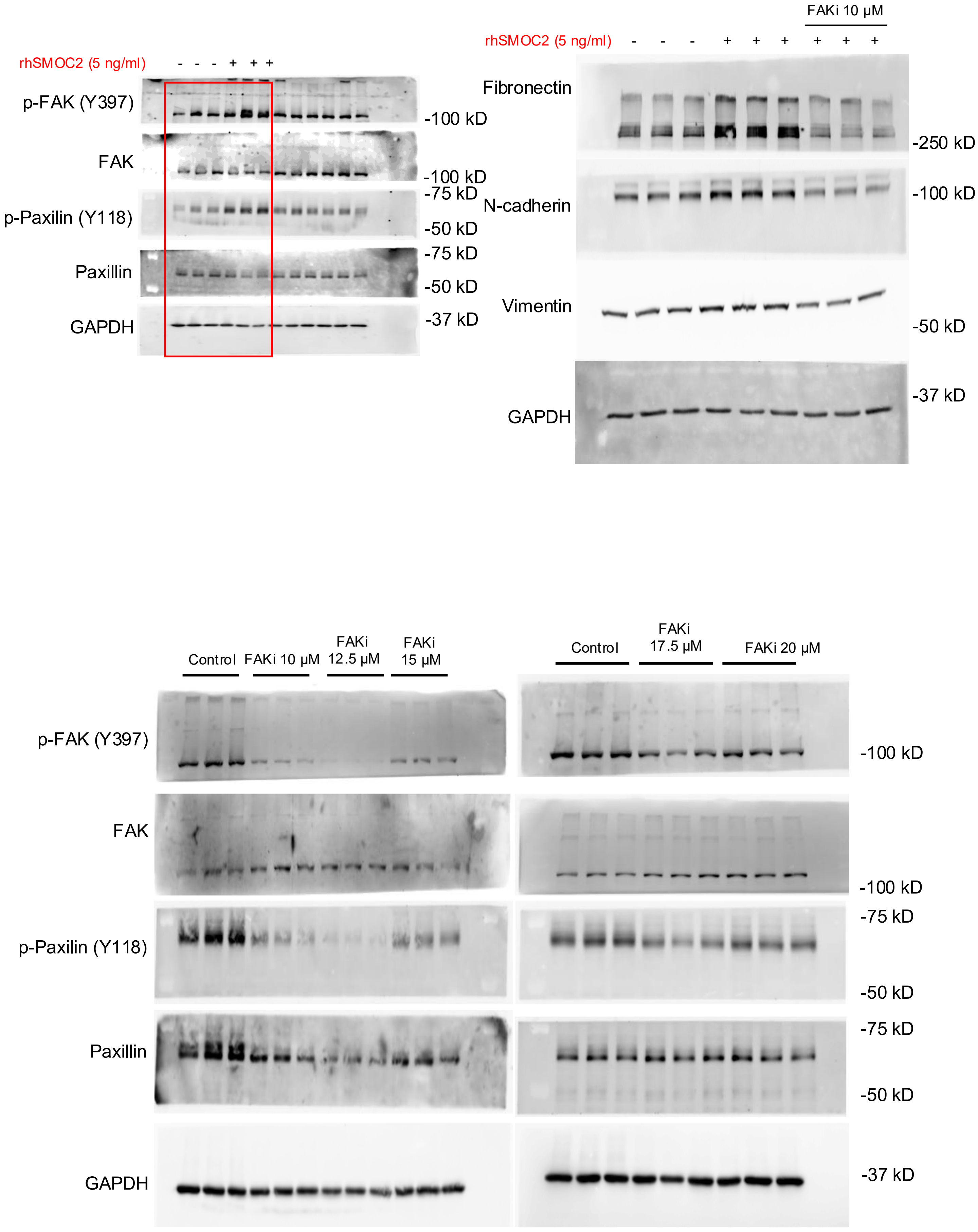

rhSMOC2 (5 ng/ml) - - - + + +
p-FAK (Y397)
FAK
p-Paxilin (Y118)
Paxillin
GAPDH
-100 kD
-100 kD
-75 kD
-50 kD
-75 kD
-50 kD
-37 kD
FAKi 10 µM
rhSMOC2 (5 ng/ml) - - - + + + + + +
Fibronectin
N-cadherin
Vimentin
GAPDH
-250 kD
-100 kD
-50 kD
-37 kD
Control
FAKi 10 µM
FAKi 12.5 µM
FAKi 15 µM
FAKi 17.5 µM
FAKi 20 µM
p-FAK (Y397)
FAK
p-Paxilin (Y118)
Paxillin
GAPDH
-100 kD
-100 kD
-75 kD
-50 kD
-75 kD
-50 kD
-37 kD

Figure 15

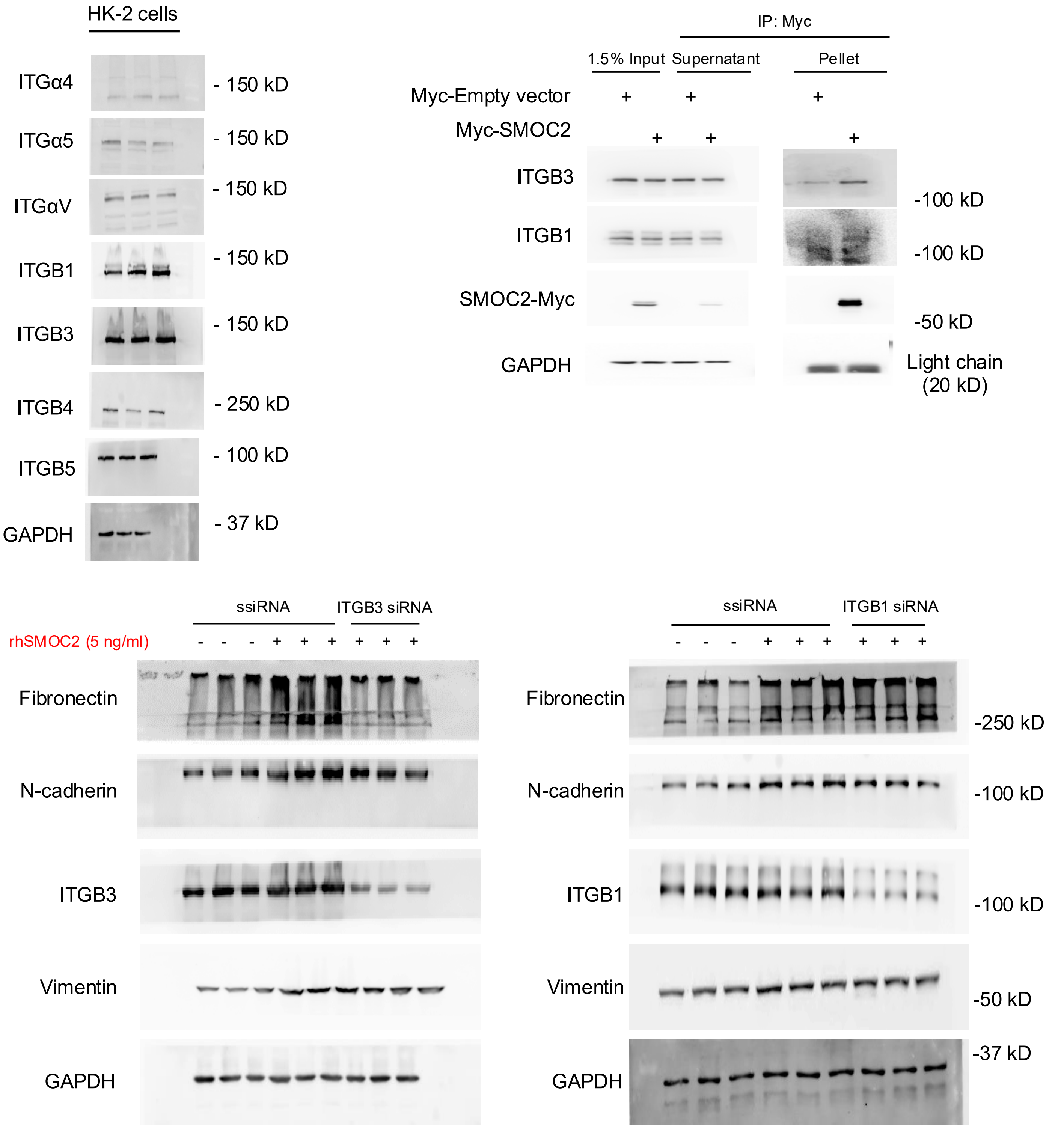
HK-2 cells
ITGα4
- 150 kD
ITGα5
- 150 kD
ITGαV
- 150 kD
ITGB1
- 150 kD
ITGB3
- 150 kD
ITGB4
- 250 kD
ITGB5
- 100 kD
GAPDH
- 37 kD
IP: Myc
1.5% Input
Supernatant
Pellet
Myc-Empty vector
Myc-SMOC2
ITGB3
ITGB1
SMOC2-Myc
GAPDH
-100 kD
-100 kD
-50 kD
Light chain (20 kD)
ssiRNA
ITGB3 siRNA
rhSMOC2 (5 ng/ml)
Fibronectin
N-cadherin
ITGB3
Vimentin
GAPDH
ssiRNA
ITGB1 siRNA
Fibronectin
N-cadherin
ITGB1
Vimentin
GAPDH
-250 kD
-100 kD
-100 kD
-50 kD
-37 kD

Figure 16

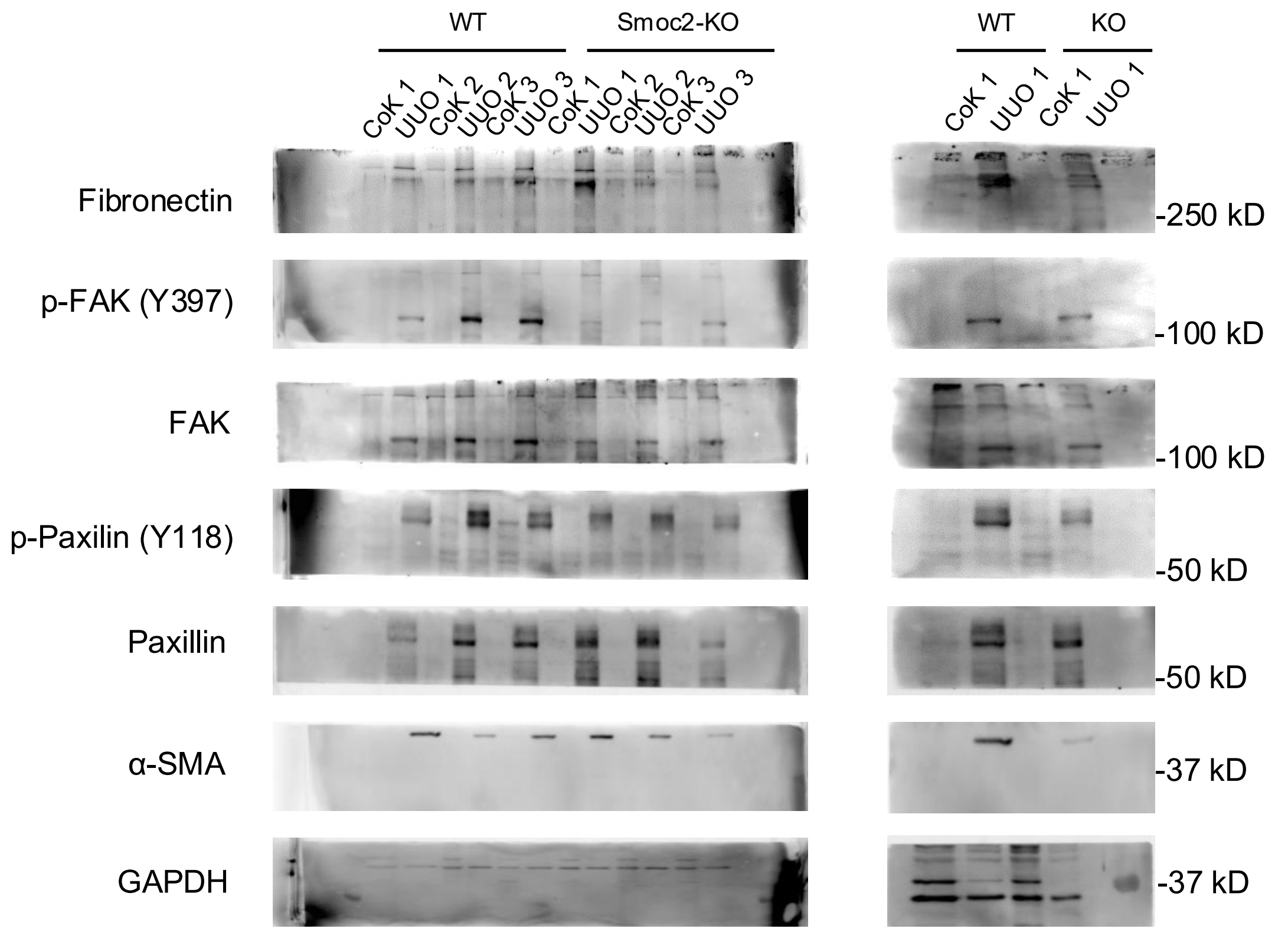

WT
Smoc2-KO
CoK 1
UUO 1
CoK 2
UUO 2
CoK 3
UUO 3
CoK 1
UUO 1
CoK 2
UUO 2
CoK 3
UUO 3
WT
KO
CoK 1
UUO 1
CoK 1
UUO 1
Fibronectin
p-FAK (Y397)
FAK
p-Paxilin (Y118)
Paxillin
α-SMA
GAPDH
-250 kD
-100 kD
-100 kD
-50 kD
-50 kD
-37 kD
-37 kD

Figure 17

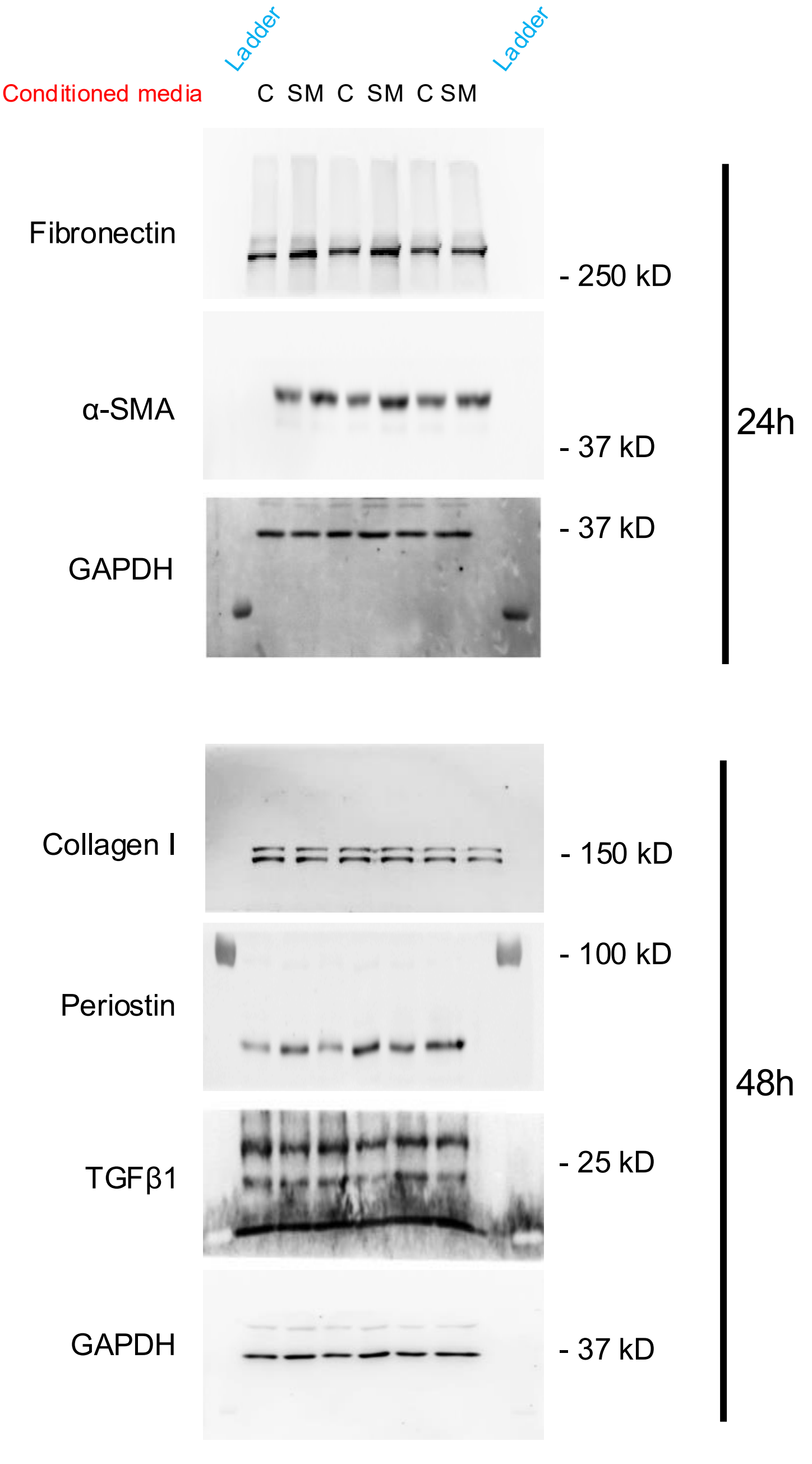
Ladder
Ladder
Conditioned media
C SM C SM C SM
Fibronectin
- 250 kD
α-SMA
- 37 kD
GAPDH
- 37 kD
24h
Collagen I
- 150 kD
Periostin
- 100 kD
TGFβ1
- 25 kD
GAPDH
- 37 kD
48h

Supplemental 4

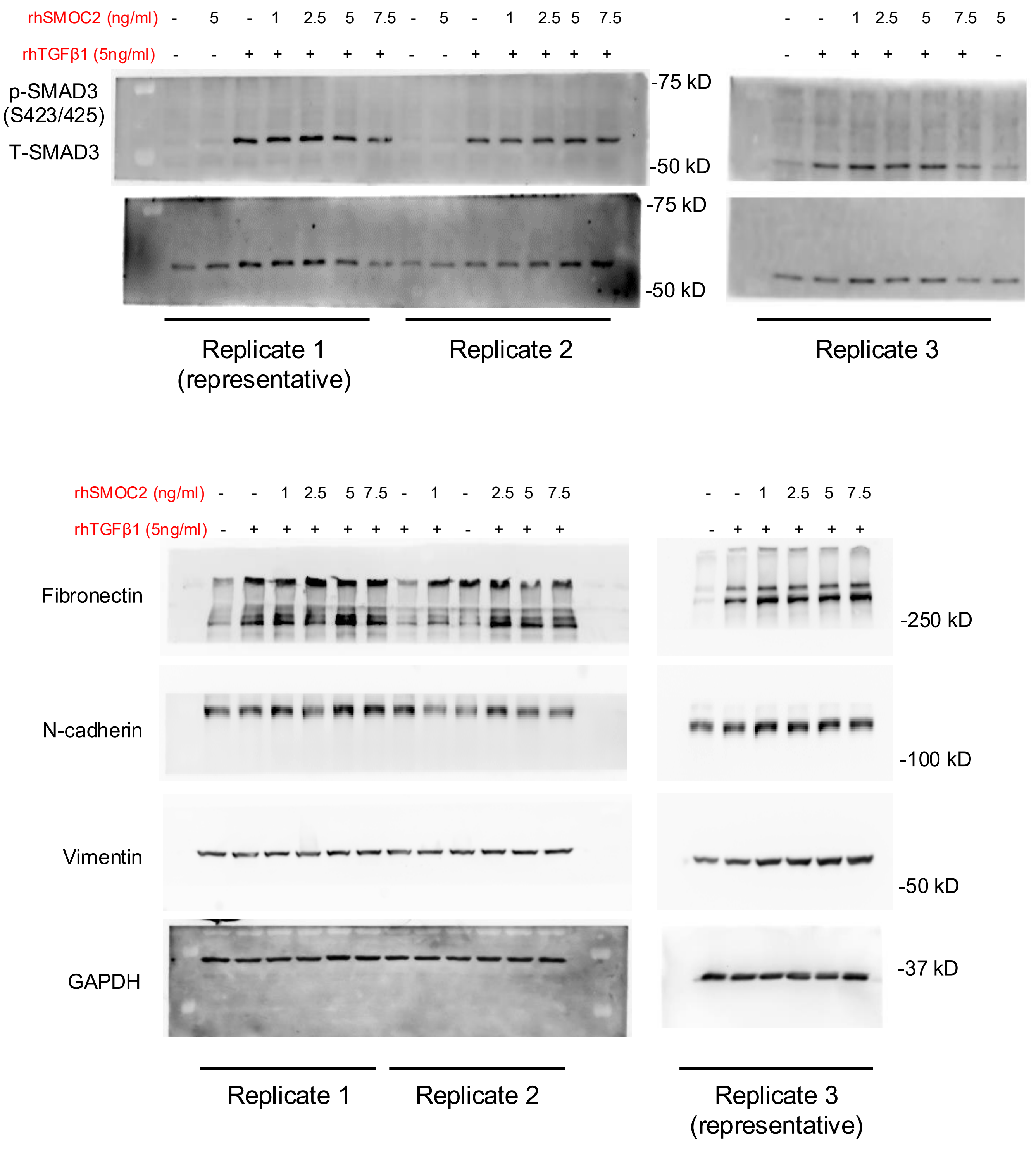

Supplemental 6

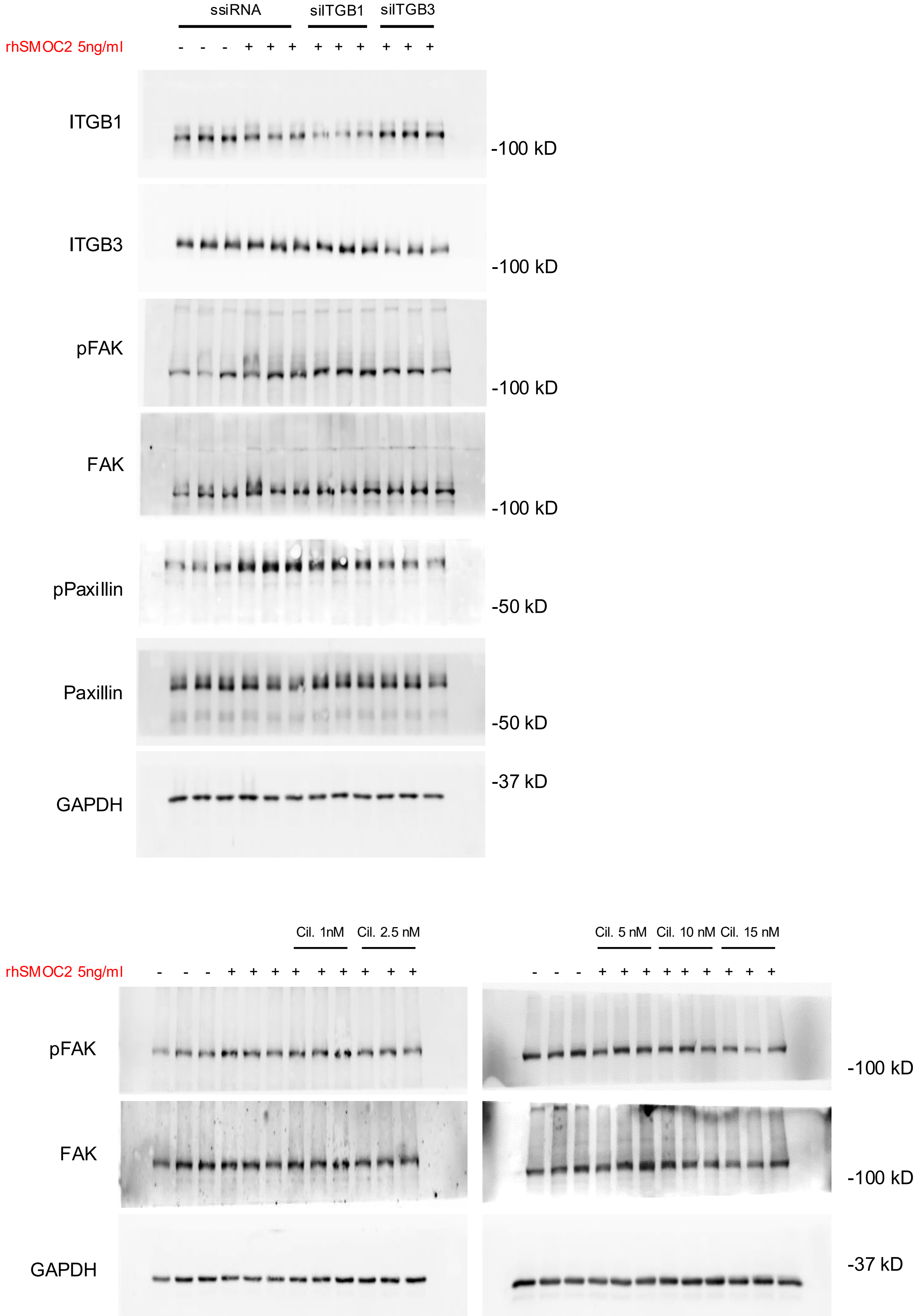

Supplement: Supplementary file 2 [file jciinsight-11-198555-s042.pdf]
